# Small organic molecules with tailored structures: initiators in the transition-metal-free C–H arylation of unactivated arenes†

**DOI:** 10.1039/d0ra01845g

**Published:** 2020-04-09

**Authors:** Zhenghui Liu, Peng Wang, Yu Chen, Zhenzhong Yan, Suqing Chen, Wenjun Chen, Tiancheng Mu

**Affiliations:** School of Pharmaceutical and Materials Engineering, Taizhou University Taizhou 318000 Zhejiang China liuzhenghui@iccas.ac.cn; Beijing National Laboratory for Molecular Sciences, CAS Research/Education Center for Excellence in Molecular Sciences, Institute of Chemistry, Chinese Academy of Sciences Beijing 100190 China; Key Laboratory of Green Chemical Media and Reactions, Ministry of Education, School of Chemistry and Chemical Engineering, Henan Normal University Xinxiang 453007 Henan China; Department of Chemistry and Material Science, Langfang Normal University Langfang 065000 Hebei China; Department of Chemistry, Renmin University of China Beijing 100872 China tcmu@ruc.edu.cn

## Abstract

Simple, small organic molecules containing nitrogen and oxygen atoms in their structures have been disclosed to catalyze transition-metal-free C–H arylation of unactivated arenes with aryl iodides in the presence of ^*t*^BuOK. In this article, an optimized catalytically active molecule, (2-(methylamino)phenyl)methanol, was designed. A broad range of aryl iodides could be converted into the corresponding arylated products at 100 °C over 24 h with good to excellent yields. Mechanistic experiments verified that radicals participated in this catalytic transformation and that the cleavage of the aromatic C–H bond was not the rate determining step. A K^+^ capture experiment by 18-crown-6 emphasized the significance of the cation species of the strong base. Fourier transform infrared spectroscopy proved that the catalytic system was activated by the hydrogen bonds between small organic molecules and ^*t*^BuOK. Also, a clear mechanism was proposed. This transition-metal-free method affords a promising system for efficient and inexpensive synthesis of biaryls *via* a user-friendly approach, as confirmed by scale-up experiments.

## Abstract 2

A small organic molecule was tailored for the efficient synthesis of biphenyl and its derivatives from aryl iodides.

## Graphical abstract

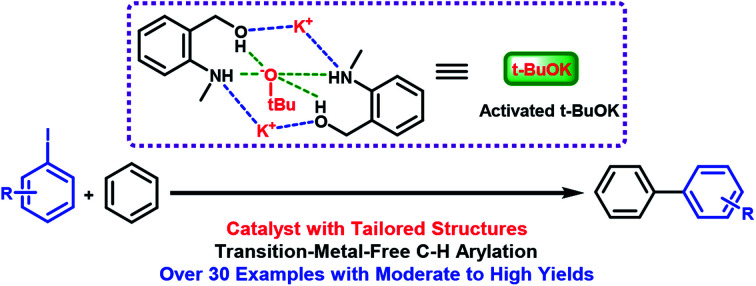

## 1. Introduction

**Scheme 1:**
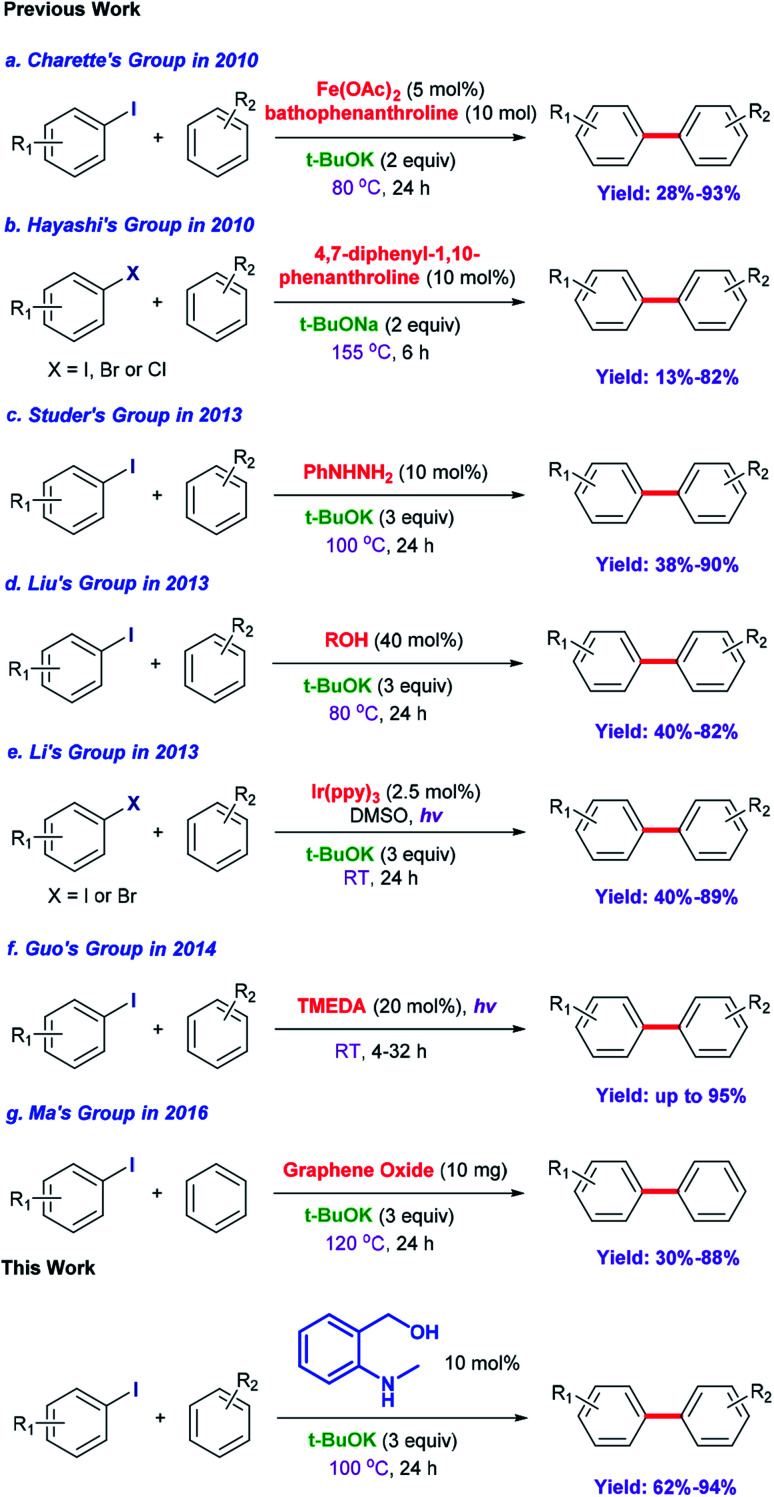
Synthesis of biaryls and their derivatives from aryl halides and unactivated arenes.

Biaryl compounds are a privileged class of structures; their use is emerging in a variety of pharmaceuticals, natural products, agrochemicals, ligands for chemical conversion and functional materials.^1,2^ Preparation of biaryl compounds from arylation of arenes has been paid much attention in recent years.^3–7^ In earlier years, transition metals were employed in these transformations, such as Fe,^8^ Co,^9^ Ni,^10–12^ Cu,^13–18^ Pd,^19–23^ Ru,^24,25^ Rh^26^ and Ir.^27,28^ In 2010, Charette *et al.* completed the arylation of aryl iodides catalyzed by Fe(OAc)_2_ with the ligand bathophenanthroline and ^*t*^BuOK at 155 °C after 16 h (yields: 28% to 93%) (Scheme 1a).^29^ Considering their environmental protection ability and catalytic cost, organocatalysts are attracting increasing attention and interest from synthetic chemists as effective alternatives to traditional transition-metal-based catalysts. Hayashi's group set up a catalytic system based on 1,10-phenanthroline with different substituent groups, and yields of 65–82% (aryl iodide substrates) were obtained after only 6 h at 155 °C. Interestingly, this system could also be applied to aryl bromides (13–80%) and aryl chlorides (75%). In addition, ^*t*^BuONa was used as a strong alkali in the reactions; it is seldom employed in such transformations (Scheme 1b).^30^ In 2013, Studer *et al.* employed commercially available and inexpensive phenyl hydrazine as an effective initiator to translate aryl iodides into cross-coupling products (yields of 38–90%, 100 °C and 24 h) (Scheme 1c).^31^ After that, an oxygen-based system was proposed by Liu's group in which a series of simple aliphatic alcohols performed well, especially *n*-BuOH, affording products in yields of 40–82% (Scheme 1d).^32^ In addition, a set of photocatalysis methods were proposed in which Li's group and Guo's group utilized Ir(ppy)_3_ (under visible-light irradiation with ^*t*^BuOK in DMSO at room temperature; the yields were 40–89%)^33^ and TMEDA (under UV-light irradiation with no strong base at room temperature, yields up to 95%)^34^ to achieve the synthesis of biaryls, respectively (Scheme 1e and f). In 2016, Ma *et al.* explored nanocarbon-material (graphene oxide) catalytic systems, and the catalytic activities of various model compounds were compared with charge analysis. Moreover, DFT calculations gave specific information about the mechanism (Scheme 1g).^35^

Prompted by the aforementioned and other related literature reports,^36–43^ herein, we propose a catalytic system composed of a small organic molecule ((2-(methylamino)phenyl)methanol, abbreviated as SOM in this article) and a strong base (^*t*^BuOK) for the synthesis of biphenyl and its derivatives (good to excellent yields, broad substrate scope and good functional group tolerance) from arenes and aryl iodides at 100 °C *via* a single electron transfer (SET) process. The catalytically active molecule with a specifically tailored structure was designed and could be applied to scale-up experiments, showing potential for industrial production. Radical trapping, crown ether and kinetic isotope experiments gave deep insights into the reaction mechanism.

## 2. Results and discussion

In an initial set of experiments, 14 selected small organic molecules with nitrogen or oxygen atoms in their structures were tested for the coupling of benzene and iodobenzene according to previous studies and our previous work in this area (as shown in Table 1).^31,44–49^ All the tested molecules showed catalytic ability for the conversion except (CH_3_)_3_OH, which creates extremely large steric hindrance (Table 1, entry 11). Aliphatic diamines exhibited considerable yields, especially after mono-methylation (Table 1, entries 1–3, 65–71%). Also, PhNHCH_3_ showed higher yield (82%, Table 1, entry 4). O atoms in the form of carbonyl groups (whether conjugated or not) could only provide biaryls in yields below 50% (Table 1, entries 5 and 6). Methanol and ethanol supplied coupling products in yields of 31% and 38%, respectively (Table 1, entries 7 and 8). Further lengthening of the carbon chain led to only slightly improved yield (Table 1, entries 9 and 10). To our delight, the introduction of a benzene ring increased the yield, probably due to π, π-stacking interactions between the substrates and catalytically active molecules (Table 1, entries 12–14).^49^ In addition, prolonging the side chains did not help improve the yield.

**Table 1:** Explorations of the effects of N and O in initiators

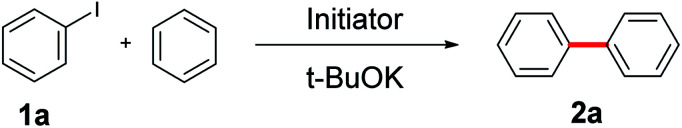		
Entry	Initiator^a^	Yield/%^b^
1	H_2_NCH_2_CH_2_NH_2_	65
2	H_2_NCH_2_CH_2_CH_2_NH_2_	68
3	H_3_CNHCH_2_CH_2_NHCH_3_	71
4	PhNHCH_3_	82
5	PhCOCH_3_	32
6	PhCH_2_COCH_3_	44
7	CH_3_OH	31
8	CH_3_CH_2_OH	38
9	CH_3_(CH_2_)_2_OH	41
10	CH_3_(CH_2_)_3_OH	45
11	(CH_3_)_3_OH	3
12	PhCH_2_OH	56
13	PhCH_2_CH_2_OH	43
14	PhCH_2_CH_2_CH_2_OH	42

a Reaction conditions: 1a (0.4 mmol), benzene (4 mL), catalyst (10 mol%), ^*t*^BuOK (1.2 mmol), 100 °C, 24 h, unless otherwise stated.

b Yields were determined by GC using *n*-dodecane as an internal standard.

After the explorations mentioned above, the effects of N and O atoms in the catalyzed coupling reactions were preliminarily disclosed. Then, the optimized molecules were screened. Because PhNHCH_3_ and PhCH_2_OH showed the highest yields among N- and O-containing catalyst molecules, respectively, molecules were designed that contained these groups. As shown in Table 2 and 3 molecules containing the two substituent groups in the *o*-, *m*-, and *p*-positions were tested. The *m*- and *p*-substituted molecules seemed to have no synergistic effects, with lower yields than PhNHCH_3_ alone. Without doubt, (2-(methylamino)phenyl)methanol (abbreviated as SOM) was the optimal catalyst molecule in this research.

**Table 2:** Explorations of combinations and molecular structures of effective initiators

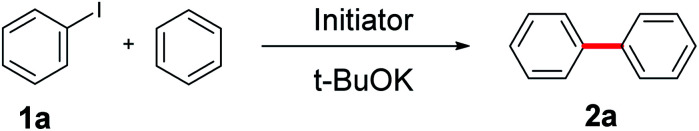		
Entry	Initiator^a^	Yield/%^b^
1	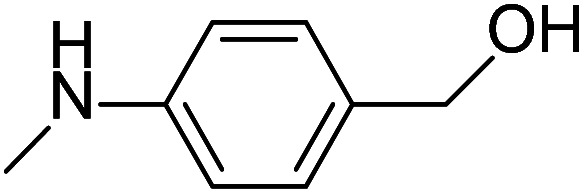	74
2	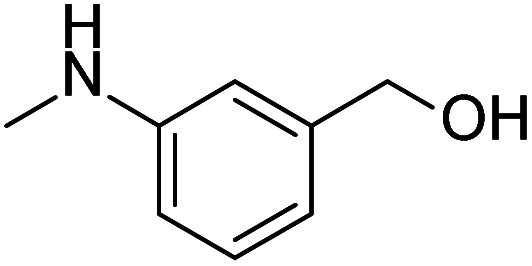	80
3	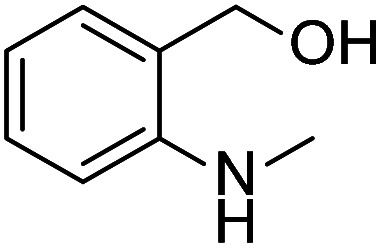	92^c^^,^^d^

a Reaction conditions: 1a (0.4 mmol), initiator (10 mol%), ^*t*^BuOK (1.2 mmol), benzene (4 mL), 100 °C, 24 h.

b Yields were determined by GC using *n*-dodecane as an internal standard.

c 4% yield for bromobenzene.

d 0% yield for chlorobenzene.

**Table 3:** Radical trapping experiments

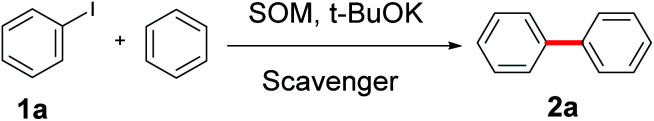		
Entry	Scavenger	Yield (%)^a^
1	None	92
2	1,1-Diphenylethylene (1.0 equiv.)	<1
3	TEMPO (1.0 equiv.)	<1
4	1,4-Benzoquinone (2.0 equiv.)	<1
5	TBA^+^PF_6_^−^ (2.0 equiv.)	<1

a Yields were determined by GC using dodecane as an internal standard. The structures of the radical scavengers are shown below:
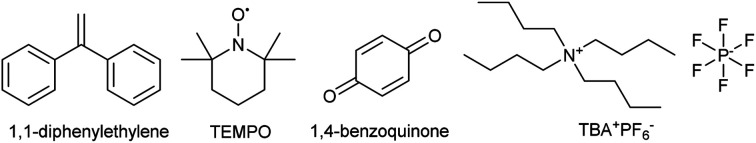

Various bases were studied, and the results are shown in Fig. 1a. Consistent with previous reports,^35,50–52^ only ^*t*^BuOK was effective for the transformation, and even ^*t*^BuONa and ^*t*^BuOLi were both invalid. This implies that K^+^ plays an important role in the cross-coupling reaction. In addition, other weaker bases, such as KOH, K_2_CO_3_ and K_3_PO_4_, provided no yield.

**Fig. 1:**
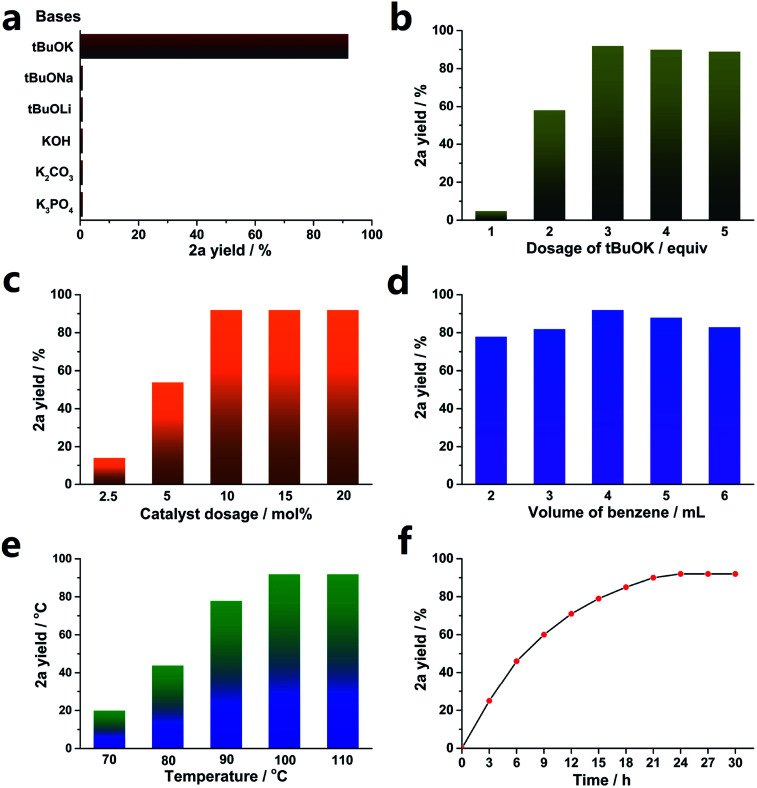
SOM-catalyzed C–H arylation of benzene with iodobenzene. (a) Effects of bases. (b) Dosage of ^*t*^BuOK, (c) catalyst dosage, (d) volume of benzene, (e) effects of temperature, (f) dynamic tests. Standard reaction conditions: PhI 0.4 mmol, benzene 4 mL, ^*t*^BuOK 1.2 mmol, SOM 10 mol%, 100 °C, 24 h, unless otherwise stated; yields were determined by GC using dodecane as an internal standard.

Then, the optimized dosage of ^*t*^BuOK was explored (exhibited in Fig. 1b). 3 equiv. ^*t*^BuOK was the most suitable dosage, while less ^*t*^BuOK led to a sharp decrease of the yield of 2a. Also, a larger dosage could not provide a higher yield.

A low concentration of the catalyst (SOM) could not promote the transformation sufficiently. As demonstrated in Fig. 1c, SOM above 10 mol% (relative to 1a) was enough to achieve the highest yield. As expected, 5 mol% and even 2.5 mol% could not supply enough catalyst concentration, leading to decreased yields.

Benzene acted as both a substrate and solvent here. Less benzene could not provide sufficient arene substrate dosage, while more benzene led to dilution of the catalyst concentration. Therefore, we explored the optimized benzene volume, and the results are shown in Fig. 1d. Obviously, 4 mL benzene was appropriate for the reaction.

Temperature had an important effect on the reaction yields; sometimes, it made the difference between full conversion and no reaction.^53–55^ As shown in Fig. 1e, a lower temperature led to a dramatically decreased yield, and 100 °C was high enough to obtain the highest yield. In addition, increasing the temperature to 110 °C did not improve the yield.

A reaction dynamics study was carried out, and the results are exhibited in Fig. 1f. The reaction rate gradually reduced as the reaction went on, and 24 h was enough to afford the highest yield. Further increasing the reaction time by another 3 h or 6 h did not provide more products.

**Scheme 2:**
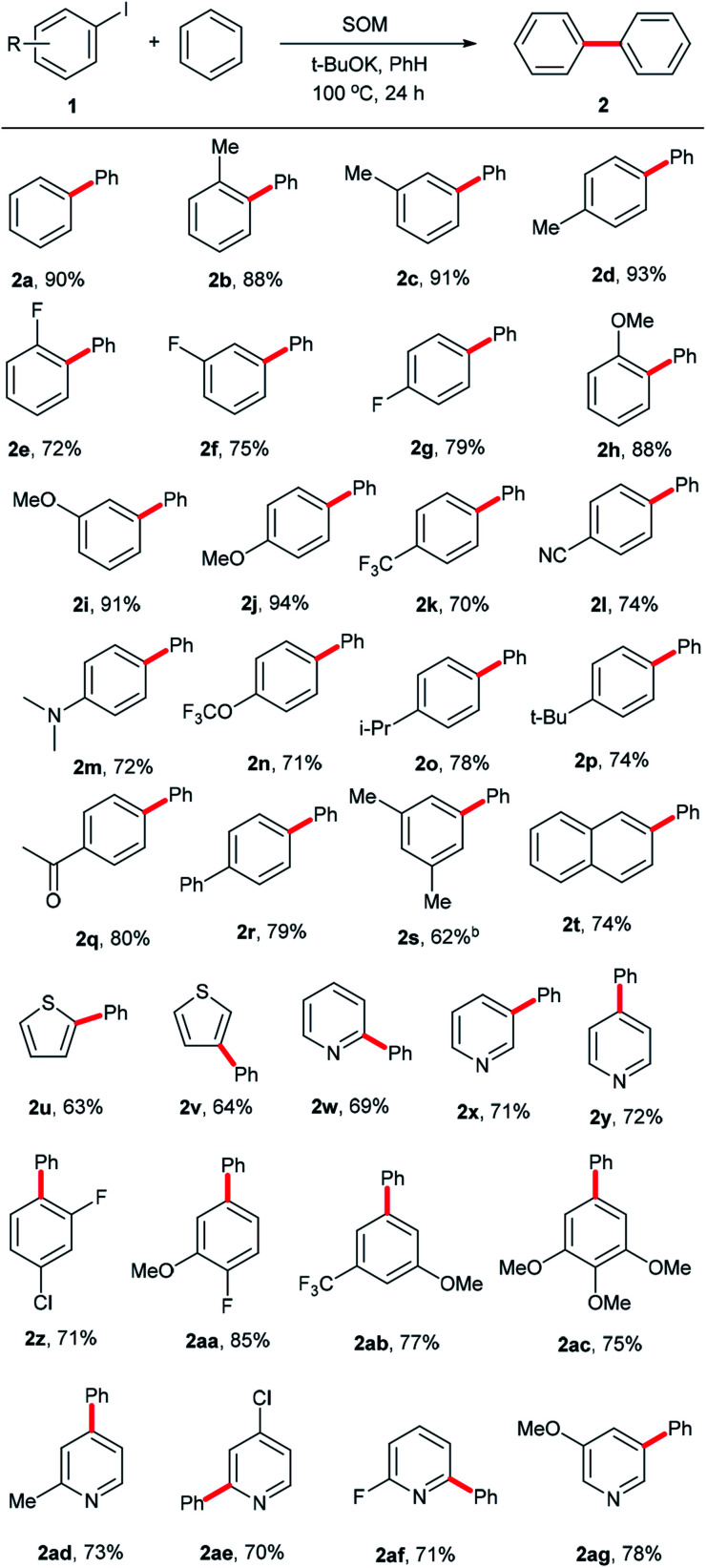
Substrate scope. ^a^Reaction conditions: 1 (0.4 mmol), SOM (10 mol%), ^*t*^BuOK (1.2 mmol), benzene (4 mL), 100 °C, 24 h, unless otherwise stated; ^b^48 h; ^c^isolated yields.

Having ascertained the feasibility of biphenyl synthesis from benzene and iodobenzene catalyzed by the SOM/^*t*^BuOK system, the substrate scope of this conversion was then explored (summarized in Scheme 2). Generally speaking, aryl iodide substrates bearing electron-donating substituent groups were relatively more active than those with electron-neutral or electron-withdrawing substituent groups. *Para-*substituent groups in the aryl iodide substrates afforded higher yields compared to those with *meta-* or *ortho-*substituents due to steric hindrance effects. For example, 94% yield of 2j was acquired using 1-iodo-4-methoxybenzene (1j) as an arylation reagent, while the reactivities of 1-fluoro-4-iodobenzene (1g) and 1-(4-iodophenyl)ethan-1-one (1q) were much lower, affording 79% yield of 2g and 80% yield of 2q, respectively. Regarding the steric hindrance effects, the couplings of benzene with 1-iodo-4-isopropylbenzene (1o) and 1-*tert*-butyl-4-iodobenzene (1p) were less efficient due to the large volumes of isopropyl and *tert*-butyl substituents, affording 78% yield of 2o and 74% yield of 2p. Comparatively, only 70% yield of 2k was obtained owing to a combination of steric and electronic effects of the trifluoromethyl group in 1k. For Me-, F- and MeO-substituent groups in different positions, yields of 88% (2b, *o*-Me) < 91% (2c, *m*-Me) < 93% (2d, *p*-Me); 72% (2e, *o*-F) < 91% (2f, *m*-F) < 93% (2g, *p*-F); and 72% (2h, *o*-OMe) < 91% (2i, *m*-OMe) and < 93% (2j, *p*-OMe) were obtained. 2l, 2n and 2q with electron-withdrawing substituent groups were obtained with yields of 74%, 71% and 80%, respectively. 2r, 2s and 2t with high steric hindrance were obtained with yields of 79%, 62% and 74%, respectively. Aromatic heterocyclic substrates tended to afford relatively lower yields than iodobenzene (1a), with yields of 2u-2v (63–64%) and 2w–2y (69–72%). 2y was obtained in slightly higher yield, perhaps owing to the tendency toward regular arrangement of the symmetrical structure. In order to establish this system as a versatile approach to obtain more biphenyl derivatives, some multi-substituted phenyl iodides and heterocyclic iodides with potential in medicinal chemistry applications were employed as substrates. For the drolated yields were obtained. Then, we attempted to test some substituted heterocyclic iodides; 2ad–2ag were afforded in yields of 70–78%. These results show that the established catalytic system is a promising approach to obtain more valuable or complicated compounds.

In order to explore whether radicals were involved in the transformation and to verify the SET mechanism, a set of radical trapping experiments were conducted. As shown in Table 3, addition of radical scavengers such as 1,1-diphenylethylene (1.0 equiv.), TEMPO (1.0 equiv.) or 1,4-benzoquinone (2.0 equiv.) could change the yield from 92% to below 1% due to the consumption of single electrons. In addition, the reaction was terminated by TBA^+^PF_6_^−^, which confirmed the existence of ion radicals. Therefore, radicals were proved to participate in the reaction process, in line with previously reported arylation reactions.^56–61^

**Scheme 3:**
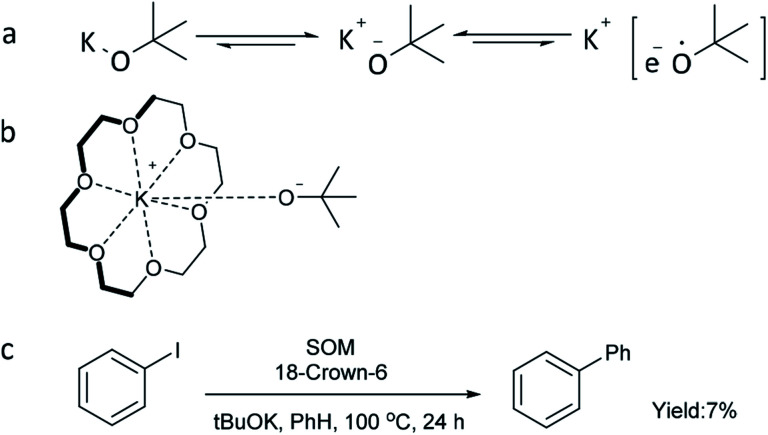
Dissociation of ^*t*^BuOK and attempt to enhance the reducing power of ^*t*^BuOK. ^a^Reaction conditions for (c) 1a (0.4 mmol), SOM (10 mol%), ^*t*^BuOK (1.2 mmol), benzene (4 mL), 18-crown-6 (4 equiv.), 100 °C, 24 h, GC yield.

According to the literature,^44,62^ strong alkalis such as ^*t*^BuOK can be in dynamic equilibrium between the charge-separated and essentially covalent species, especially in an organic phase (Scheme 3a). Moreover, a loosely bound electron (only in the charge-separated form) can be transferred much more easily to substrates to start the reaction. Considering that the reducing ability of metal alkoxide anions can be enhanced by charge separation between the alkoxy anion and metal cation, an experiment using 18-crown-6 to capture potassium cation was conducted (Scheme 3b and c). However, only 7% yield of biphenyl was obtained, indicating that capture of potassium cation alone is not sufficient to improve the reaction efficiency and that the potassium cation itself plays an important role in the transformation. This is also consistent with the fact that ^*t*^BuONa and ^*t*^BuOLi could not catalyze the reaction (Fig. 1a).

**Scheme 4:**
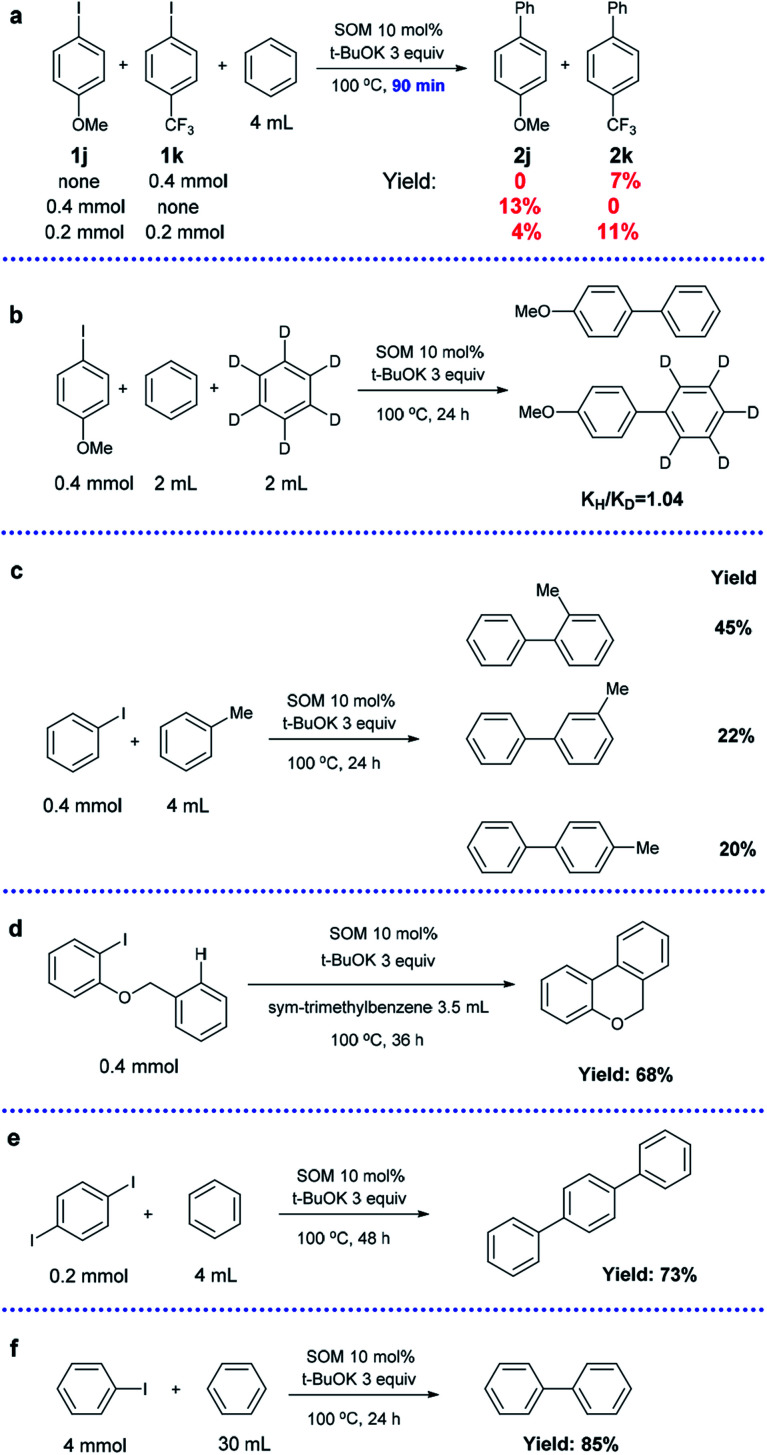
Competition reactions between two different aryl iodides (a), kinetic isotope experiment (b), arylation using toluene (c), intramolecular cross-coupling reaction (d), arylation of paradiiodobenzene (e), and scale-up experiment (f).

Competition experiments using substrates with an electron-donating substituent group (1j) and electron-withdrawing substituent group (1k) were conducted (Scheme 4a). In the control groups (1j or 1k only), the yield of 2j was almost twice that of 2k after 90 min under optimized conditions (13% *vs.* 7%). However, a competition reaction with the coexistence of 1j and 1k in equimolar amounts revealed that electron-deficient 1k possessed much higher reactivity compared to electron-sufficient 1j (4% *vs.* 11%). This result is consistent with the SET mechanism, in which an electron-withdrawing substituent group is beneficial to the stability of intermediates.^35,62^

A kinetic isotope experiment was carried out and a low *k*_H_/*k*_D_ value of 1.04 of the products was observed, implying that the rate-determining step was not the cleavage of aromatic C–H bonds (Scheme 4b). The corresponding NMR spectrum is displayed in the ESI (Fig. S1†).

Next, we changed benzene to toluene (the other conditions were the same); a mixture of *o*-, *m*- and *p*-methylbiphenyls was obtained with a total yield of 87% and a product molar ratio of 2 : 1 : 0.9 (Scheme 4c). The radical mechanism was confirmed by the high *ortho* selectivity of the substituted arenes.^63^

An intramolecular version of the reaction was investigated by conducting a cyclization reaction using 1-(benzyloxy)-2-iodobenzene as the substrate (Scheme 4d). *sym*-Trimethylbenzene was selected as a suitable solvent due to its relatively low reactivity. The exclusive product 6*H*-benzo[*c*]-chromene was obtained in 68% isolated yield, indicating potential application in the preparation of fused ring systems.

In addition, *para*-diiodobenzene could be transformed into terphenyl after a longer time (73%), showing high bi-functionalization efficiency (Scheme 4e).

In order to determine the practical viability of the approach, an experiment was carried out at 10 times larger scale, and a yield of 85% was obtained; this indicates that our catalytic system can find valuable applications in industrial production (Scheme 4f).

^*t*^BuOK generally acts as an medium for electron transfer and was reported to deliver an electron to substrate aryl iodides in such transformations in the first step.^44,64^ In addition, consistent with previous literature, the hydrogen bonds between SOM and ^*t*^BuOK achieved the activation of the catalytic system.^49^ A set of FT-IR experiments were conducted, and the broad peak at around 3420 cm^−1^ (active hydrogens) was dramatically narrowed in the spectrum of a physical mixture of SOM and ^*t*^BuOK compared to that of SOM only (for the FT-IR spectra, see ESI, Fig. S2†). This implies that the active hydrogens on SOM are captured by ^*t*^BuOK through hydrogen bonds^49^ and that this special combination accomplished the activation of the catalytic system.

**Scheme 5:**
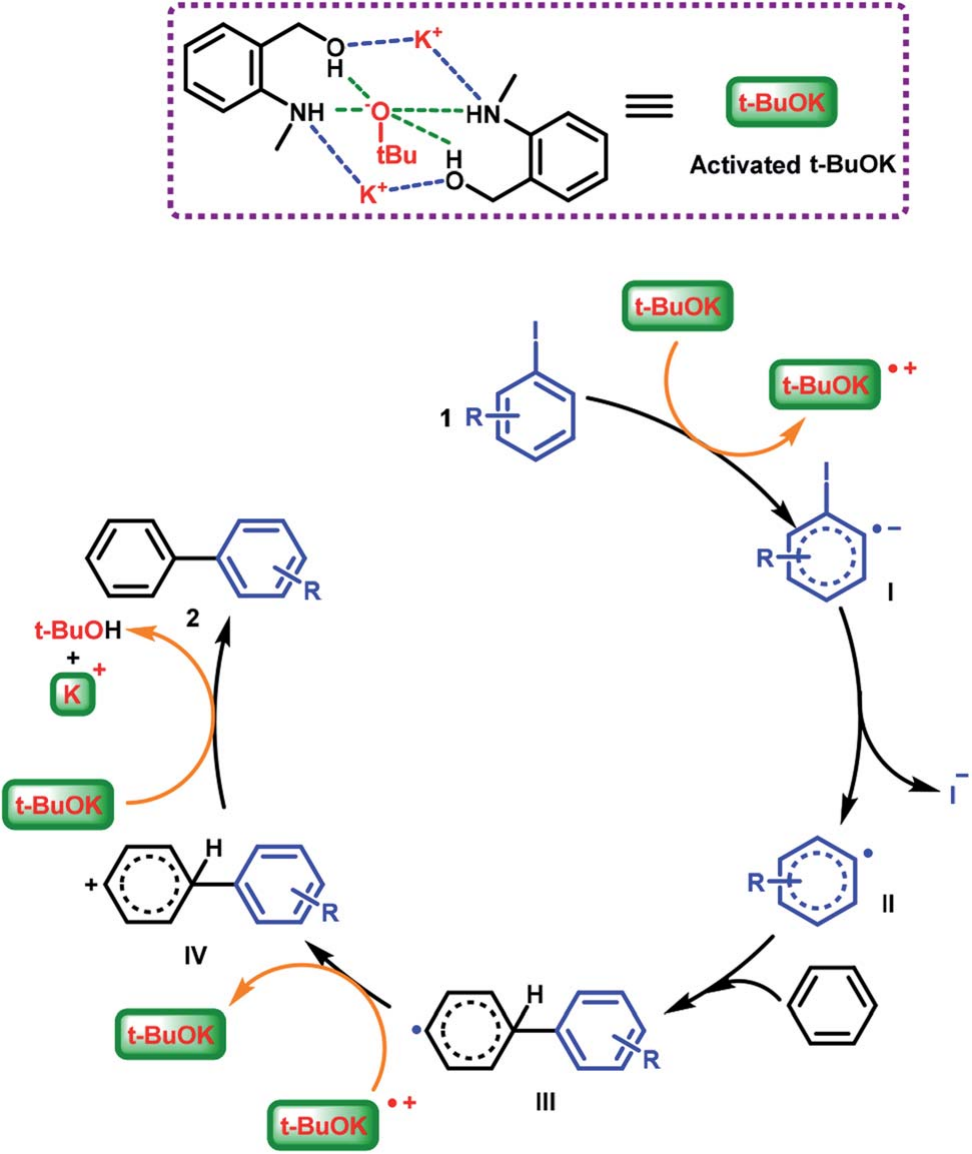
Proposed mechanism for the (2-(methylamino)phenyl)methanol/^*t*^BuOK-catalyzed C–H arylation.

Based on the above explorations and related reports,^49,64^ the interactions (hydrogen bonding and coulombic attraction) of the potassium cation and the *tert*-butoxide anion with (2-(methylamino)phenyl)methanol completed the activation of the catalytic species and further promoted the C–C bond formation reaction. A catalytic mechanism involving radicals was proposed (Scheme 5). Activated ^*t*^BuOK delivered a single electron to aryl iodide 1, affording the intermediate I (aryl radical anion). Then departure of an iodide anion generated aryl radical II, which then combined benzene to form a biaryl radical III. The previously formed radical cation of ^*t*^BuOK oxidized intermediate III and produced the biaryl cation IV. Finally, a *tert*-butoxide anion captured a proton from IV, affording biaryl product 2.

## 3. Conclusions

A convenient catalytic system based on N- and O-containing small organic molecules was established. Arylated products were obtained from aryl iodides in good to excellent yields. A series of mechanism experiments were conducted, and a possible mechanism was proposed. Scale-up experiments confirmed the feasibility of industrial application of the catalytic system. In addition, explorations of other interesting applications of tailored molecules are ongoing.

## 4. Experimental

### 4.1 Materials

All reagents and solvents were purchased from commercial sources (Innochem Science & Technology Co. Ltd, Sigma-Aldrich LLC., J&K Scientific Ltd, Shanghai Xianding Biological Technology Co. Ltd, Amatek Scientific Co. Ltd, Zhengzhou Yi Chemical Products Co. Ltd, and Suzhou Medinoah Pharmaceutical Technology Co. Ltd, Wuxi Kehua biotechnology Co. Ltd, Camus (Suzhou) biotechnology Co. Ltd, and Shanghai Haohong biomedical technology Co. Ltd). Benzene was dried and distilled from sodium/benzophenone under argon atmosphere immediately prior to use.

### 4.2 Instrumentation

Liquid ^1^H and ^13^C NMR spectra were recorded on a Bruker 400 spectrometer. GC analysis was performed on an Agilent 4890D with a FID detector and a nonpolar capillary column (DB-5) (30 m × 0.25 mm × 0.25 μm). The column oven was temperature-programmed with a 2 min initial hold at 323 K followed by a temperature increase to 538 K at a rate of 10 K min^−1^; it was then maintained at 538 K for 10 min. High purity nitrogen was used as a carrier gas. FT-IR spectra of the samples were collected on a TENSOR 27 FTIR at a resolution of 2 cm^−1^.

### 4.3 General procedure for arylation reactions

In a typical procedure, iodobenzene (0.4 mmol), initiator (10 mol%), ^*t*^BuOK (1.2 mmol), and benzene (4 mL) were successively placed in a 15 mL glass tube with a cap; the tube was then sealed tightly and heated to 100 °C. After reacting for 24 h, the reaction mixture was cooled to room temperature. To determine the GC yield, dodecane (internal standard, 0.05 g) and CH_2_Cl_2_ (8 mL) were added, and the reaction solution was then stirred vigorously and centrifuged. The upper liquid was analyzed by GC. To determine the isolated yield, the excess benzene was distilled under vacuum. The residues were quenched with water (4 mL) and extracted with ethyl acetate (3 × 8 mL). The combined organic phase was washed with brine (10 mL), dried over anhydrous Na_2_SO_4_, filtered and concentrated under vacuum. The crude product was purified by column chromatography using petroleum ether/ethyl acetate as the eluent.

### 4.4 Procedure for competition reactions between two different aryl iodides (a), kinetic isotope experiment (b), arylation using toluene (c), intramolecular cross-coupling reaction (d), arylation of paradiiodobenzene (e), and scale-up experiment (f)

#### Competition reaction between two different aryl iodides (a)

Specific amounts of 1-iodo-4-methoxybenzene (1j) and 1-iodo-4-(trifluoromethyl)benzene (1k), SOM (10 mol%), ^*t*^BuOK (1.2 mmol), and benzene (4 mL) were successively placed in a 15 mL glass tube with a cap; the tube was sealed tightly and then heated to 100 °C. After reacting for 90 min, the reaction mixture was cooled to room temperature. Dodecane (internal standard, 0.05 g) and CH_2_Cl_2_ (8 mL) were added, and the reaction solution was then stirred vigorously and centrifuged. The upper liquid was analyzed by GC to determine the yield.

#### Kinetic isotope experiment (b)

A 15 mL glass tube with a cap was charged with 1-iodo-4-methoxybenzene (0.4 mmol), SOM (10 mol%), ^*t*^BuOK (1.2 mmol), benzene-H_6_ (2 mL) and benzene-D_6_ (2 mL) at room temperature. The tube was then sealed and the resulting mixture was stirred at 100 °C for 24 h. After cooling to room temperature, the reaction mixture was quenched (water 4 mL) and extracted with ethyl acetate (8 mL × 3). The organic layers were combined, dried over anhydrous Na_2_SO_4_, concentrated under reduced pressure, and then purified by silica gel chromatography to yield the desired product (using petroleum ether/ethyl acetate as the eluent). The product distribution (*k*_H_/*k*_D_ = 1.04) was analyzed by ^1^H NMR spectroscopy. The corresponding NMR spectrum is shown in the ESI (Fig. S1†).

#### Arylation using toluene (c)

Iodobenzene (0.4 mmol), SOM (10 mol%), ^*t*^BuOK (1.2 mmol), and toluene (4 mL) were successively placed into a 15 mL glass tube with a cap; the tube was sealed tightly and then heated to 100 °C. After reacting for 24 h, the reaction mixture was cooled to room temperature. To determine the GC yield, dodecane (internal standard, 0.05 g) and CH_2_Cl_2_ (8 mL) was added, and the reaction solution was then stirred vigorously and centrifuged. The upper liquid was analyzed by GC.

#### Intramolecular cross-coupling reaction (d)

1-(Benzyloxy)-2-iodobenzene (0.4 mmol), SOM (10 mol%), ^*t*^BuOK (1.2 mmol), and *sym*-trimethylbenzene (3.5 mL) were successively placed in a 15 mL glass tube with a cap; the tube was sealed tightly and then heated to 100 °C. After reacting for 24 h, the reaction mixture was cooled to room temperature. The yield was determined by column chromatography using petroleum ether/ethyl acetate as the eluent.

#### Arylation of paradiiodobenzene (e)

1,4-Diiodobenzene (0.2 mmol), SOM (10 mol%), ^*t*^BuOK (1.2 mmol), and benzene (4 mL) were successively placed in a 15 mL glass tube with a cap; the tube was sealed tightly and then heated to 100 °C. After reacting for 24 h, the reaction mixture was cooled to room temperature. The yield was determined by column chromatography using petroleum ether/ethyl acetate as the eluent.

#### Scale-up experiment (f)

Iodobenzene (4 mmol), SOM (10 mol%), ^*t*^BuOK (12 mmol), and benzene (30 mL) were successively placed in a 60 mL stainless steel reaction still, which was sealed tightly and then heated to 100 °C. After reacting for 24 h, the reaction mixture was cooled to room temperature. The yield was determined by column chromatography using petroleum ether/ethyl acetate as the eluent.

### 4.5 NMR characterization of products

Spectroscopic data of all products are exhibited as follows. All the products gave satisfactory spectroscopic values and were analogous to spectroscopic data in previous literature reports.

#### 1,1′-Biphenyl (2a)^65^

^1^H NMR (400 MHz, chloroform-d) *δ* 7.61–7.54 (m, 4H), 7.41 (dd, *J* = 8.3, 6.9 Hz, 4H), 7.36–7.27 (m, 2H); ^13^C NMR (101 MHz, chloroform-d) *δ* 141.2, 128.7, 127.2, 127.1.

#### 2-Methyl-1,1′-biphenyl (2b)^65^

^1^H NMR (400 MHz, chloroform-d) *δ* 7.42–7.36 (m, 2H), 7.31 (td, *J* = 6.4, 5.7, 2.5 Hz, 3H), 7.27–7.20 (m, 4H), 2.26 (s, 3H); ^13^C NMR (101 MHz, chloroform-d) *δ* 141.9, 135.3, 130.3, 129.8, 129.1, 128.0, 127.2, 126.7, 125.7, 20.4.

#### 3-Methyl-1,1′-biphenyl (2c)^65^

^1^H NMR (400 MHz, chloroform-d) *δ* 7.59–7.55 (m, 2H), 7.40 (dt, *J* = 9.7, 7.7 Hz, 4H), 7.32 (td, *J* = 7.5, 1.4 Hz, 2H), 7.15 (d, *J* = 7.4 Hz, 1H), 2.41 (s, 3H); ^13^C NMR (101 MHz, chloroform-d) *δ* 141.3, 141.2, 138.3, 128.7, 128.6, 128.0, 127.9, 127.2, 127.1, 124.3, 21.5.

#### 4-Methyl-1,1′-biphenyl (2d)^65^

^1^H NMR (400 MHz, chloroform-d) *δ* 7.56 (dd, *J* = 8.2, 1.4 Hz, 2H), 7.51–7.44 (m, 2H), 7.40 (dd, *J* = 8.3, 6.9 Hz, 2H), 7.33–7.26 (m, 1H), 7.22 (d, *J* = 7.8 Hz, 2H), 2.37 (s, 3H); ^13^C NMR (101 MHz, chloroform-d) *δ* 141.1, 138.3, 137.0, 129.5, 128.7, 127.0, 126.9, 21.1.

#### 2-Fluoro-1,1′-biphenyl (2e)^45^

^1^H NMR (400 MHz, chloroform-d) *δ* 7.54 (dd, *J* = 7.2, 1.7 Hz, 2H), 7.41 (dt, *J* = 7.6, 5.7 Hz, 3H), 7.38–7.32 (m, 1H), 7.28 (tdd, *J* = 7.3, 4.9, 1.8 Hz, 1H), 7.20–7.09 (m, 2H); ^13^C NMR (101 MHz, chloroform-d) *δ* 161.0, 158.5, 135.8, 130.8, 130.7, 129.0, 129.0, 128.9, 128.8, 128.4, 127.6, 124.3, 124.3, 116.2, 116.0.

#### 3-Fluoro-1,1′-biphenyl (2f)^45^

^1^H NMR (400 MHz, chloroform-d) *δ* 7.57–7.52 (m, 2H), 7.45–7.40 (m, 2H), 7.35 (qd, *J* = 5.5, 3.8 Hz, 3H), 7.27 (dt, *J* = 9.7, 1.9 Hz, 1H), 7.01 (ddt, *J* = 11.3, 6.1, 2.5 Hz, 1H); ^13^C NMR (101 MHz, chloroform-d) *δ* 164.4, 161.9, 143.5, 143.4, 139.9, 139.9, 130.2, 130.1, 128.8, 127.8, 127.1, 122.7, 122.7, 114.1, 114.1, 113.9, 113.9.

#### 4-Fluoro-1,1′-biphenyl (2g)^65^

^1^H NMR (400 MHz, chloroform-d) *δ* 7.53 (dd, *J* = 6.8, 2.4 Hz, 4H), 7.42 (t, *J* = 7.6 Hz, 2H), 7.33 (t, *J* = 7.3 Hz, 1H), 7.11 (t, *J* = 8.5 Hz, 2H); ^13^C NMR (101 MHz, chloroform-d) *δ* 163.7, 161.2, 140.2, 137.3, 128.8, 128.7, 128.6, 127.2, 127.0, 115.7, 115.5.

#### 2-Methoxy-1,1′-biphenyl (2h)^66^

^1^H NMR (400 MHz, chloroform-d) *δ* 7.72–7.66 (m, 2H), 7.55 (t, *J* = 7.8 Hz, 2H), 7.50–7.40 (m, 3H), 7.17 (td, *J* = 7.5, 1.1 Hz, 1H), 7.10 (dd, *J* = 8.3, 1.1 Hz, 1H), 3.91 (s, 3H); ^13^C NMR (101 MHz, chloroform-d) *δ* 156.4, 138.5, 130.8, 130.6, 129.5, 128.5, 127.9, 126.8, 120.8, 111.2, 77.3, 77.0, 76.8, 55.4.

#### 3-Methoxy-1,1′-biphenyl (2i)^5^

^1^H NMR (400 MHz, chloroform-d) *δ* 7.76–7.64 (m, 2H), 7.53 (t, *J* = 7.5 Hz, 2H), 7.49–7.39 (m, 2H), 7.31–7.23 (m, 2H), 7.00 (dd, *J* = 8.3, 2.5 Hz, 1H), 3.94 (s, 3H); ^13^C NMR (101 MHz, chloroform-d) *δ* 159.9, 142.7, 141.0, 129.7, 128.7, 127.4, 127.1, 119.6, 112.8, 112.6, 77.4, 77.0, 76.6, 55.2.

#### 4-Methoxy-1,1′-biphenyl (2j)^65^

^1^H NMR (400 MHz, chloroform-d) *δ* 7.53 (tt, *J* = 9.6, 2.3 Hz, 4H), 7.40 (t, *J* = 7.7 Hz, 2H), 7.33–7.26 (m, 1H), 7.00–6.93 (m, 2H), 3.83 (s, 3H); ^13^C NMR (101 MHz, chloroform-d) *δ* 159.1, 140.8, 133.8, 128.7, 128.1, 126.7, 126.6, 114.2, 55.3.

#### 4-(Trifluoromethyl)-1,1′-biphenyl (2k)^34^

^1^H NMR (400 MHz, chloroform-d) *δ* 7.68 (s, 4H), 7.61–7.56 (m, 2H), 7.49–7.43 (m, 2H), 7.43–7.36 (m, 1H); ^13^C NMR (101 MHz, chloroform-d) *δ* 144.7, 139.8, 129.0, 128.2, 127.4, 127.3, 125.7, 125.7.

#### [1,1′-Biphenyl]-4-carbonitrile (2l)^65^

^1^H NMR (400 MHz, chloroform-d) *δ* 7.90–7.84 (m, 2H), 7.77–7.72 (m, 2H), 7.62–7.56 (m, 2H), 7.49–7.42 (m, 2H), 7.39–7.32 (m, 1H); ^13^C NMR (101 MHz, chloroform-d) *δ* 141.0, 140.6, 132.6, 129.0, 127.7, 127.6, 127.1, 118.5, 110.3.

#### *N*,*N*-Dimethyl-[1,1′-biphenyl]-4-amine (2m)^67^

^1^H NMR (400 MHz, chloroform-d) *δ* 7.61–7.55 (m, 2H), 7.48–7.38 (m, 4H), 7.36–7.29 (m, 1H), 6.84–6.78 (m, 2H), 3.02 (s, 6H); ^13^C NMR (101 MHz, chloroform-d) *δ* 151.0, 140.8, 132.2, 128.9, 127.7, 127.3, 127.1, 112.5, 40.3.

#### 4-(Trifluoromethoxy)-1,1′-biphenyl (2n)^68^

^1^H NMR (400 MHz, chloroform-d) *δ* 7.58 (t, *J* = 1.6 Hz, 1H), 7.58–7.50 (m, 3H), 7.46–7.38 (m, 4H), 7.38–7.30 (m, 1H); ^13^C NMR (101 MHz, chloroform-d) *δ* 150.2, 150.1, 140.6, 134.1, 129.4, 128.7, 127.8, 127.3, 122.0, 121.9, 121.6, 119.5.

#### 4-Isopropyl-1,1′-biphenyl (2o)^68^

^1^H NMR (400 MHz, chloroform-d) *δ* 7.61–7.52 (m, 4H), 7.46–7.39 (m, 2H), 7.36–7.29 (m, 3H), 2.93 (m, 1H), 1.28 (d, *J* = 6.8 Hz, 6H); ^13^C NMR (101 MHz, chloroform-d) *δ* 146.5, 140.6, 138.8, 128.9, 127.7, 127.3, 127.1, 126.6, 33.9, 23.9.

#### 4-(*tert*-Butyl)-1,1′-biphenyl (2p)^65^

^1^H NMR (400 MHz, chloroform-d) *δ* 7.60–7.56 (m, 2H), 7.55–7.50 (m, 2H), 7.48–7.44 (m, 2H), 7.41 (dd, *J* = 8.4, 7.0 Hz, 2H), 7.36–7.27 (m, 1H), 1.36 (s, 9H); ^13^C NMR (101 MHz, chloroform-d) *δ* 150.2, 141.0, 138.3, 128.7, 127.0, 126.9, 126.8, 125.7, 34.5, 31.4.

#### 1-([1,1′-Biphenyl]-4-yl)ethan-1-one (2q)^66^

^1^H NMR (400 MHz, chloroform-d) *δ* 8.14–8.02 (m, 2H), 7.77–7.64 (m, 4H), 7.57–7.21 (m, 3H), 2.66 (t, *J* = 3.5 Hz, 3H); ^13^C NMR (101 MHz, chloroform-d) *δ* 129.1, 129.0, 128.9, 128.2, 127.3, 127.2, 127.1, 26.6.

#### 1,1′:4′,1′′-Terphenyl (2r)^65^

^1^H NMR (400 MHz, chloroform-d) *δ* 7.71–7.60 (m, 8H), 7.50–7.41 (m, 4H), 7.39–7.32 (m, 2H); ^13^C NMR (101 MHz, chloroform-d) *δ* 128.9, 128.1, 127.1, 26.5.

#### 3,5-Dimethyl-1,1′-biphenyl (2s)^64^

^1^H NMR (400 MHz, chloroform-d) *δ* 7.62–7.55 (m, 2H), 7.43–7.34 (m, 3H), 7.19 (d, *J* = 1.5 Hz, 2H), 6.82 (m, 1H), 2.27 (s, 6H); ^13^C NMR (101 MHz, chloroform-d) *δ* 138.7, 137.3, 137.2, 129.3, 128.9, 128.3, 126.6, 126.3, 21.5.

#### 2-Phenylnaphthalene (2t)^65^

^1^H NMR (400 MHz, chloroform-d) *δ* 8.03–7.95 (m, 2H), 7.89 (m, 1H), 7.70 (m, 1H), 7.64–7.58 (m, 2H), 7.56–7.46 (m, 3H), 7.45–7.41 (m, 2H), 7.41–7.33 (m, 1H); ^13^C NMR (101 MHz, chloroform-d) *δ* 140.8, 138.1, 133.7, 133.6, 128.5, 128.3, 128.0, 127.9, 127.8, 127.6, 126.7, 126.3, 126.0, 125.9.

#### 2-Phenylthiophene (2u)^66^

^1^H NMR (400 MHz, chloroform-d) *δ* 7.71–7.69 (m, 2H), 7.44 (d, *J* = 8.2 Hz, 2H), 7.39–7.32 (m, 3H), 7.15 (dd, *J* = 5.5, 3.9 Hz, 1H); ^13^C NMR (101 MHz, chloroform-d) *δ* 128.9, 128.0, 127.5, 126.0, 124.8, 123.1.

#### 3-Phenylthiophene (2v)^64^

^1^H NMR (400 MHz, chloroform-d) *δ* 7.60–7.53 (m, 2H), 7.45 (dd, *J* = 2.8, 1.5 Hz, 1H), 7.43–7.37 (m, 3H), 7.37–7.31 (m, 1H), 7.23 (dd, *J* = 7.5, 1.5 Hz, 1H); ^13^C NMR (101 MHz, chloroform-d) *δ* 142.8, 137.1, 128.8, 127.5, 127.4, 125.9, 125.4, 122.7.

#### 2-Phenylpyridine (2w)^66^

^1^H NMR (400 MHz, chloroform-d) *δ* 8.72 (dt, *J* = 4.6, 1.4 Hz, 1H), 8.06–8.02 (m, 2H), 7.73–7.69 (m, 2H), 7.50 (dd, *J* = 8.5, 6.8 Hz, 2H), 7.46–7.41 (m, 1H), 7.22–7.17 (m, 1H); ^13^C NMR (101 MHz, chloroform-d) *δ* 157.2, 149.5, 139.2, 136.5, 128.8, 128.6, 126.7, 121.9, 120.3.

#### 3-Phenylpyridine (2x)^68^

^1^H NMR (400 MHz, chloroform-d) *δ* 8.88–8.81 (m, 1H), 8.57 (dd, *J* = 4.9, 1.6 Hz, 1H), 7.81 (dt, *J* = 7.9, 2.0 Hz, 1H), 7.56–7.52 (m, 2H), 7.44 (dd, *J* = 8.5, 6.9 Hz, 2H), 7.39–7.35 (m, 1H), 7.30 (m, 1H); ^13^C NMR (101 MHz, chloroform-d) *δ* 148.2, 148.0, 137.5, 136.3, 134.0, 128.8, 127.8, 126.8, 123.2.

#### 4-Phenylpyridine (2y)^5^

^1^H NMR (400 MHz, DMSO-d_6_) *δ* 8.68–8.63 (m, 2H), 7.91–7.76 (m, 2H), 7.71–7.68 (m, 2H), 7.69–7.37 (m, 3H); ^13^C NMR (101 MHz, DMSO-d_6_) *δ* 150.0, 129.3, 129.2, 126.8, 121.3, 39.2.

#### 4-Chloro-2-fluoro-1,1′-biphenyl (2z)

^1^H NMR (400 MHz, chloroform-d) *δ* 7.53–7.43 (m, 5H), 7.39–7.32 (m, 2H), 7.29 (dd, *J* = 8.0, 1.5 Hz, 1H); ^13^C NMR (101 MHz, chloroform-d) *δ* 160.5, 158.5, 137.1, 135.4, 130.2, 128.60, 127.9, 127.7, 126.9, 115.5.

#### 4-Fluoro-3-methoxy-1,1′-biphenyl (2aa)

^1^H NMR (400 MHz, chloroform-d) *δ* 7.62–7.56 (m, 2H), 7.47–7.41 (m, 2H), 7.41–7.36 (m, 1H), 7.33 (ddd, *J* = 7.5, 5.0, 1.5 Hz, 1H), 7.19 (dd, *J* = 8.0, 7.5 Hz, 1H), 7.15 (dd, *J* = 5.0, 1.5 Hz, 1H), 3.93 (s, 3H); ^13^C NMR (101 MHz, chloroform-d) *δ* 155.0, 153.0, 149.1, 140.2, 137.4, 127.7, 123.4, 117.7, 112.3, 56.6.

#### 3-Methoxy-5-(trifluoromethyl)-1,1′-biphenyl (2ab)

^1^H NMR (400 MHz, chloroform-d) *δ* 7.63–7.57 (m, 3H), 7.47–7.40 (m, 2H), 7.40–7.34 (m, 1H), 7.17 (dt, *J* = 17.9, 1.5 Hz, 2H), 3.53 (s, 3H); ^13^C NMR (101 MHz, chloroform-d) *δ* 158.9, 139.2, 137.0, 132.1, 128.7, 127.2, 124.6, 123.9, 122.4, 113.2, 110.3, 55.7.

#### 3,4,5-Trimethoxy-1,1′-biphenyl (2ac)

^1^H NMR (400 MHz, chloroform-d) *δ* 7.62–7.56 (m, 2H), 7.47–7.40 (m, 2H), 7.40–7.34 (m, 1H), 6.78 (s, 2H), 3.84 (d, *J* = 12.6 Hz, 9H); ^13^C NMR (101 MHz, chloroform-d) *δ* 152.9, 141.4, 138.7, 133.8, 128.8, 128.3, 127.1, 106.7, 60.8, 56.2.

#### 2-Methyl-4-phenylpyridine (2ad)

^1^H NMR (400 MHz, chloroform-d) *δ* 8.55 (d, *J* = 7.5 Hz, 1H), 7.62–7.56 (m, 2H), 7.47–7.44 (m, 1H), 7.44–7.35 (m, 3H), 7.35–7.32 (m, 1H), 2.74 (d, *J* = 0.5 Hz, 3H); ^13^C NMR (101 MHz, chloroform-d) *δ* 158.1, 148.9, 142.8, 138.6, 128.9, 127.9, 120.6, 119.8, 24.7.

#### 4-Chloro-2-phenylpyridine (2ae)

^1^H NMR (400 MHz, chloroform-d) *δ* 8.45 (d, *J* = 7.5 Hz, 1H), 8.00–7.94 (m, 2H), 7.78 (d, *J* = 1.5 Hz, 1H), 7.51–7.44 (m, 2H), 7.42–7.35 (m, 1H), 7.24 (dd, *J* = 7.5, 1.5 Hz, 1H); ^13^C NMR (101 MHz, chloroform-d) *δ* 156.7, 148.2, 139.6, 135.2, 129.8, 128.7, 127.4, 122.4, 122.2.

#### 2-Fluoro-6-phenylpyridine (2af)

^1^H NMR (400 MHz, chloroform-d) *δ* 8.00–7.94 (m, 2H), 7.66 (dd, *J* = 7.5, 1.5 Hz, 1H), 7.53 (m, 1H), 7.50–7.44 (m, 2H), 7.42–7.35 (m, 1H), 6.68 (m, 1H); ^13^C NMR (101 MHz, chloroform-d) *δ* 163.1, 161.1, 154.8, 141.1, 135.2, 129.8, 127.5, 118.6, 108.4.

#### 3-Methoxy-5-phenylpyridine (2ag)

^1^H NMR (400 MHz, chloroform-d) *δ* 8.51 (dd, *J* = 1.5, 0.4 Hz, 1H), 8.14 (dd, *J* = 1.5, 0.4 Hz, 1H), 7.57–7.51 (m, 2H), 7.48 (t, *J* = 1.5 Hz, 1H), 7.46–7.39 (m, 2H), 7.39–7.34 (m, 1H), 3.95 (s, 3H); ^13^C NMR (101 MHz, chloroform-d) *δ* 155.5, 143.6, 135.5, 135.3, 134.2, 129.0, 127.9, 127.8, 113.3, 55.8.

#### *6H*-Benzo[*c*]chromene

^1^H NMR (400 MHz, chloroform-d) *δ* 7.93 (dd, *J* = 7.5, 1.5 Hz, 1H), 7.71 (dd, *J* = 7.5, 1.5 Hz, 1H), 7.35 (td, *J* = 7.4, 1.6 Hz, 1H), 7.26–7.18 (m, 2H), 7.13 (m, 2H), 6.59 (dd, *J* = 7.5, 1.5 Hz, 1H), 4.81 (d, *J* = 1.0 Hz, 2H); ^13^C NMR (101 MHz, chloroform-d) *δ* 153.2, 134.1, 128.1, 127.8, 127.7, 127.6, 125.7, 125.3, 123.9, 123.3, 115.3, 69.3.

## Conflicts of interest

There are no conflicts to declare.

## Supplementary Material

RA-010-D0RA01845G-s001
