# Supplementary material for: Small organic molecules with tailored structures: initiators in the transition-metal-free C–H arylation of unactivated arenes
Source: RSC Adv. 2020 Apr 9;10(25):14500–9. doi: 10.1039/d0ra01845g (PMC9051888; doi:10.1039/d0ra01845g)
Supplement: RA-010-D0RA01845G-s001 [file RA-010-D0RA01845G-s001.pdf]

Electronic Supplementary Material (ESI) for RSC Advances.
This journal is © The Royal Society of Chemistry 2020

Electronic Supplementary Information

Small Organic Molecules with Tailored Structures: Initiators in Transition-Metal-Free C–H Arylation of Unactivated Arenes

Zhenghui Liu[a,*], Peng Wang[b,c], Yu Chen[d], Zhenzhong Yan[a], Suqing Chen[a], Wenjun Chen[e], Tiancheng Mu[e,*]

[a] School of Pharmaceutical and Materials Engineering, Taizhou University, Taizhou 318000, Zhejiang, China

[b] Beijing National Laboratory for Molecular Sciences, CAS Research/Education Center for Excellence in Molecular Sciences, Institute of Chemistry, Chinese Academy of Sciences, Beijing 100190, China

[c] Key Laboratory of Green Chemical Media and Reactions, Ministry of Education, School of Chemistry and Chemical Engineering, Henan Normal University, Xinxiang 453007, Henan, China

[d] Department of Chemistry and Material Science, Langfang Normal University, Langfang 065000, Hebei, China

[e] Department of Chemistry, Renmin University of China, Beijing 100872, China

* Corresponding author.

Table of Contents

1. Result of kinetic isotope experiment S3

2. FT-IR spectra of pure SOM and SOM with tBuOK S4

3. Information for GC analysis S5

4. NMR spectra of products S6-S39

1. Result of kinetic isotope experiment

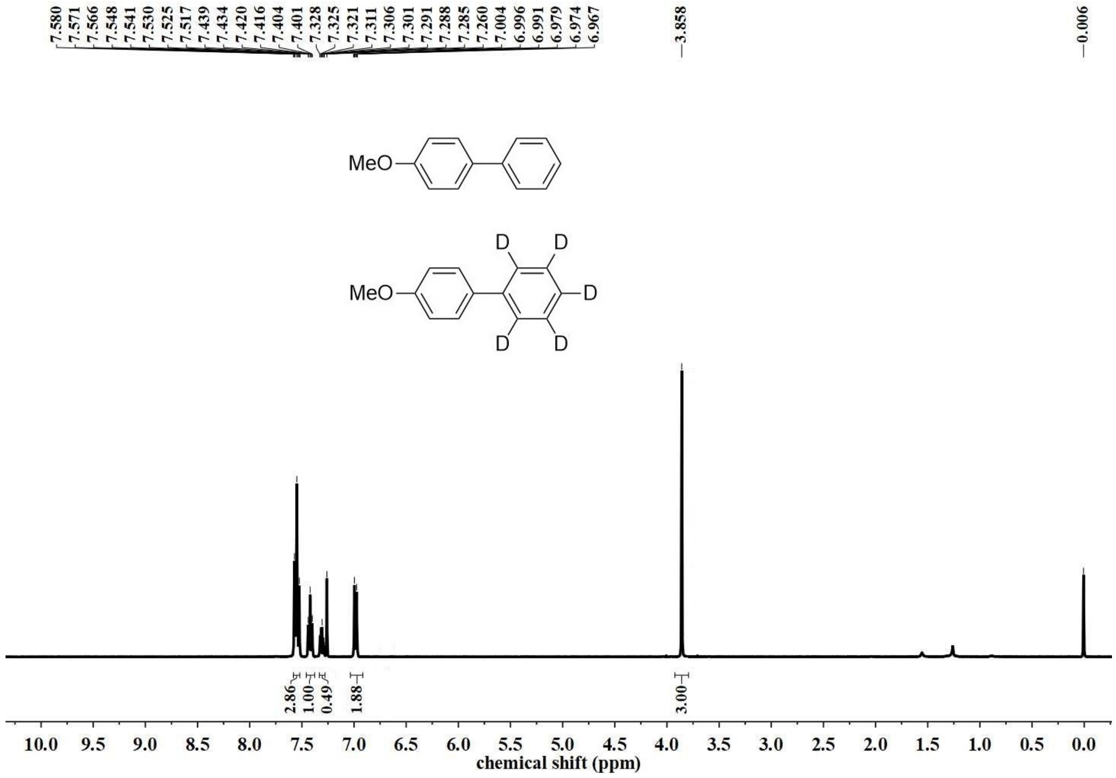

Figure S1 NMR spectrum of kinetic isotope experiment

2. FT-IR spectra of pure SOM and SOM with tBuOK

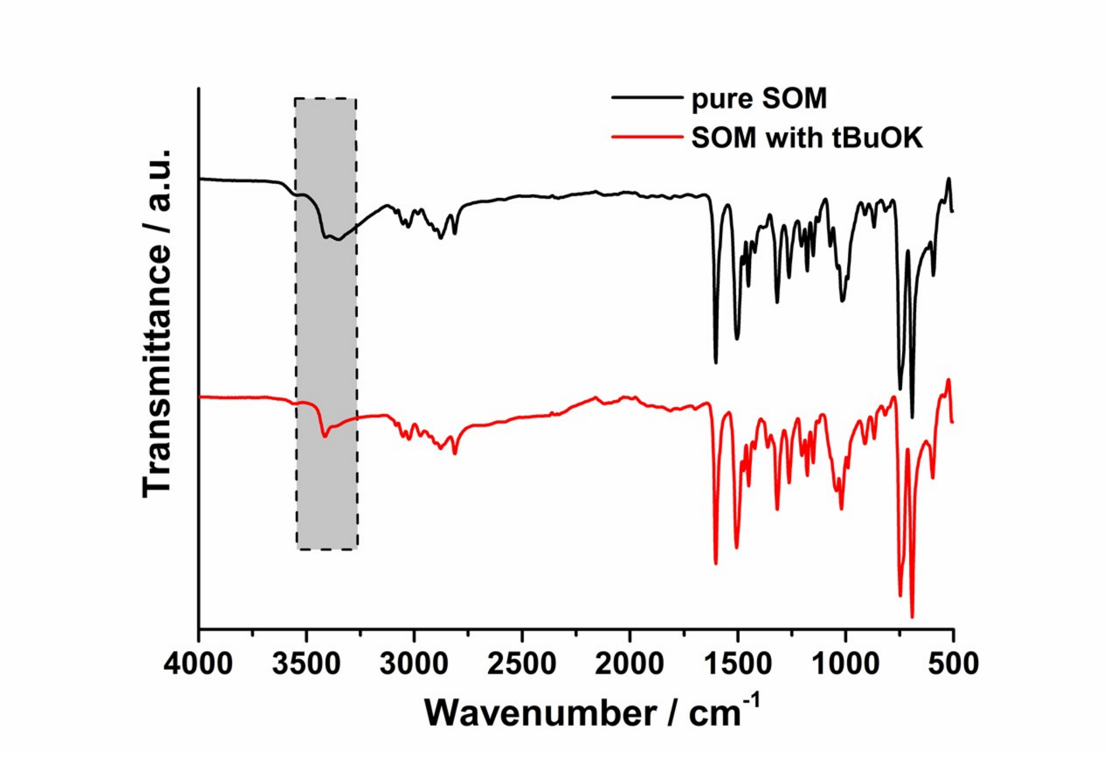

Figure S2 FT-IR spectra of pure SOM and SOM with tBuOK

3. Information for GC analysis

The yields of the product biphenyl of each reaction were determined by GC using dodecane as the internal standard and calculated through the calibartion curves as shown below.

Calibration Curve

Standard Solution	iodobenzene/g	dodecane/g
1	0.0624	0.0488
2	0.1289	0.0502
3	0.2231	0.0491
4	0.3471	0.0509
5	0.4696	0.0502

Standard Solution	biphenyl/g	dodecane/g
1	0.0100	0.0745
2	0.0213	0.0796
3	0.0266	0.0710
4	0.0313	0.1077
5	0.0466	0.0793

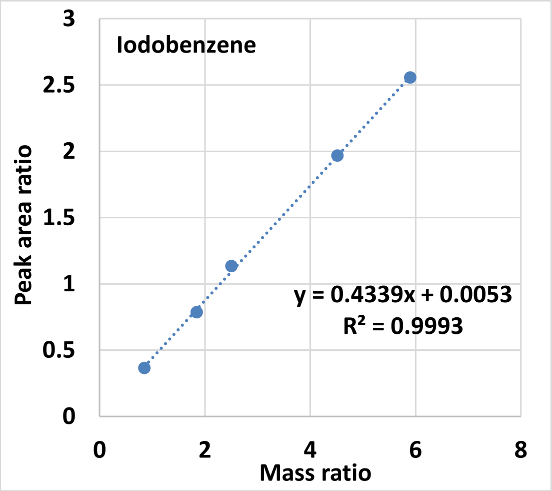

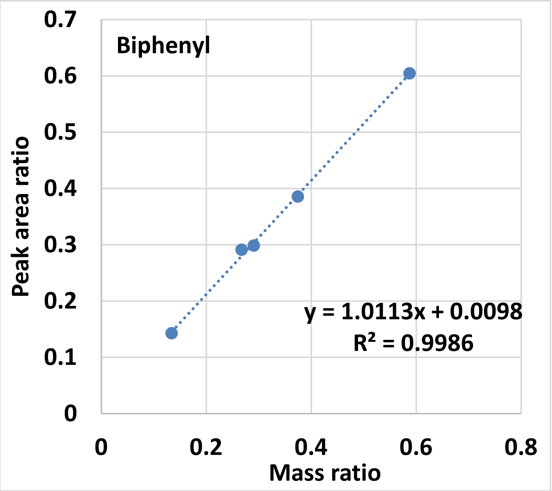

GC spectra

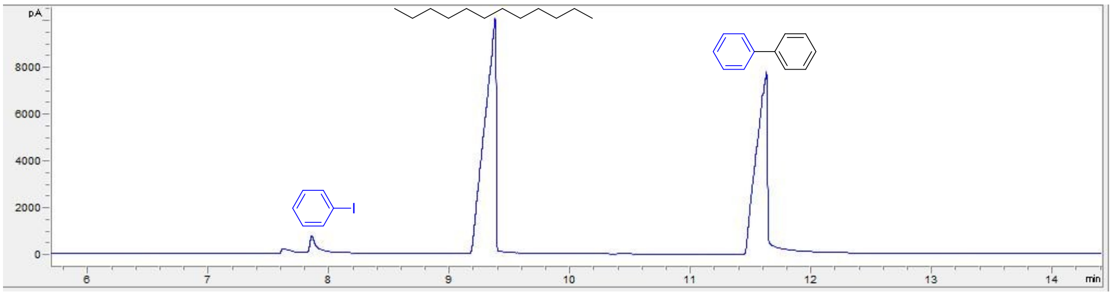

4. NMR spectra of products

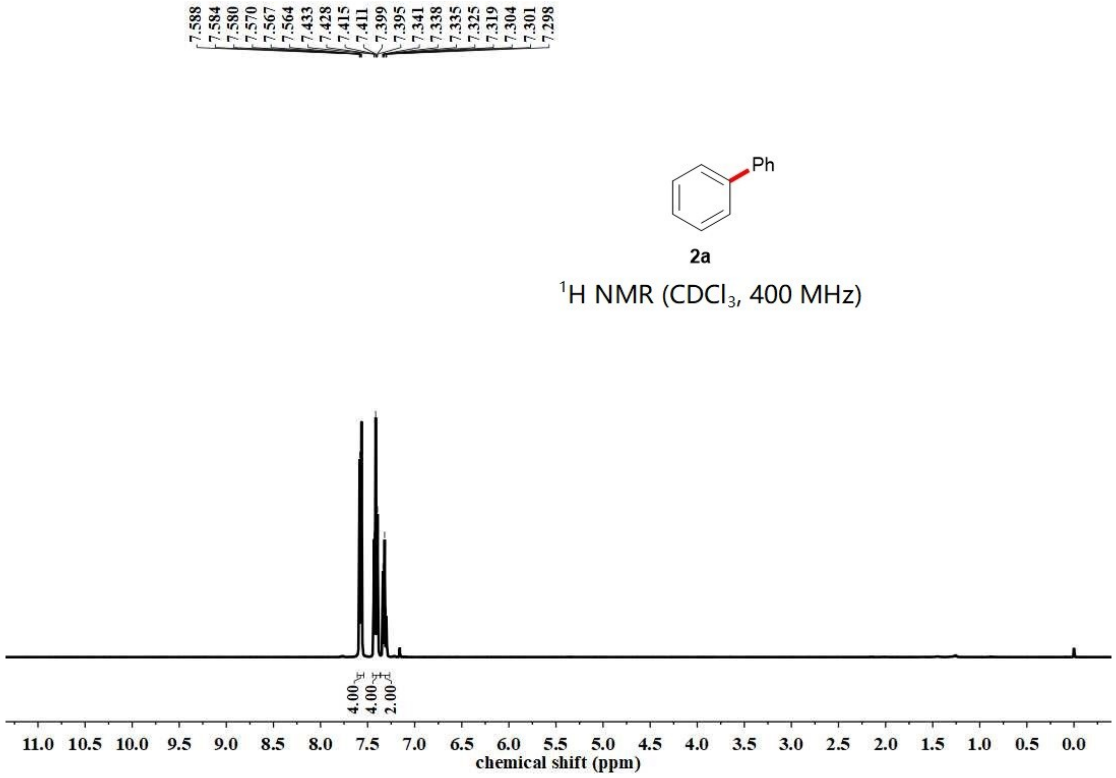

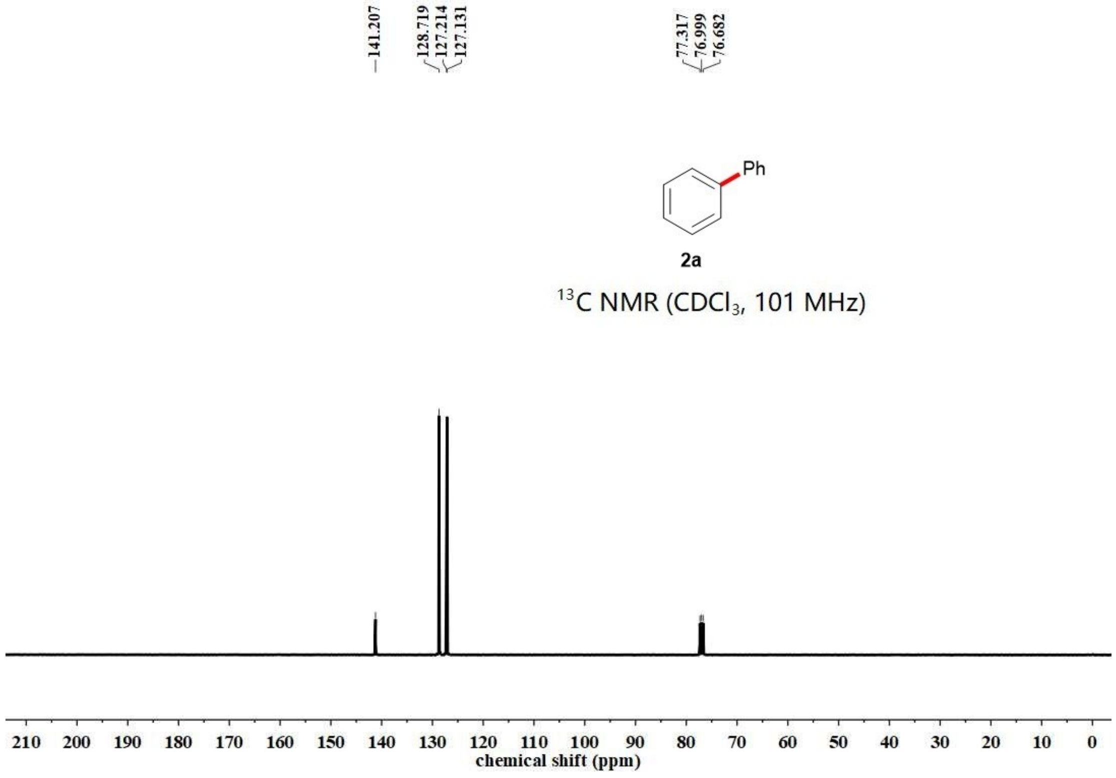

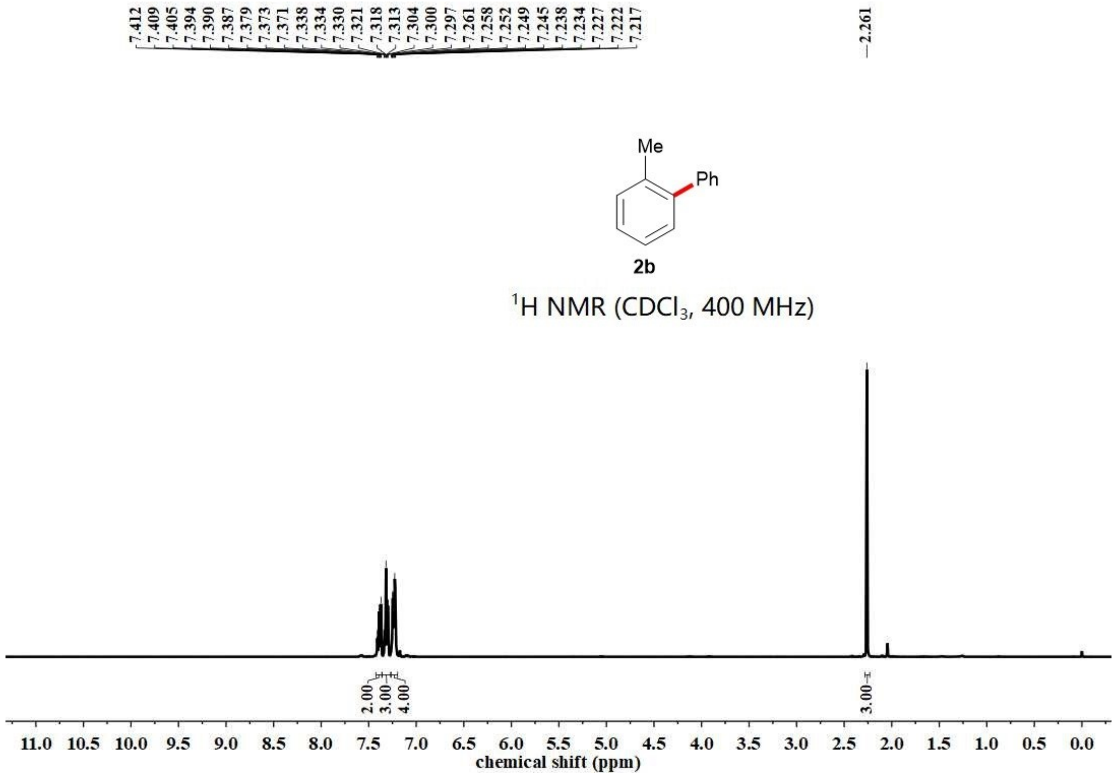
7.412 7.409 7.405 7.394 7.390 7.387 7.379 7.373 7.371 7.338 7.334 7.330 7.321 7.318 7.313 7.304 7.300 7.297 7.261 7.258 7.252 7.249 7.245 7.238 7.234 7.227 7.222 7.217
2.261
Me
Ph
2b
^{1}H NMR (CDCl$_3$, 400 MHz)
2.00
3.00
4.00
3.00
11.0 10.5 10.0 9.5 9.0 8.5 8.0 7.5 7.0 6.5 6.0 5.5 5.0 4.5 4.0 3.5 3.0 2.5 2.0 1.5 1.0 0.5 0.0
chemical shift (ppm)

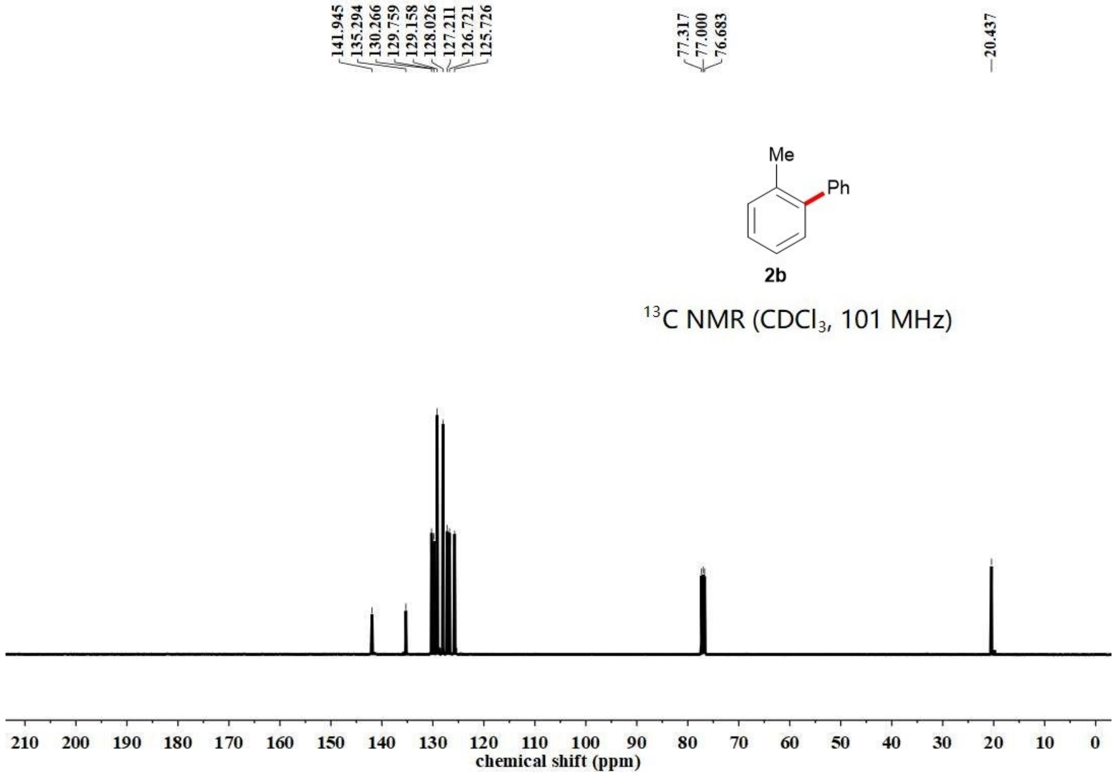
141.945 135.294 130.266 129.759 129.158 128.026 127.211 126.721 125.726
77.317 77.000 76.683
20.437
Me
Ph
2b
^{13}C NMR (CDCl$_3$, 101 MHz)
210 200 190 180 170 160 150 140 130 120 110 100 90 80 70 60 50 40 30 20 10 0
chemical shift (ppm)

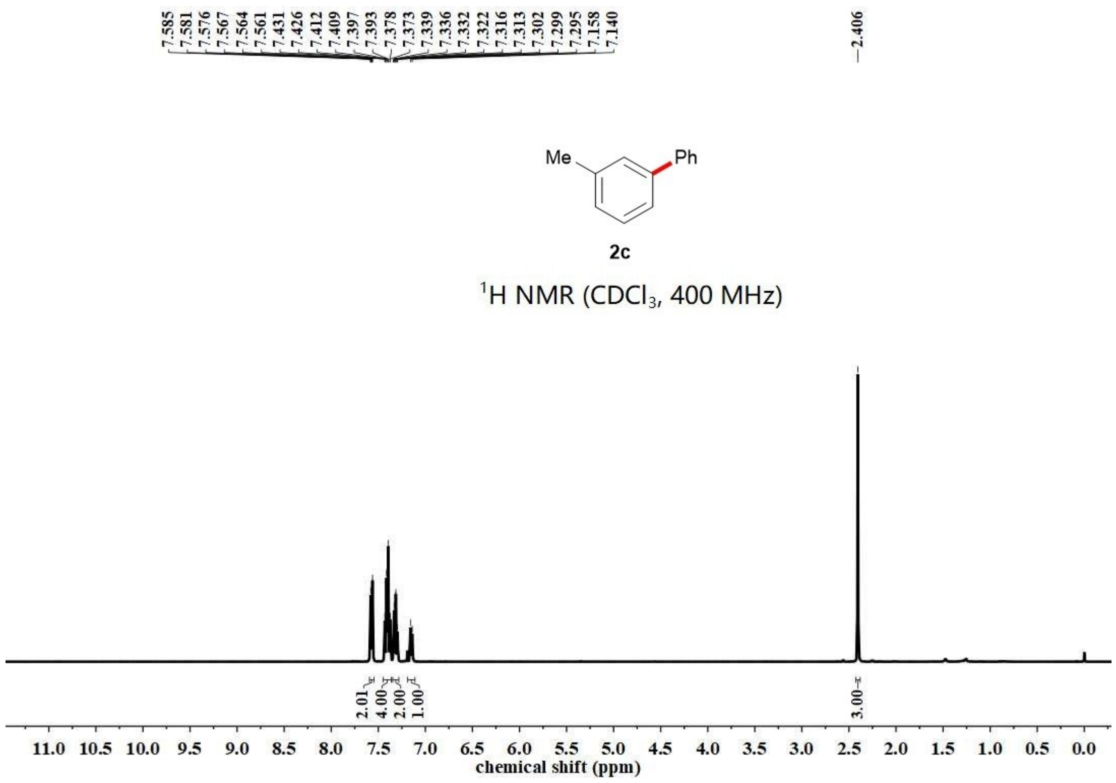
7.585 7.581 7.576 7.567 7.564 7.561 7.431 7.426 7.412 7.409 7.397 7.393 7.378 7.373 7.339 7.336 7.332 7.322 7.316 7.313 7.302 7.299 7.295 7.158 7.140
2.406
Me
Ph
2c
1H NMR (CDCl3, 400 MHz)
2.01 4.00 2.00 1.00
3.00
chemical shift (ppm)

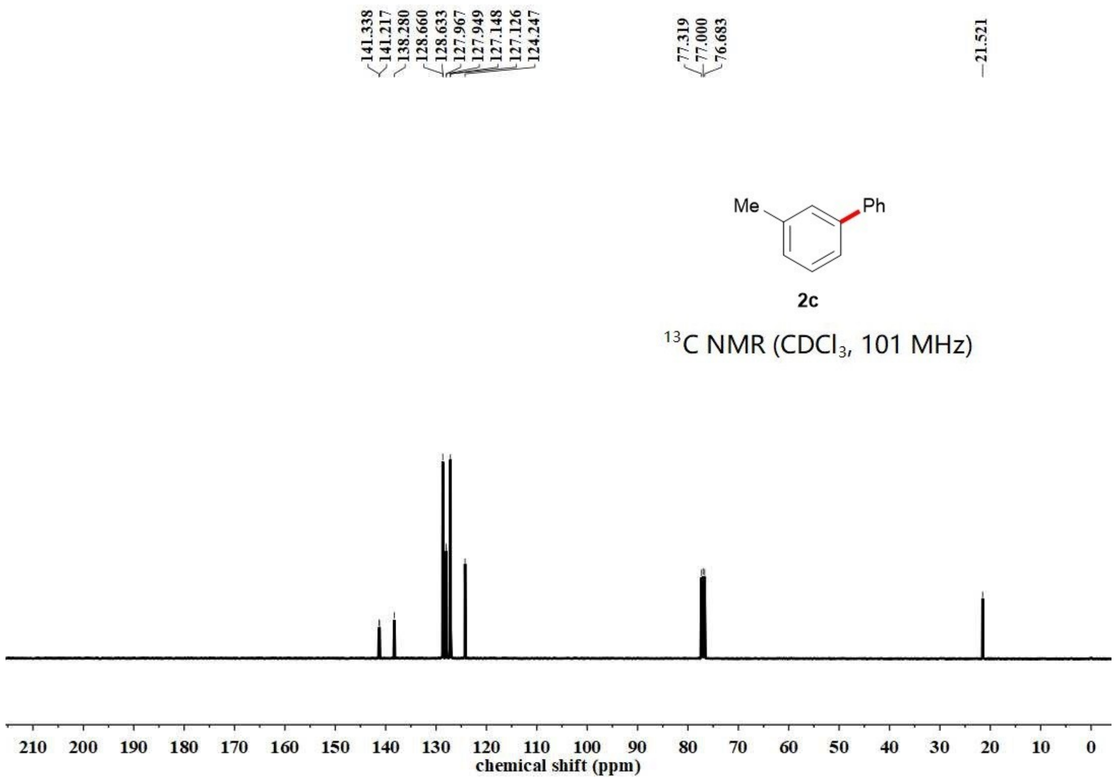
141.338 141.217 138.280 128.660 128.633 127.967 127.949 127.148 127.126 124.247
77.319 77.000 76.683
21.521
Me
Ph
2c
13C NMR (CDCl3, 101 MHz)
chemical shift (ppm)

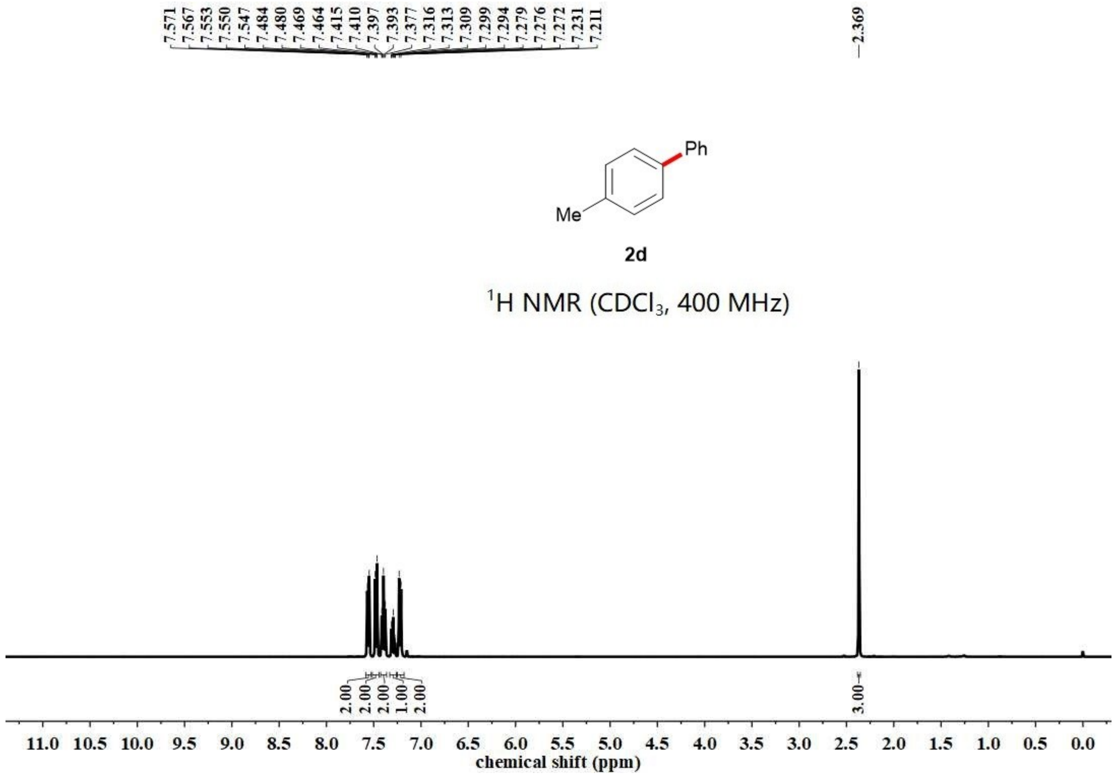

2d

^{1}H NMR (CDCl$_3$, 400 MHz)

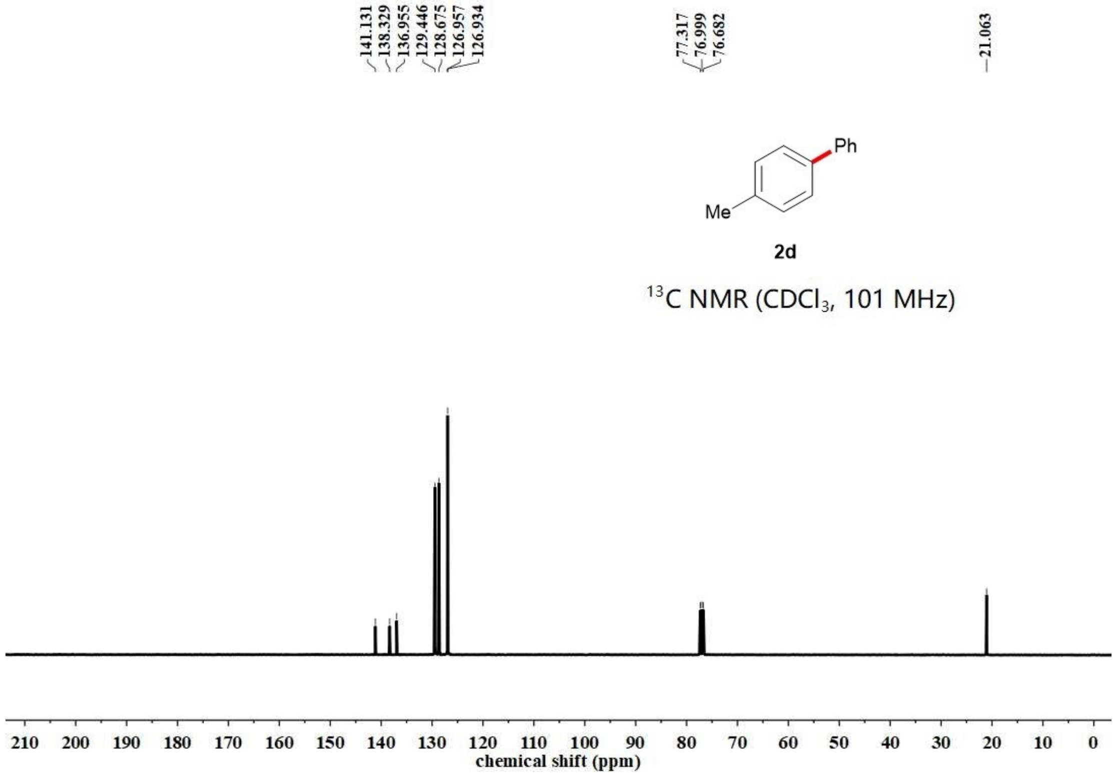

2d

^{13}C NMR (CDCl$_3$, 101 MHz)

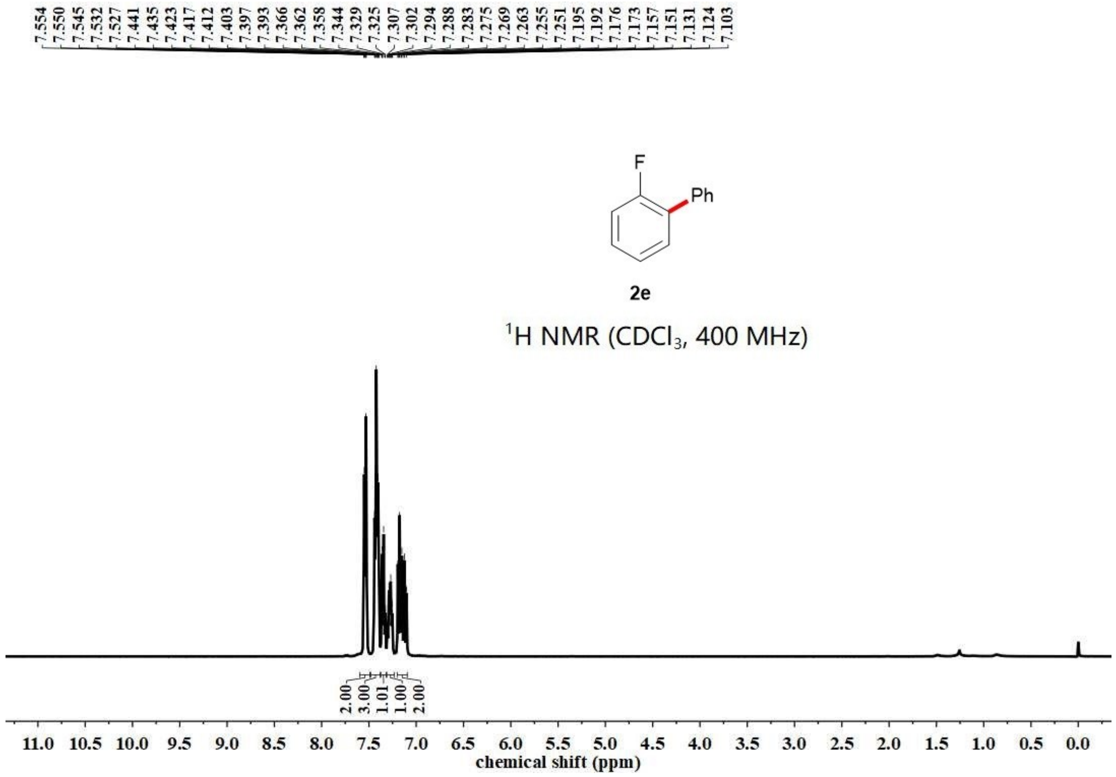
7.554 7.550 7.545 7.532 7.527 7.441 7.435 7.423 7.417 7.412 7.403 7.397 7.393 7.366 7.362 7.358 7.344 7.329 7.325 7.307 7.302 7.294 7.288 7.283 7.275 7.269 7.263 7.255 7.251 7.195 7.192 7.176 7.173 7.157 7.151 7.131 7.124 7.103
F
Ph
2e
^{1}H NMR (CDCl$_3$, 400 MHz)
2.00 3.00 1.01 1.00 2.00
11.0 10.5 10.0 9.5 9.0 8.5 8.0 7.5 7.0 6.5 6.0 5.5 5.0 4.5 4.0 3.5 3.0 2.5 2.0 1.5 1.0 0.5 0.0
chemical shift (ppm)

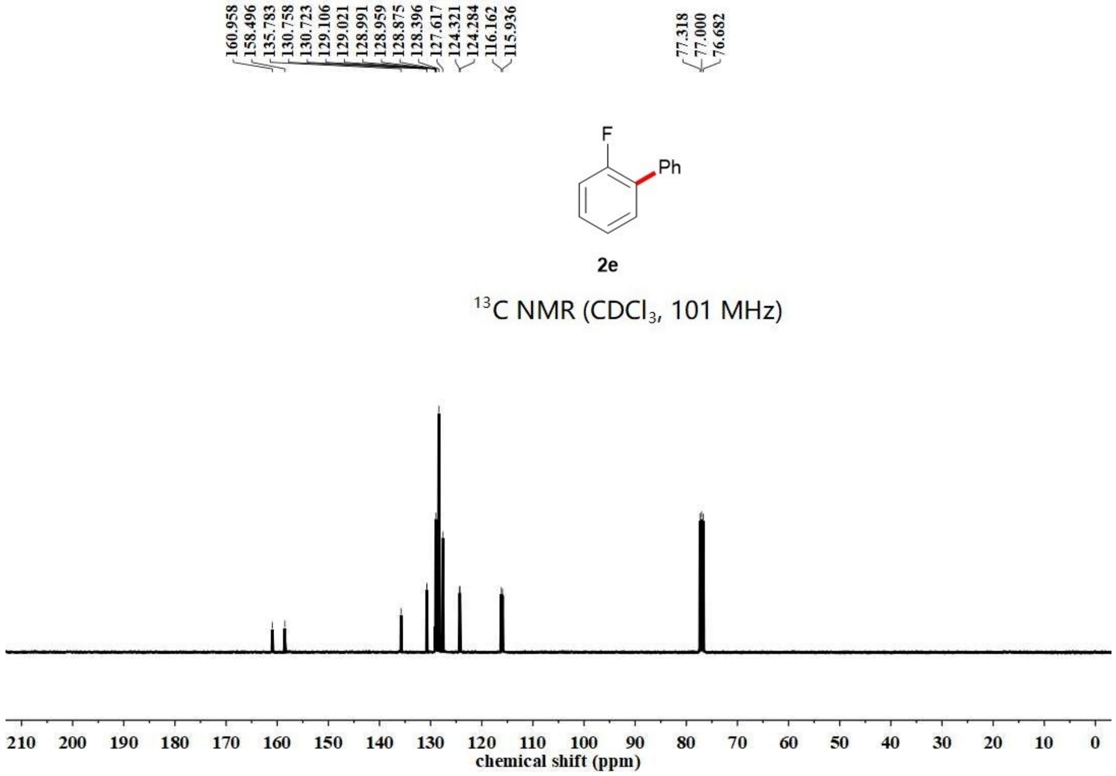
160.958 158.496 135.783 130.758 130.723 129.106 129.021 128.991 128.959 128.875 128.396 127.617 124.321 124.284 116.162 115.936
77.318 77.000 76.682
F
Ph
2e
^{13}C NMR (CDCl$_3$, 101 MHz)
210 200 190 180 170 160 150 140 130 120 110 100 90 80 70 60 50 40 30 20 10 0
chemical shift (ppm)

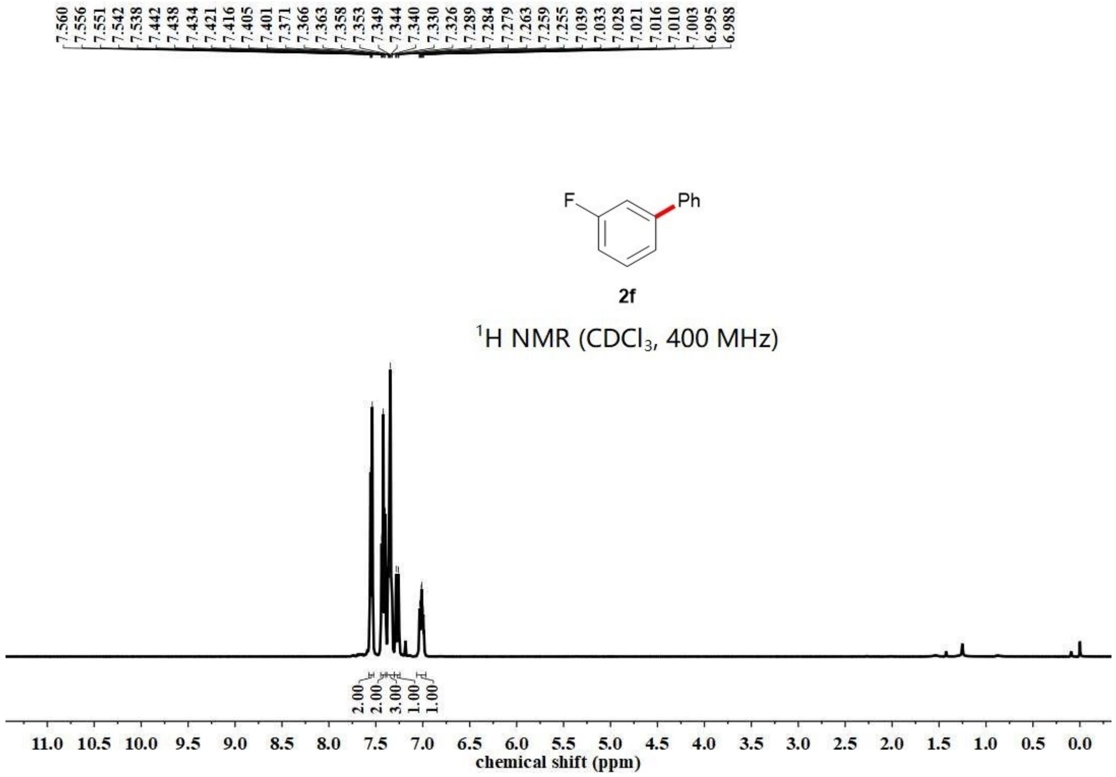
7.560 7.556 7.551 7.542 7.538 7.442 7.438 7.434 7.421 7.416 7.405 7.401 7.371 7.366 7.363 7.358 7.353 7.349 7.344 7.340 7.330 7.326 7.289 7.284 7.279 7.263 7.259 7.255 7.039 7.033 7.028 7.021 7.016 7.010 7.003 6.995 6.988
F
Ph
2f
^{1}H NMR (CDCl$_3$, 400 MHz)
2.00 2.00 3.00 1.00 1.00
11.0 10.5 10.0 9.5 9.0 8.5 8.0 7.5 7.0 6.5 6.0 5.5 5.0 4.5 4.0 3.5 3.0 2.5 2.0 1.5 1.0 0.5 0.0
chemical shift (ppm)

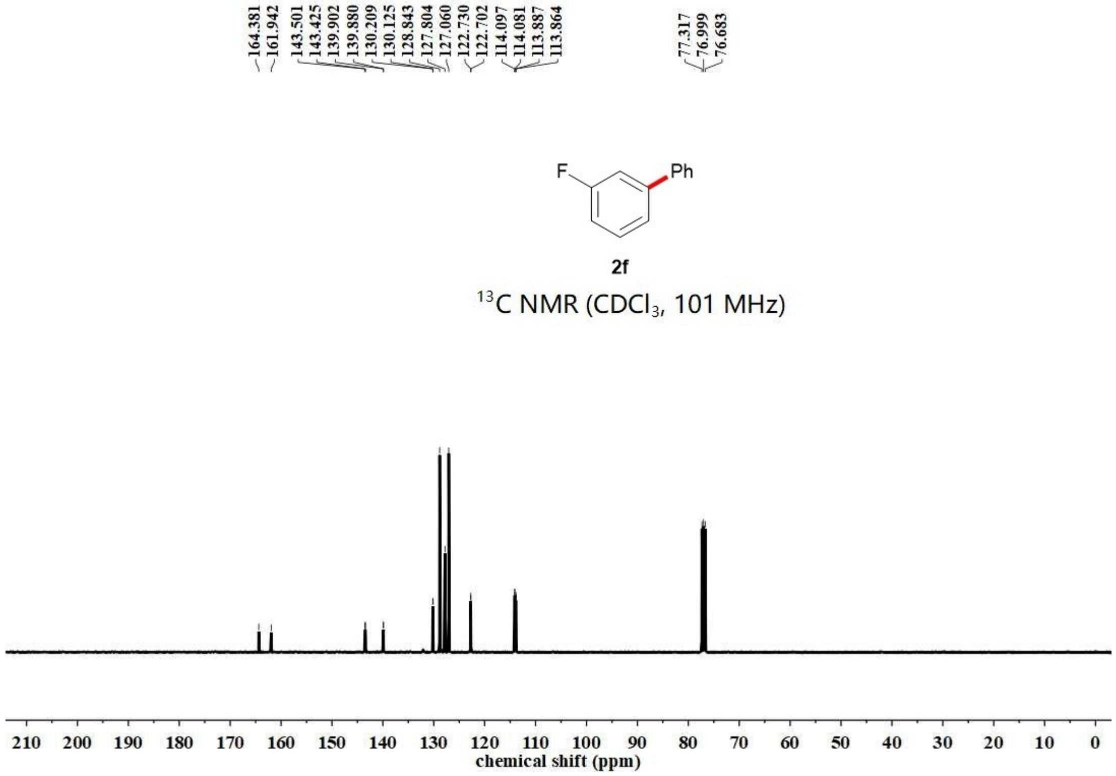
164.381 161.942 143.501 143.425 139.902 139.880 130.209 130.125 128.843 127.804 127.060 122.730 122.702 114.097 114.081 113.887 113.864
77.317 76.999 76.683
F
Ph
2f
^{13}C NMR (CDCl$_3$, 101 MHz)
210 200 190 180 170 160 150 140 130 120 110 100 90 80 70 60 50 40 30 20 10 0
chemical shift (ppm)

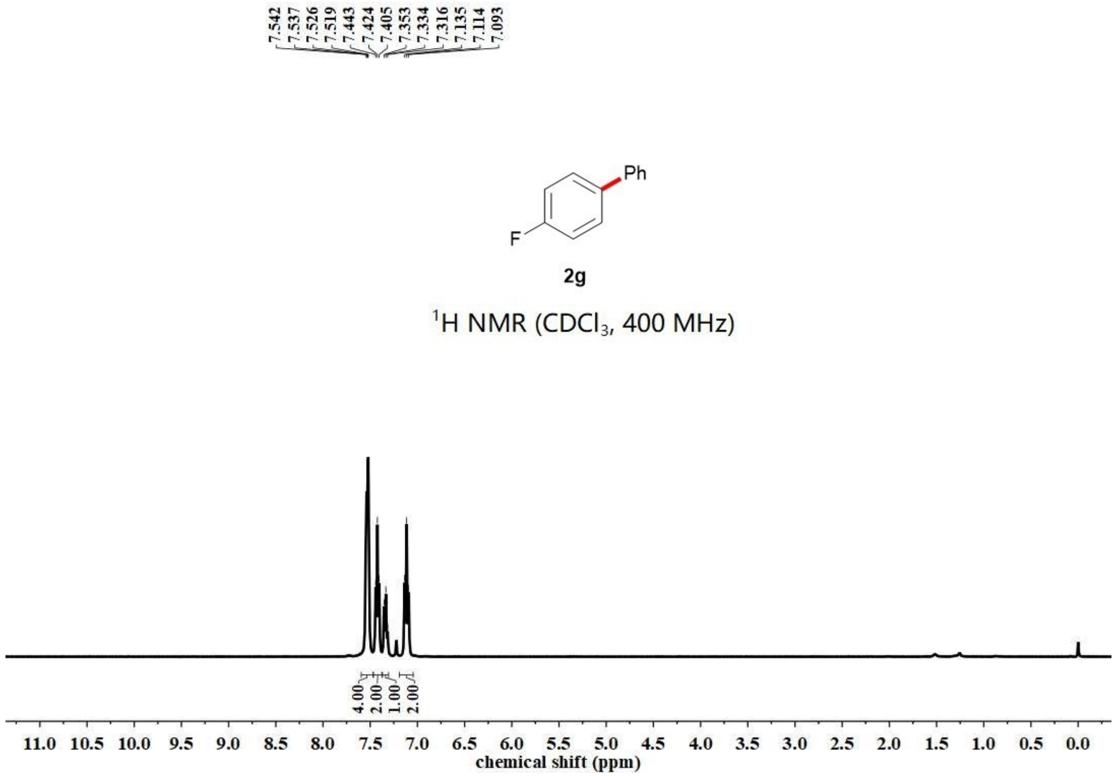
7.542
7.537
7.526
7.519
7.443
7.424
7.405
7.353
7.334
7.316
7.135
7.114
7.093
Ph
F
2g
^{1}H NMR (CDCl$_3$, 400 MHz)
4.00
2.00
1.00
2.00
11.0 10.5 10.0 9.5 9.0 8.5 8.0 7.5 7.0 6.5 6.0 5.5 5.0 4.5 4.0 3.5 3.0 2.5 2.0 1.5 1.0 0.5 0.0
chemical shift (ppm)

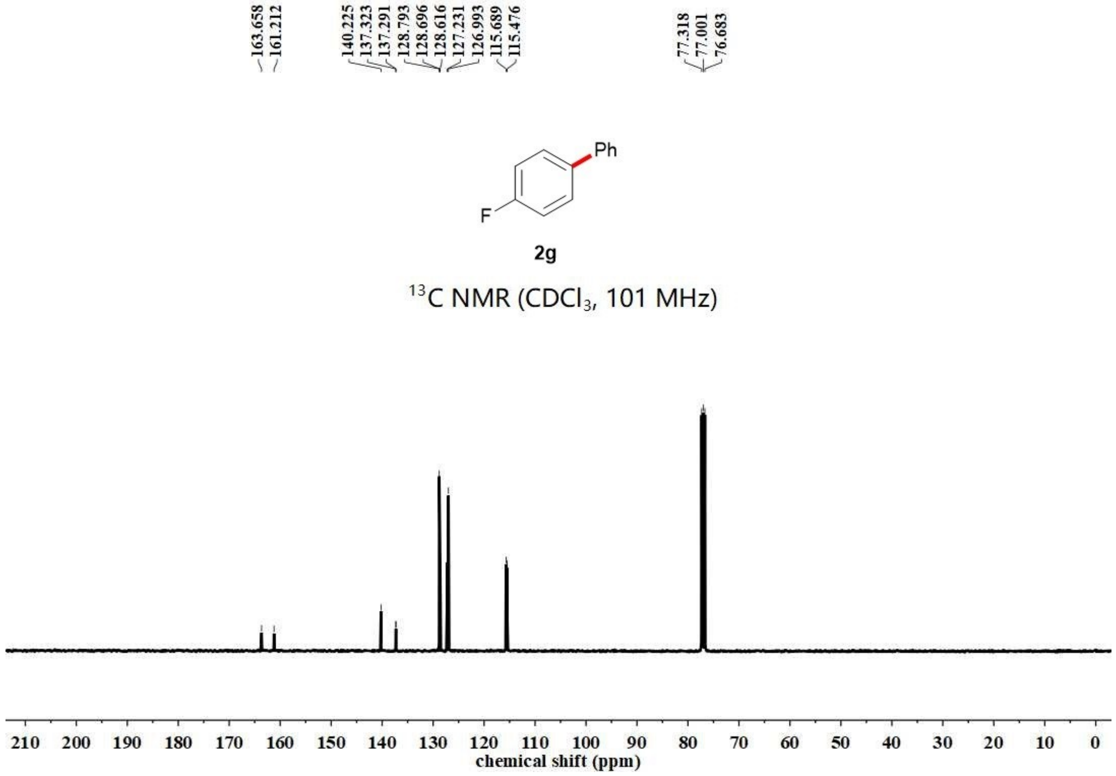
163.658
161.212
140.225
137.323
137.291
128.793
128.696
128.616
127.231
126.993
115.689
115.476
77.318
77.001
76.683
Ph
F
2g
^{13}C NMR (CDCl$_3$, 101 MHz)
210 200 190 180 170 160 150 140 130 120 110 100 90 80 70 60 50 40 30 20 10 0
chemical shift (ppm)

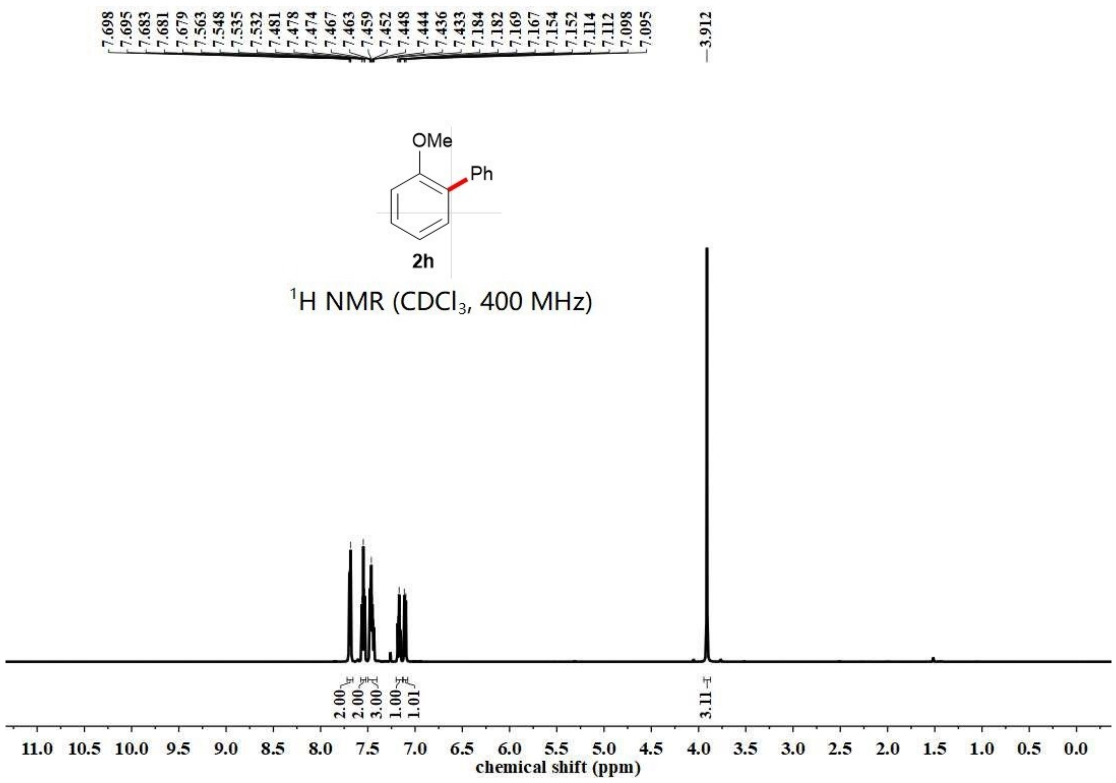
7.698 7.695 7.683 7.681 7.679 7.563 7.548 7.535 7.532 7.481 7.478 7.474 7.467 7.463 7.459 7.452 7.448 7.444 7.436 7.433 7.184 7.182 7.169 7.167 7.154 7.152 7.114 7.112 7.098 7.095
3.912
OMe
Ph
2h
1H NMR (CDCl3, 400 MHz)
2.00 2.00 3.00 1.00 1.01
3.11
11.0 10.5 10.0 9.5 9.0 8.5 8.0 7.5 7.0 6.5 6.0 5.5 5.0 4.5 4.0 3.5 3.0 2.5 2.0 1.5 1.0 0.5 0.0
chemical shift (ppm)

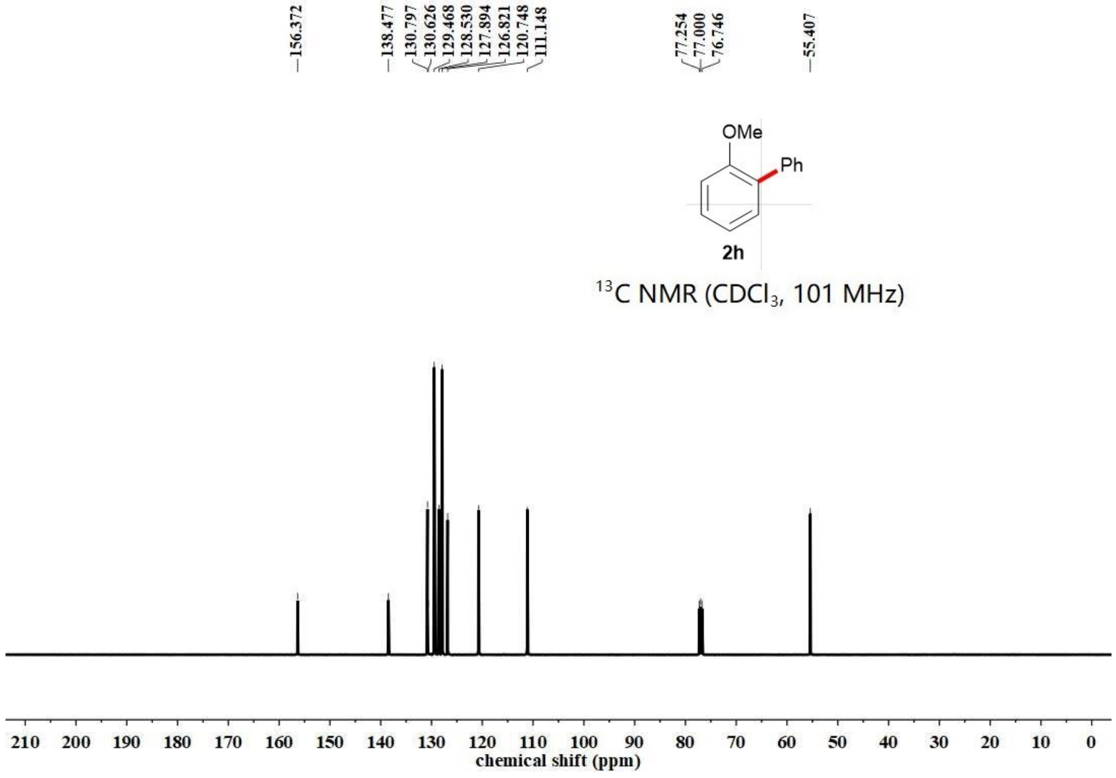
156.372
138.477 130.797 130.626 129.468 128.530 127.894 126.821 120.748 111.148
77.254 77.000 76.746
55.407
OMe
Ph
2h
13C NMR (CDCl3, 101 MHz)
210 200 190 180 170 160 150 140 130 120 110 100 90 80 70 60 50 40 30 20 10 0
chemical shift (ppm)

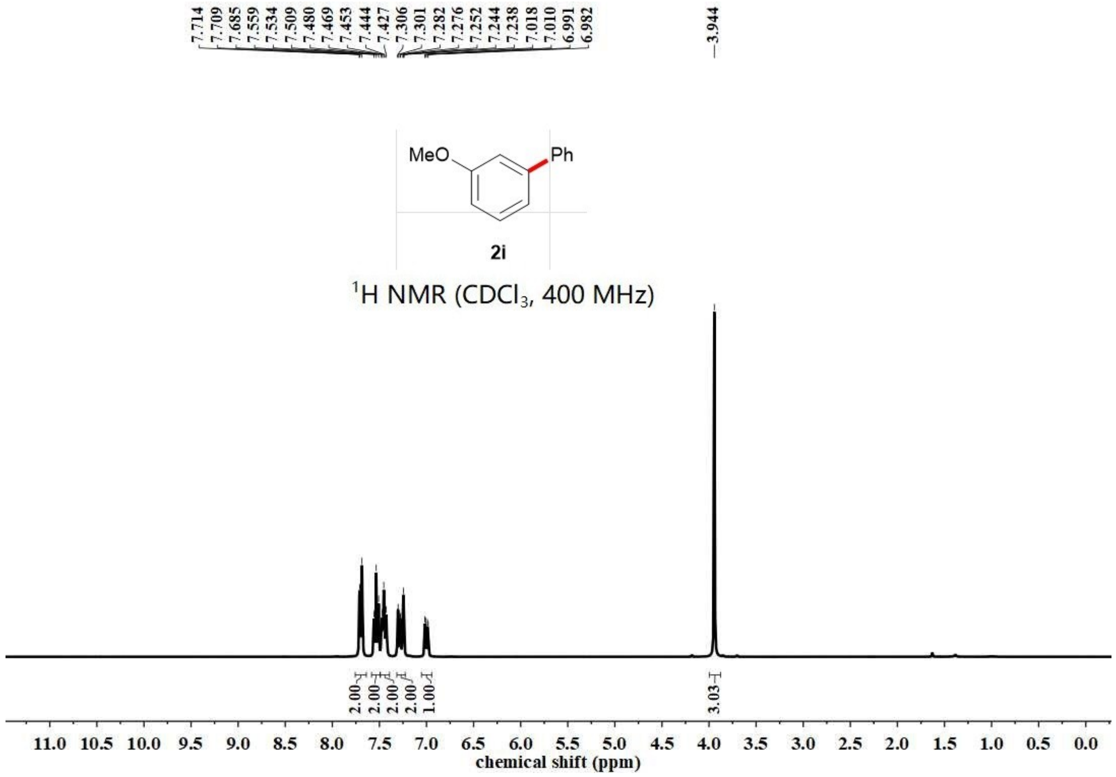
7.714
7.709
7.685
7.559
7.534
7.509
7.480
7.469
7.453
7.444
7.427
7.306
7.301
7.282
7.276
7.252
7.244
7.238
7.018
7.010
6.991
6.982
3.944
MeO
Ph
2i
1H NMR (CDCl3, 400 MHz)
2.00
2.00
2.00
2.00
1.00
3.03
11.0 10.5 10.0 9.5 9.0 8.5 8.0 7.5 7.0 6.5 6.0 5.5 5.0 4.5 4.0 3.5 3.0 2.5 2.0 1.5 1.0 0.5 0.0
chemical shift (ppm)

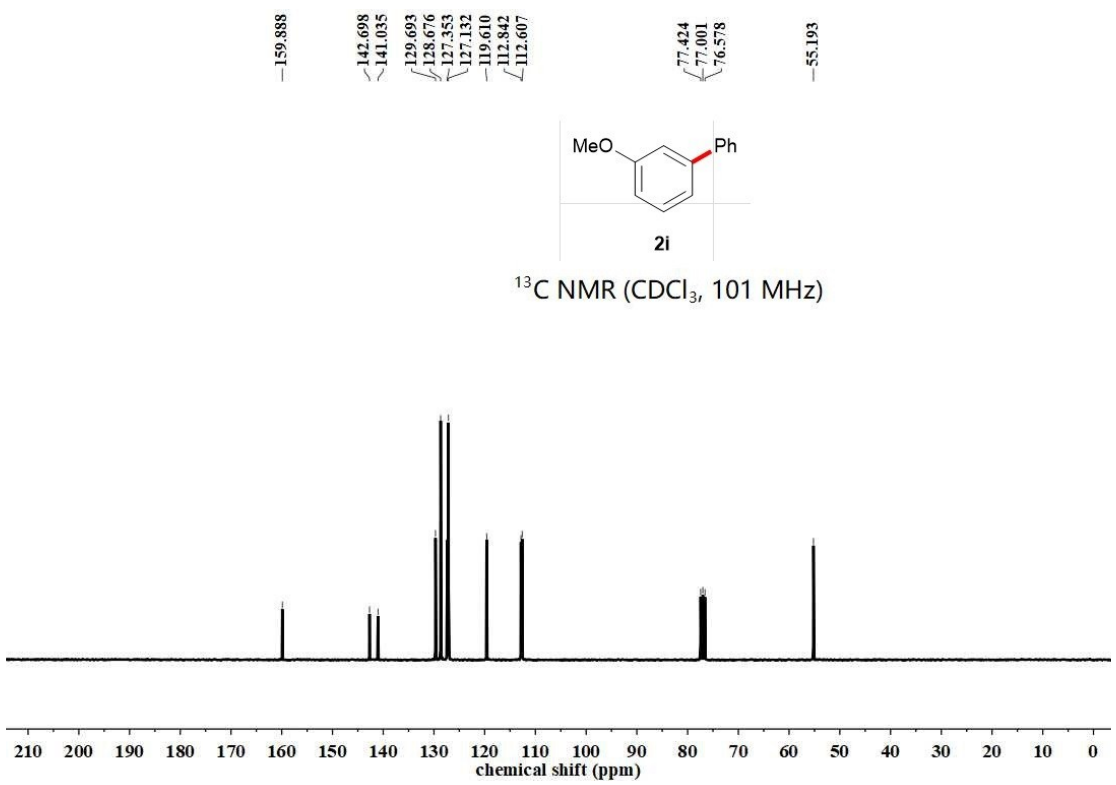
159.888
142.698
141.035
129.693
128.676
127.353
127.132
119.610
112.842
112.607
77.424
77.001
76.578
55.193
MeO
Ph
2i
13C NMR (CDCl3, 101 MHz)
210 200 190 180 170 160 150 140 130 120 110 100 90 80 70 60 50 40 30 20 10 0
chemical shift (ppm)

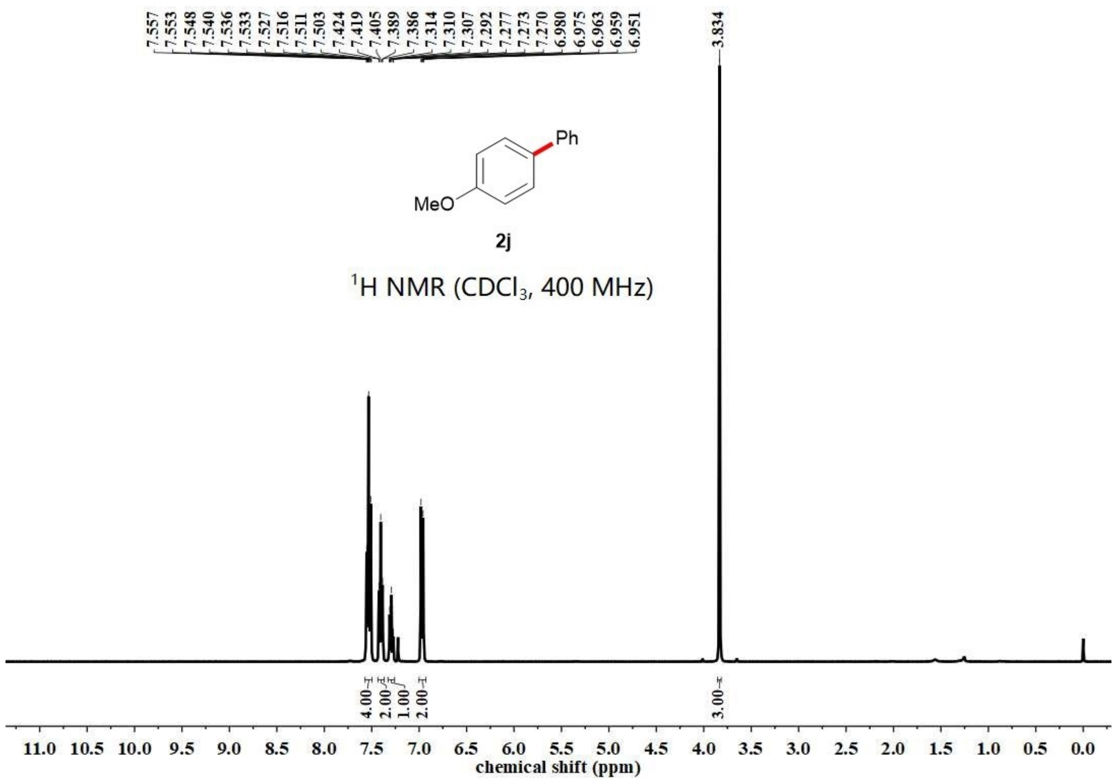

7.557
7.553
7.548
7.540
7.536
7.533
7.527
7.516
7.511
7.503
7.424
7.419
7.405
7.389
7.386
7.314
7.310
7.307
7.292
7.277
7.273
7.270
6.980
6.975
6.963
6.959
6.951
3.834
Ph
MeO
2j
^{1}H NMR (CDCl$_3$, 400 MHz)
4.00
2.00
1.00
2.00
3.00
11.0 10.5 10.0 9.5 9.0 8.5 8.0 7.5 7.0 6.5 6.0 5.5 5.0 4.5 4.0 3.5 3.0 2.5 2.0 1.5 1.0 0.5 0.0
chemical shift (ppm)

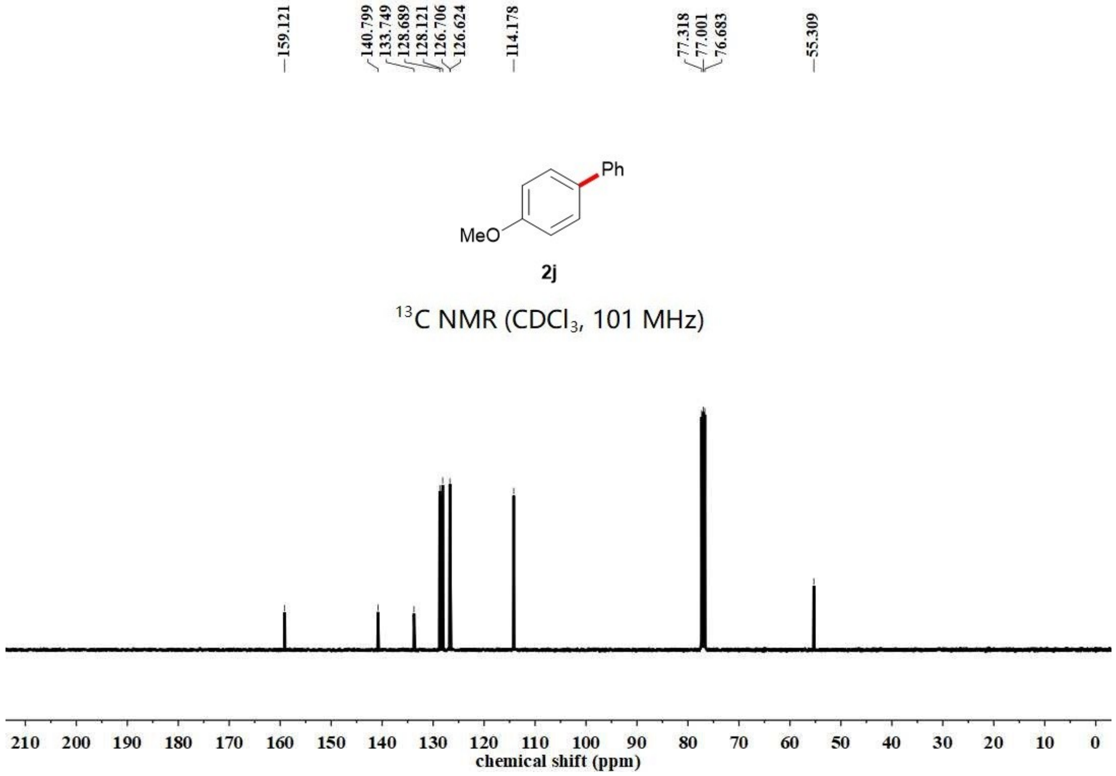

159.121
140.799
133.749
128.689
128.121
126.706
126.624
114.178
77.318
77.001
76.683
55.309
Ph
MeO
2j
^{13}C NMR (CDCl$_3$, 101 MHz)
210 200 190 180 170 160 150 140 130 120 110 100 90 80 70 60 50 40 30 20 10 0
chemical shift (ppm)

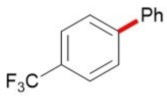

2k

^{1}H NMR (CDCl$_3$, 400 MHz)

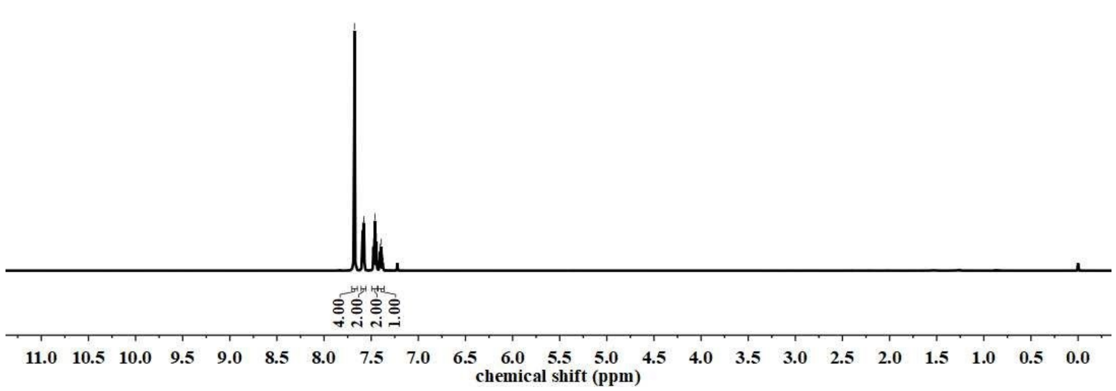

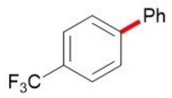

2k

^{13}C NMR (CDCl$_3$, 101 MHz)

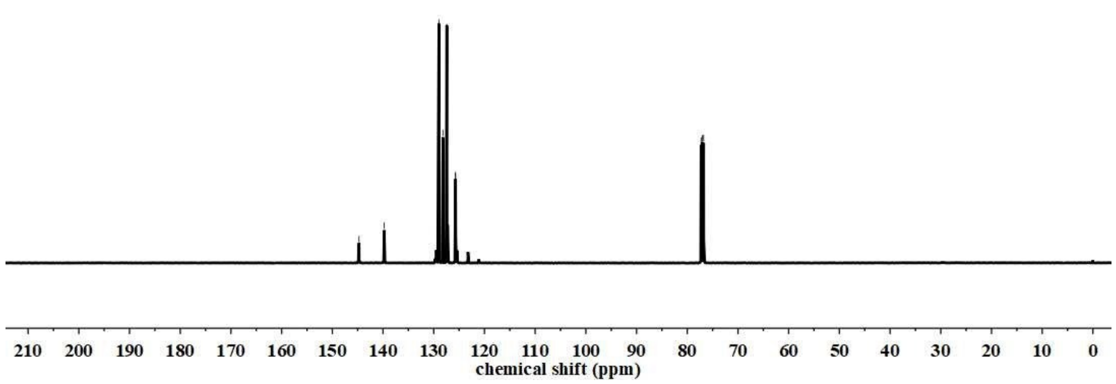

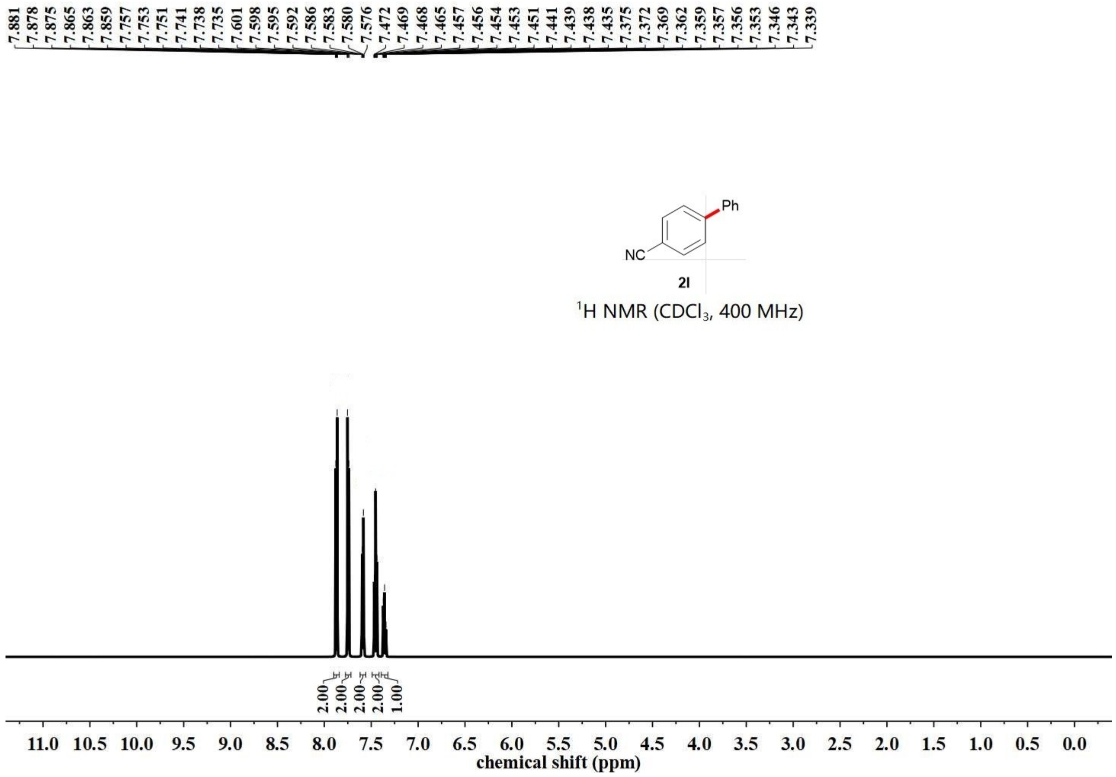
7.881 7.878 7.875 7.865 7.863 7.859 7.757 7.753 7.751 7.741 7.738 7.735 7.601 7.598 7.595 7.592 7.586 7.583 7.580 7.576 7.472 7.469 7.468 7.465 7.457 7.456 7.454 7.453 7.451 7.441 7.439 7.438 7.435 7.375 7.372 7.369 7.362 7.359 7.357 7.356 7.353 7.346 7.343 7.339
Ph
NC
2l
¹H NMR (CDCl₃, 400 MHz)
2.00 2.00 2.00 2.00 1.00
11.0 10.5 10.0 9.5 9.0 8.5 8.0 7.5 7.0 6.5 6.0 5.5 5.0 4.5 4.0 3.5 3.0 2.5 2.0 1.5 1.0 0.5 0.0
chemical shift (ppm)

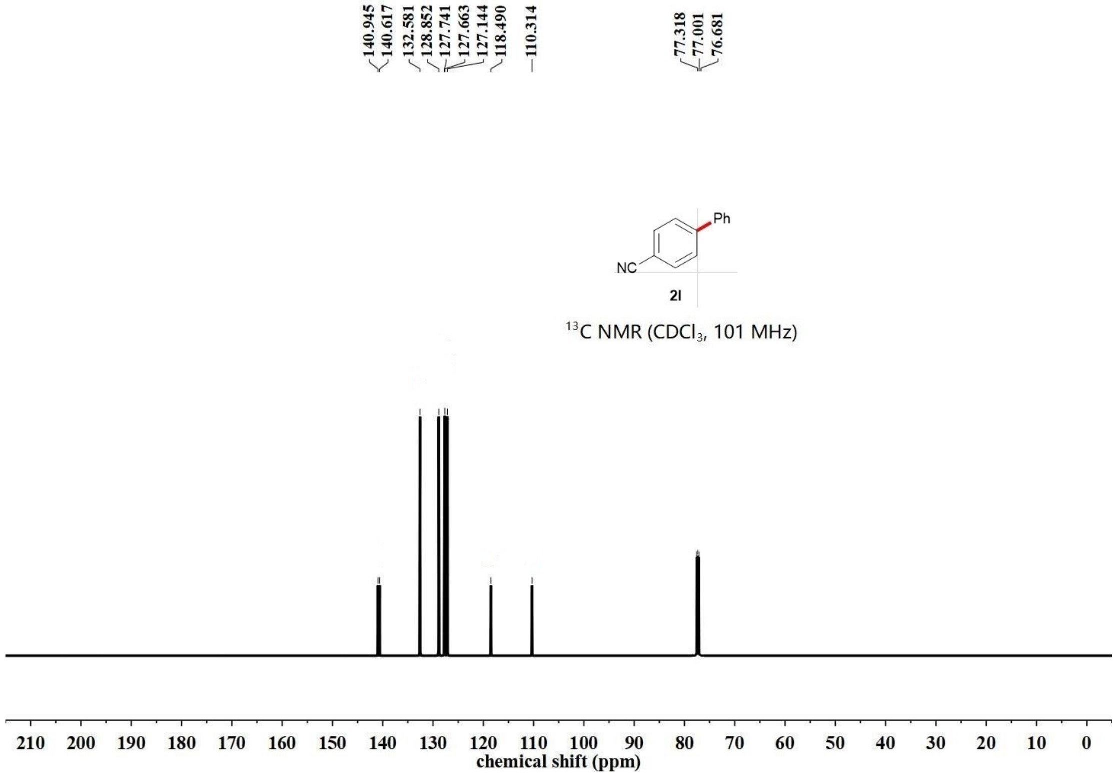
140.945 140.617 132.581 128.852 127.741 127.663 127.144 118.490 110.314
77.318 77.001 76.681
Ph
NC
2l
¹³C NMR (CDCl₃, 101 MHz)
210 200 190 180 170 160 150 140 130 120 110 100 90 80 70 60 50 40 30 20 10 0
chemical shift (ppm)

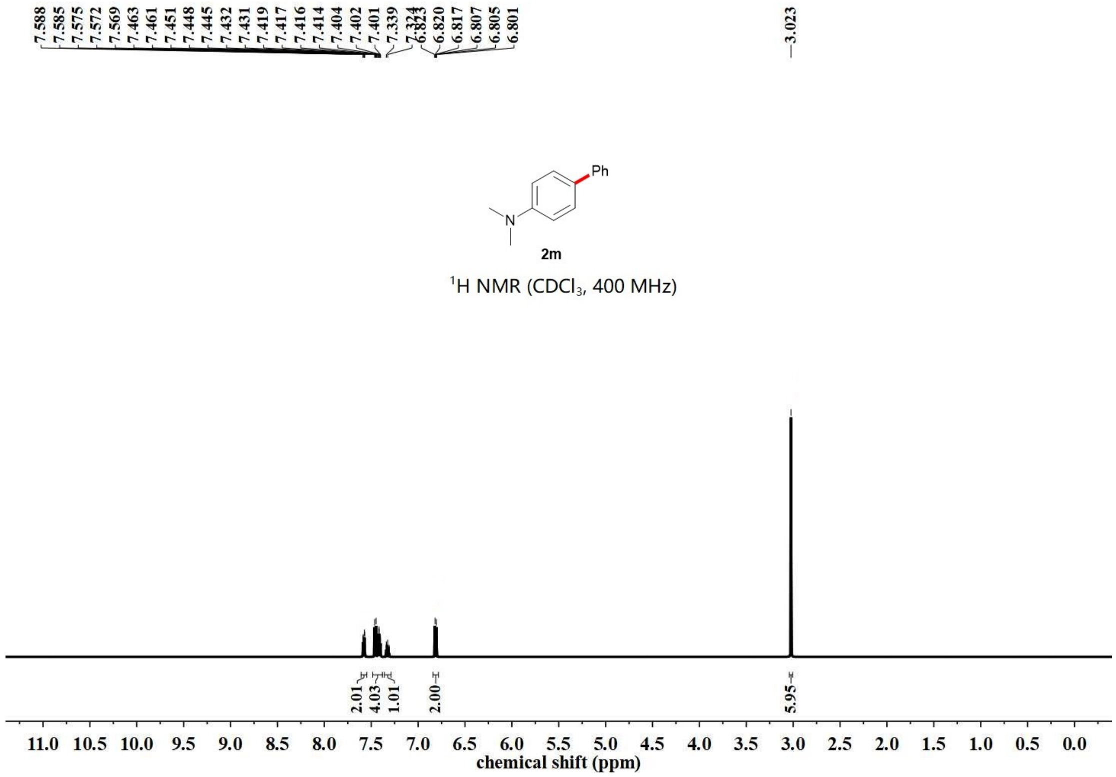
7.588
7.585
7.575
7.572
7.569
7.463
7.461
7.451
7.448
7.445
7.432
7.431
7.419
7.417
7.416
7.414
7.404
7.402
7.401
7.339
6.820
6.817
6.807
6.805
6.801
3.023
Ph
N
2m
¹H NMR (CDCl₃, 400 MHz)
2.01
4.03
1.01
2.00
5.95
11.0 10.5 10.0 9.5 9.0 8.5 8.0 7.5 7.0 6.5 6.0 5.5 5.0 4.5 4.0 3.5 3.0 2.5 2.0 1.5 1.0 0.5 0.0
chemical shift (ppm)

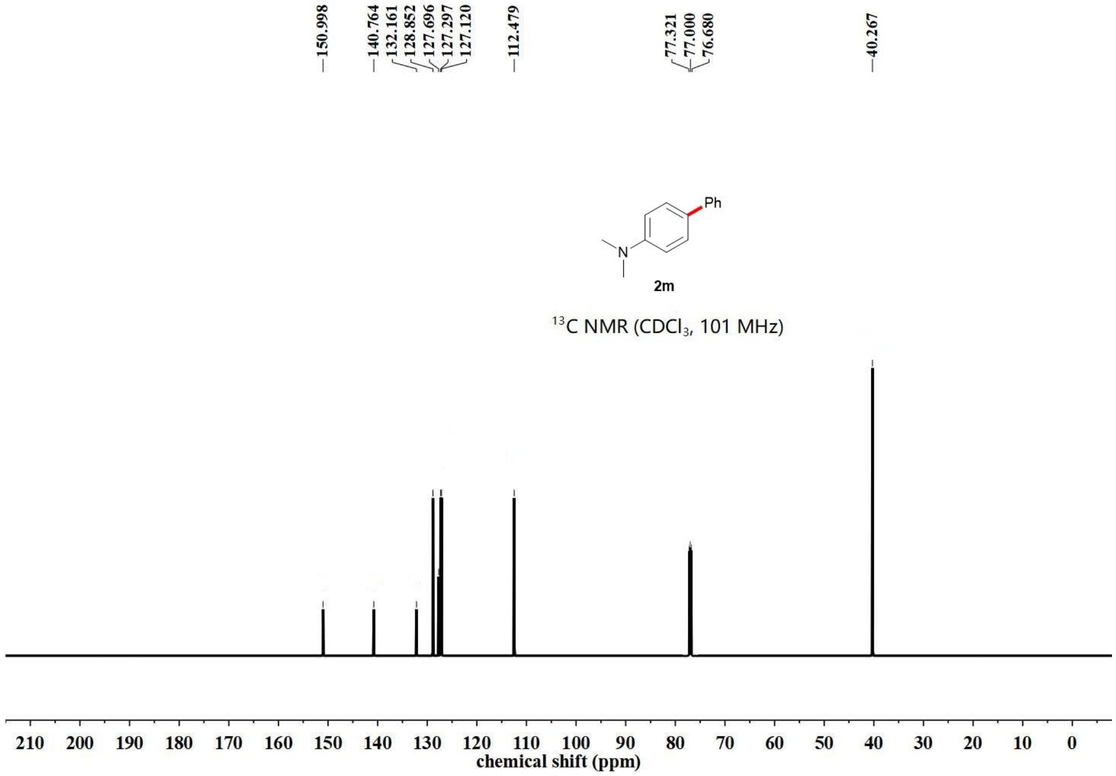
150.998
140.764
132.161
128.852
127.696
127.297
127.120
112.479
77.321
77.000
76.680
40.267
Ph
N
2m
¹³C NMR (CDCl₃, 101 MHz)
210 200 190 180 170 160 150 140 130 120 110 100 90 80 70 60 50 40 30 20 10 0
chemical shift (ppm)

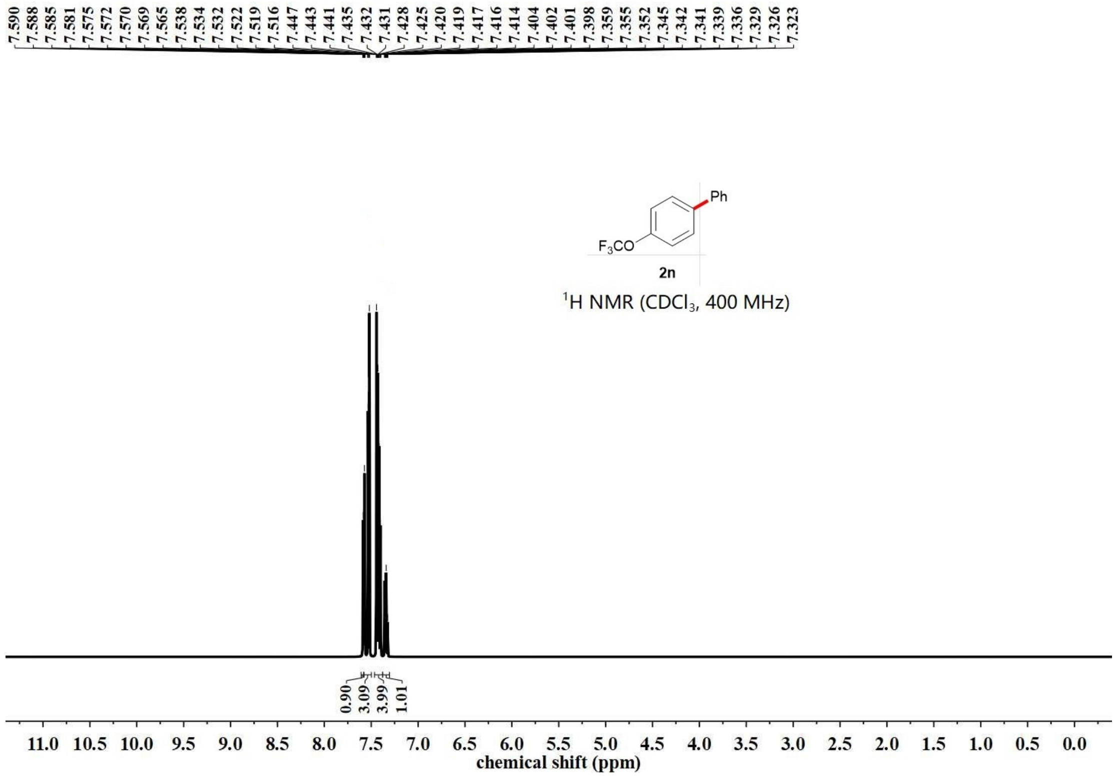
7.590 7.588 7.585 7.581 7.575 7.572 7.570 7.569 7.565 7.538 7.534 7.532 7.522 7.519 7.516 7.447 7.443 7.441 7.435 7.432 7.431 7.428 7.425 7.420 7.419 7.417 7.416 7.414 7.404 7.402 7.401 7.398 7.359 7.355 7.352 7.345 7.342 7.341 7.339 7.336 7.329 7.326 7.323
Ph
F3CO
2n
1H NMR (CDCl3, 400 MHz)
0.90
3.09
3.99
1.01
11.0 10.5 10.0 9.5 9.0 8.5 8.0 7.5 7.0 6.5 6.0 5.5 5.0 4.5 4.0 3.5 3.0 2.5 2.0 1.5 1.0 0.5 0.0
chemical shift (ppm)

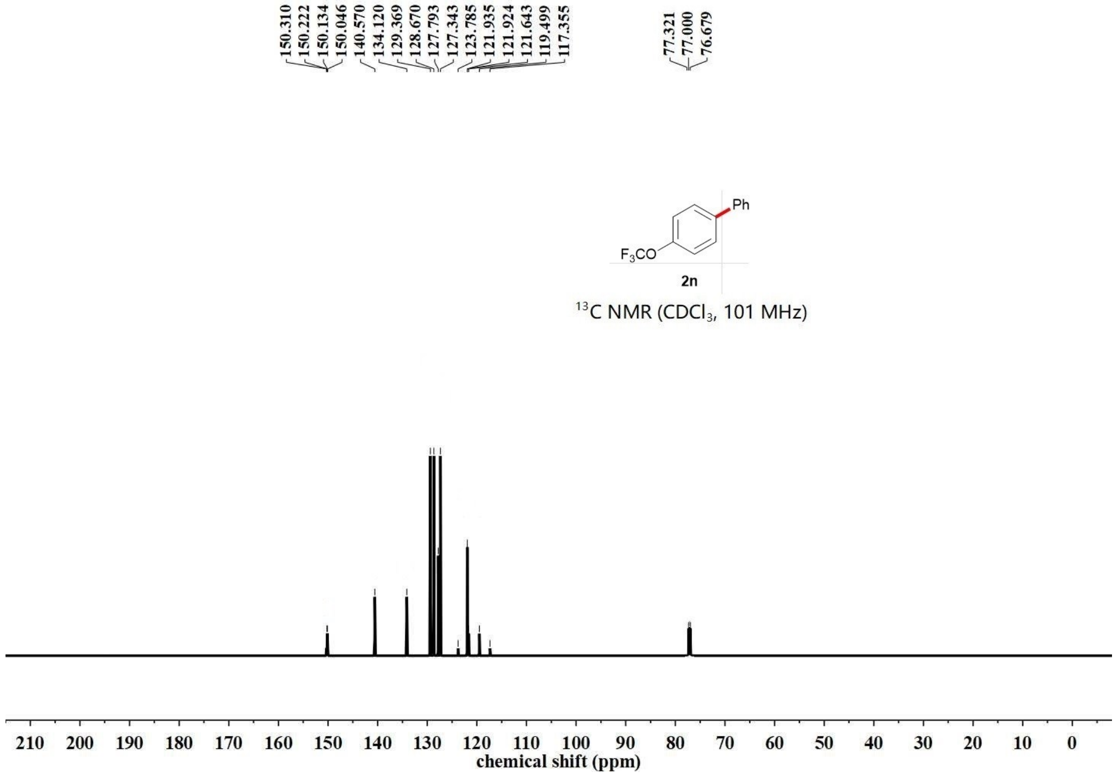
150.310 150.222 150.134 150.046 140.570 134.120 129.369 128.670 127.793 127.343 123.785 121.935 121.924 121.643 119.499 117.355
77.321 77.000 76.679
Ph
F3CO
2n
13C NMR (CDCl3, 101 MHz)
210 200 190 180 170 160 150 140 130 120 110 100 90 80 70 60 50 40 30 20 10 0
chemical shift (ppm)

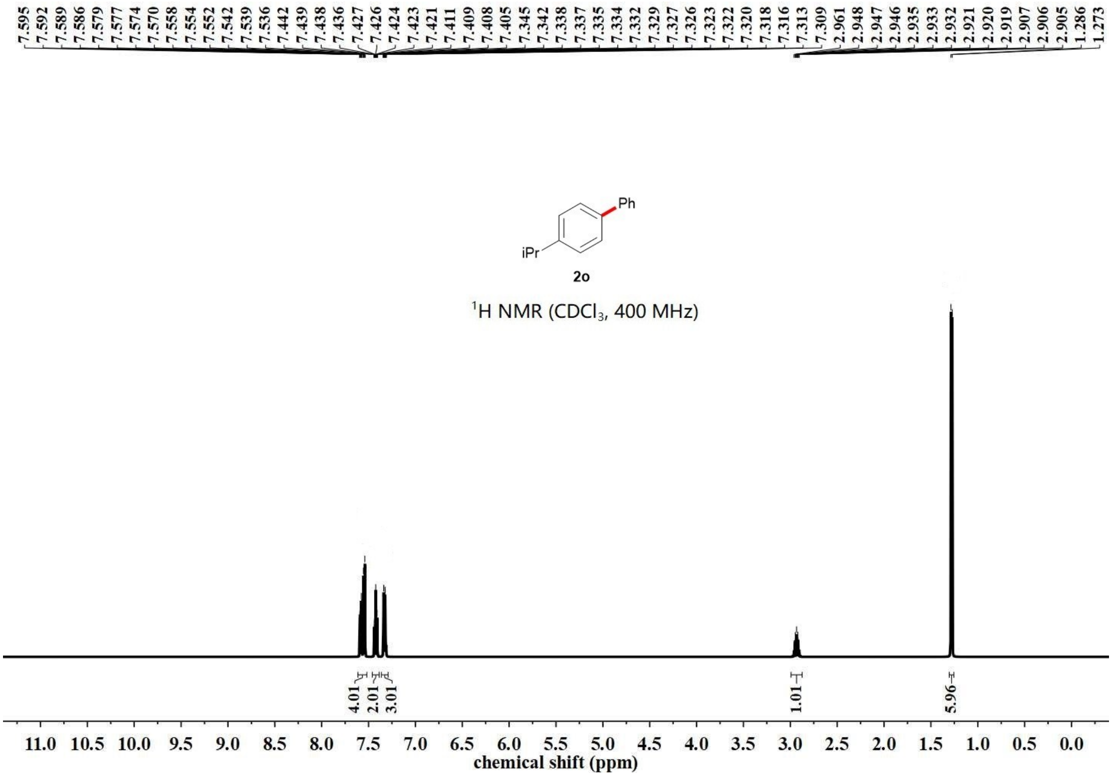
7.595 7.592 7.589 7.586 7.579 7.577 7.574 7.570 7.558 7.554 7.552 7.542 7.539 7.536 7.442 7.439 7.438 7.436 7.427 7.426 7.424 7.423 7.421 7.411 7.409 7.408 7.405 7.345 7.342 7.338 7.337 7.335 7.334 7.332 7.329 7.327 7.326 7.323 7.322 7.320 7.318 7.316 7.313 7.309 2.961 2.948 2.947 2.946 2.935 2.933 2.932 2.921 2.920 2.919 2.907 2.906 2.905 1.286 1.273
Ph
iPr
2o
1H NMR (CDCl3, 400 MHz)
4.01
2.01
3.01
1.01
5.96
11.0 10.5 10.0 9.5 9.0 8.5 8.0 7.5 7.0 6.5 6.0 5.5 5.0 4.5 4.0 3.5 3.0 2.5 2.0 1.5 1.0 0.5 0.0
chemical shift (ppm)

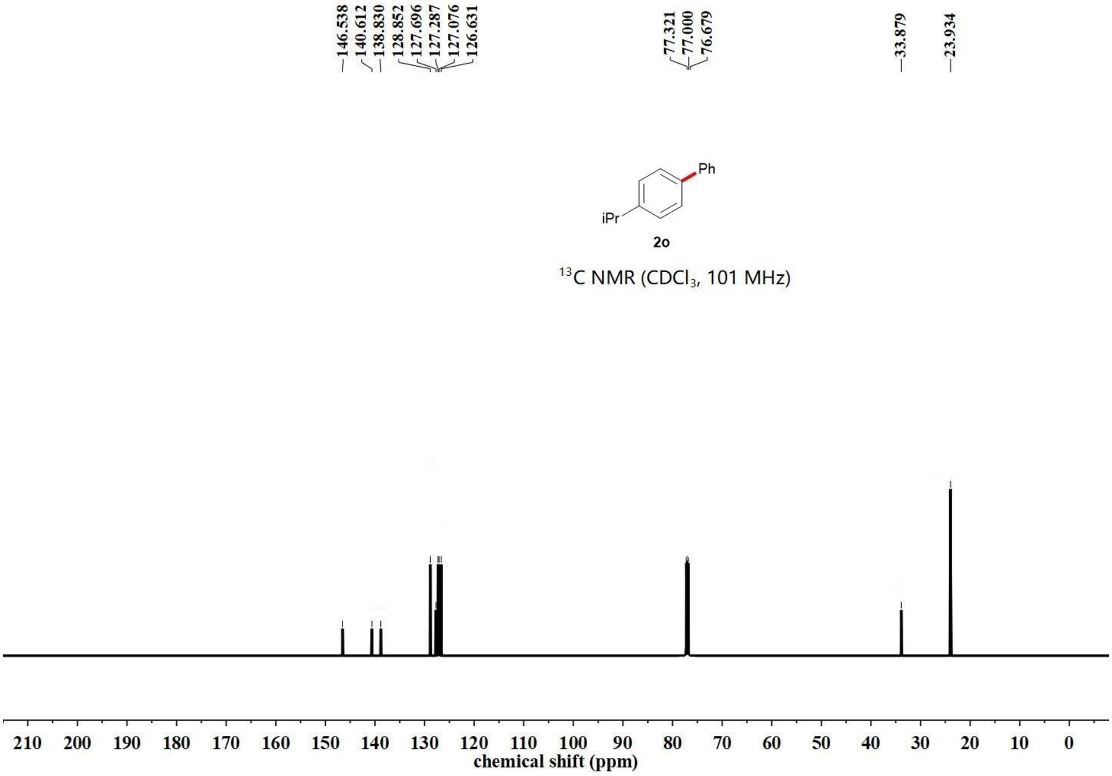
146.538 140.612 138.830 128.852 127.696 127.287 127.076 126.631
77.321 77.000 76.679
33.879
23.934
Ph
iPr
2o
13C NMR (CDCl3, 101 MHz)
210 200 190 180 170 160 150 140 130 120 110 100 90 80 70 60 50 40 30 20 10 0
chemical shift (ppm)

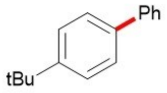

2p

^{1}H NMR ($CDCl_3$, 400 MHz)

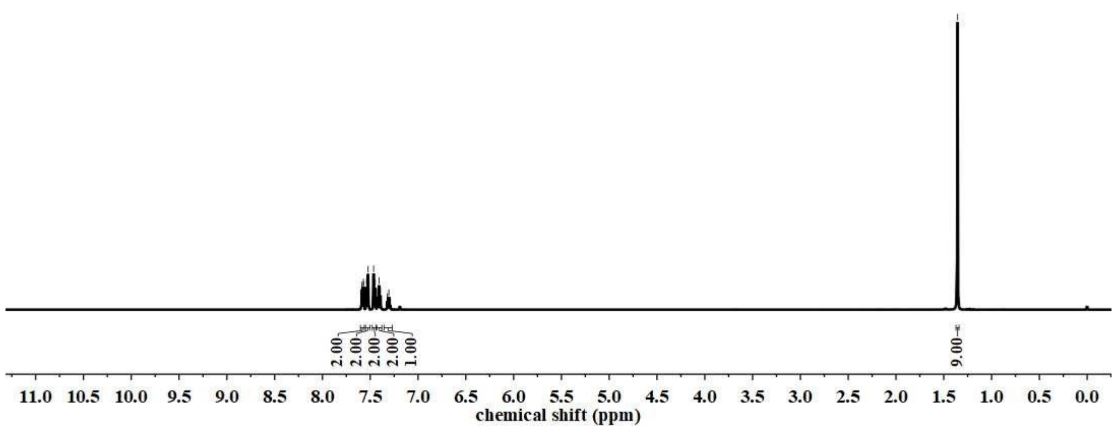

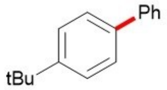

2p

^{13}C NMR ($CDCl_3$, 101 MHz)

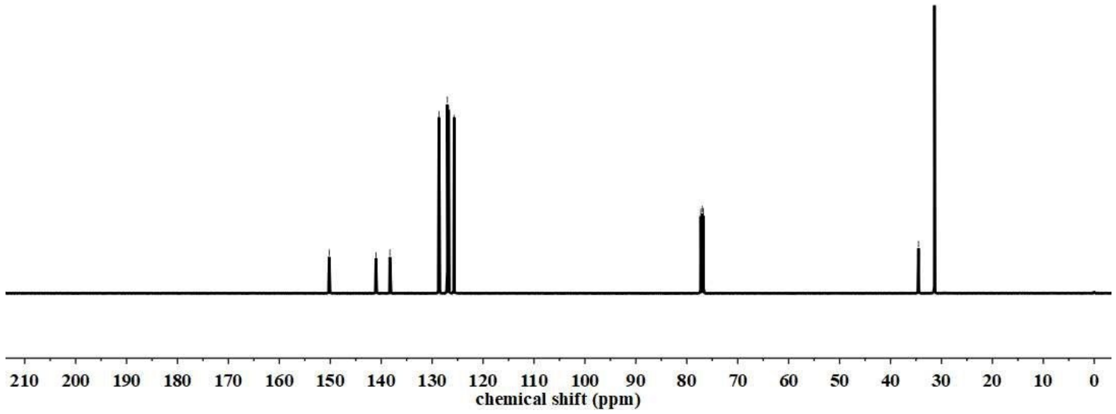

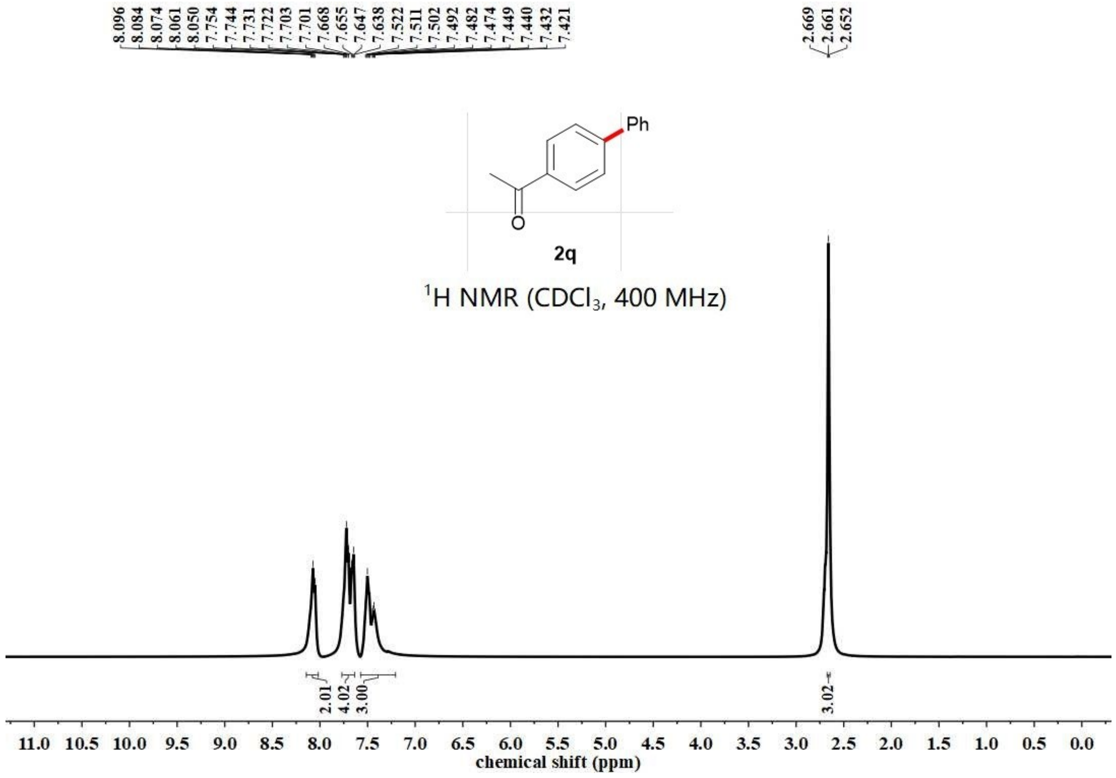
8.096
8.084
8.074
8.061
8.050
7.754
7.744
7.731
7.722
7.703
7.701
7.668
7.655
7.647
7.638
7.522
7.511
7.502
7.492
7.482
7.474
7.449
7.440
7.432
7.421
2.669
2.661
2.652
Ph
O
2q
^{1}H NMR (CDCl$_3$, 400 MHz)
2.01
4.02
3.00
3.02
11.0 10.5 10.0 9.5 9.0 8.5 8.0 7.5 7.0 6.5 6.0 5.5 5.0 4.5 4.0 3.5 3.0 2.5 2.0 1.5 1.0 0.5 0.0
chemical shift (ppm)

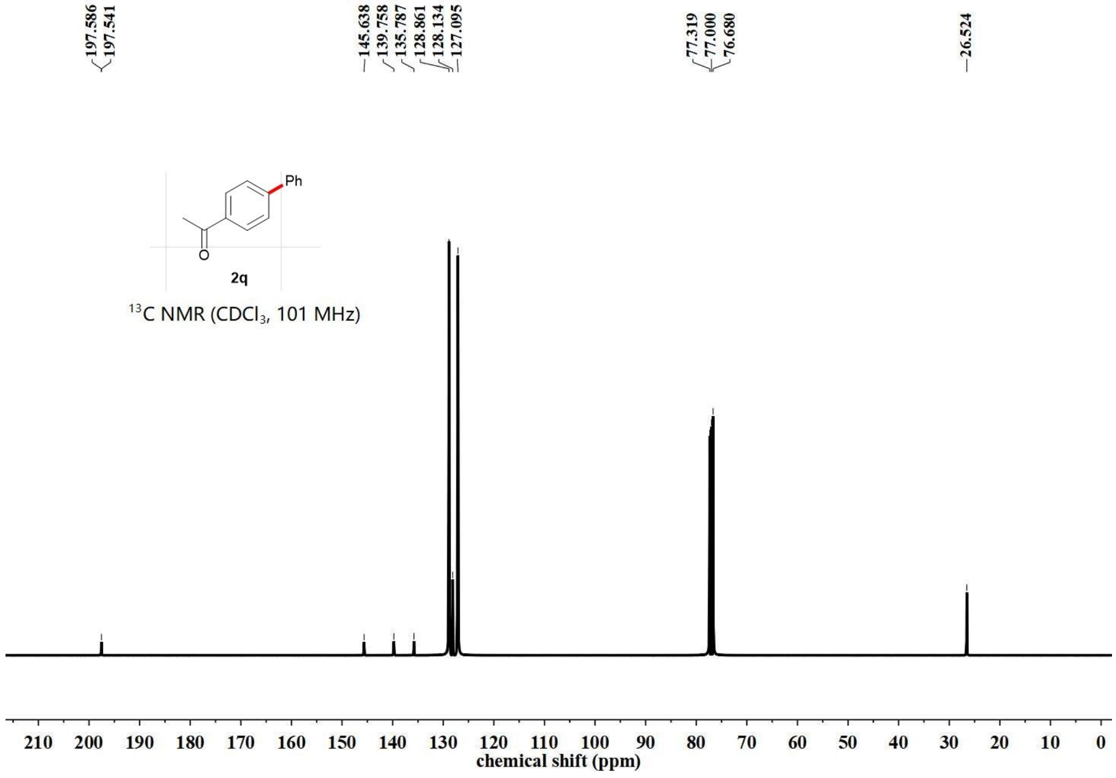
197.586
197.541
145.638
139.758
135.787
128.861
128.134
127.095
77.319
77.000
76.680
26.524
Ph
O
2q
^{13}C NMR (CDCl$_3$, 101 MHz)
210 200 190 180 170 160 150 140 130 120 110 100 90 80 70 60 50 40 30 20 10 0
chemical shift (ppm)

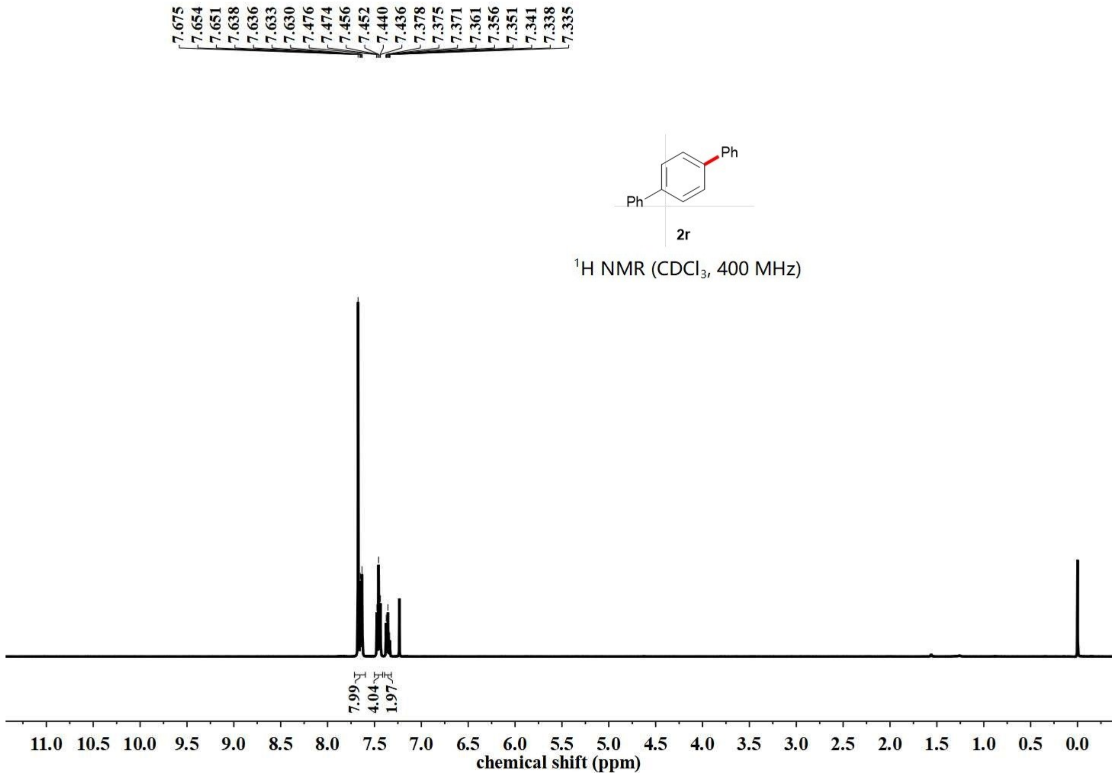
7.675
7.654
7.651
7.638
7.636
7.633
7.630
7.476
7.474
7.456
7.452
7.440
7.436
7.378
7.375
7.371
7.361
7.356
7.351
7.341
7.338
7.335
Ph
Ph
2r
^{1}H NMR (CDCl$_3$, 400 MHz)
7.99
4.04
1.97
11.0 10.5 10.0 9.5 9.0 8.5 8.0 7.5 7.0 6.5 6.0 5.5 5.0 4.5 4.0 3.5 3.0 2.5 2.0 1.5 1.0 0.5 0.0
chemical shift (ppm)

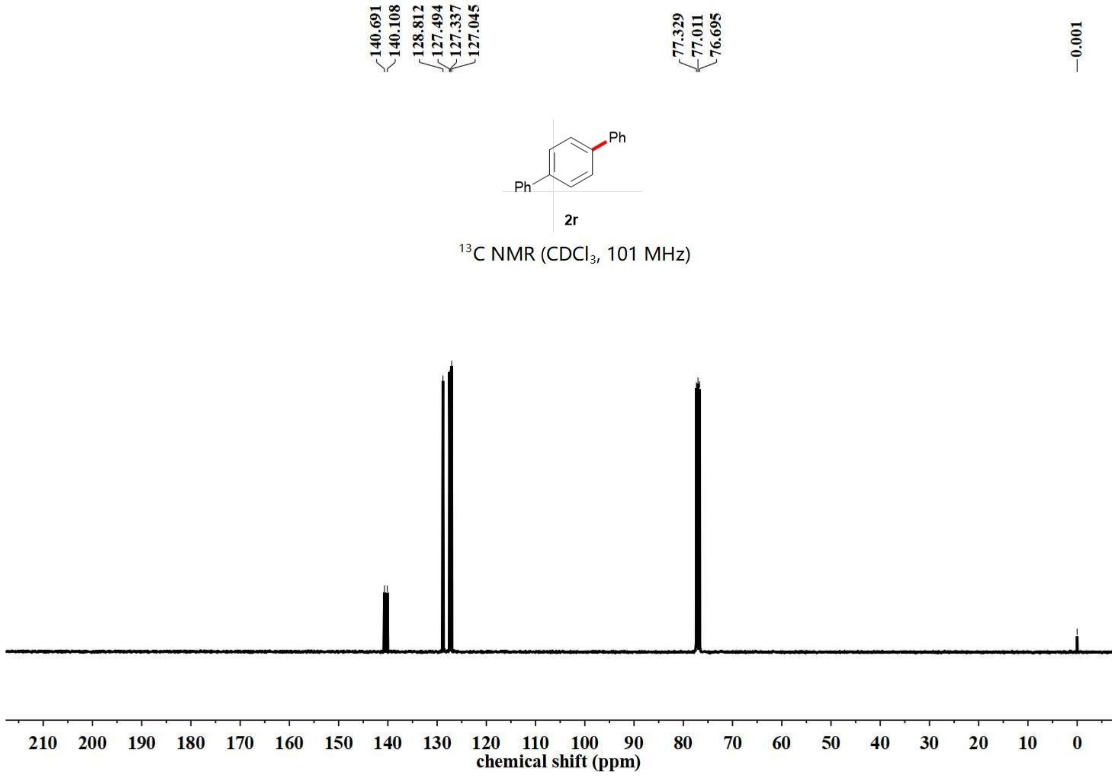
140.691
140.108
128.812
127.494
127.337
127.045
77.329
77.011
76.695
0.001
Ph
Ph
2r
^{13}C NMR (CDCl$_3$, 101 MHz)
210 200 190 180 170 160 150 140 130 120 110 100 90 80 70 60 50 40 30 20 10 0
chemical shift (ppm)

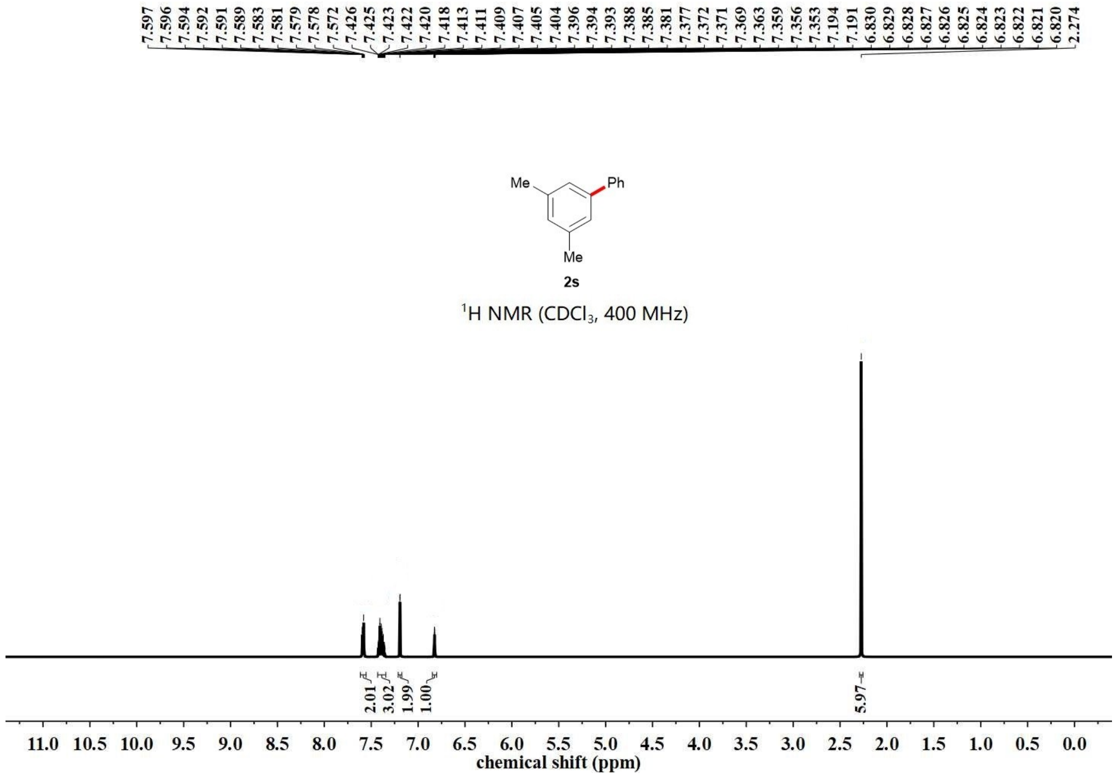
7.597 7.596 7.594 7.592 7.591 7.589 7.583 7.581 7.579 7.578 7.572 7.426 7.425 7.423 7.422 7.420 7.418 7.413 7.411 7.409 7.407 7.405 7.404 7.396 7.394 7.393 7.388 7.385 7.381 7.377 7.372 7.371 7.369 7.363 7.359 7.356 7.353 7.194 7.191 6.830 6.829 6.828 6.827 6.826 6.825 6.824 6.823 6.822 6.821 6.820 2.274
Me
Ph
Me
2s
^{1}H NMR (CDCl$_3$, 400 MHz)
2.01
3.02
1.99
1.00
5.97
11.0 10.5 10.0 9.5 9.0 8.5 8.0 7.5 7.0 6.5 6.0 5.5 5.0 4.5 4.0 3.5 3.0 2.5 2.0 1.5 1.0 0.5 0.0
chemical shift (ppm)

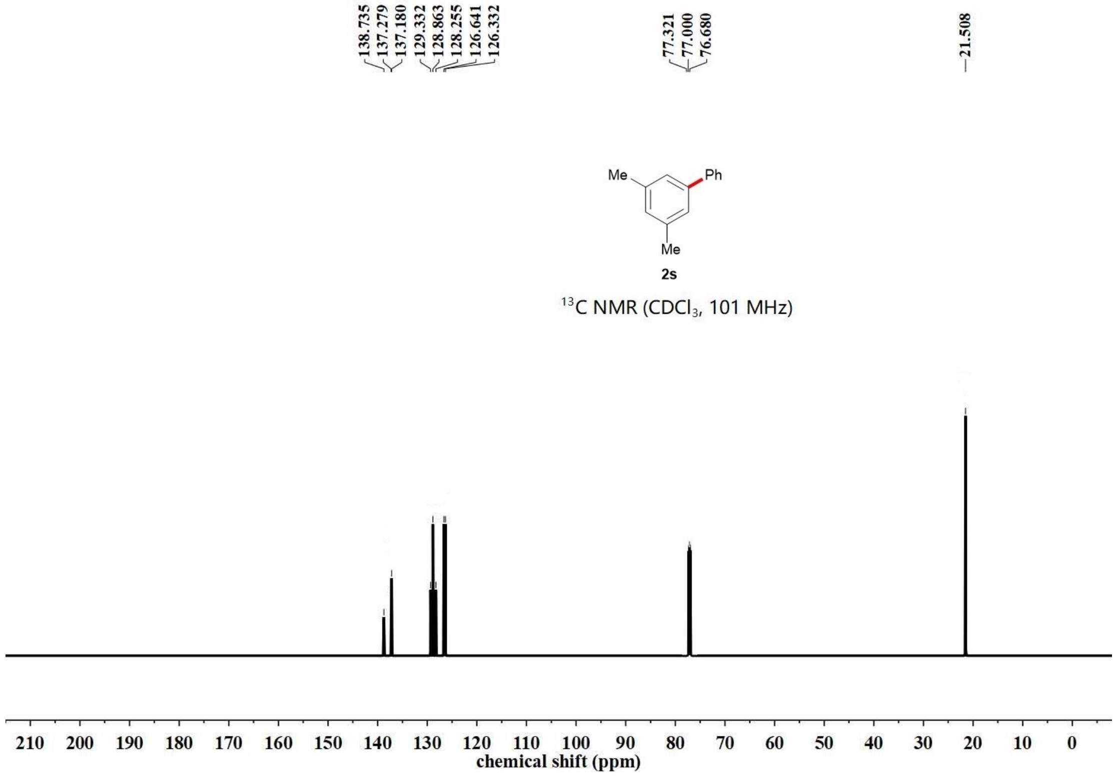
138.735 137.279 137.180 129.332 128.863 128.255 126.641 126.332
77.321 77.000 76.680
21.508
Me
Ph
Me
2s
^{13}C NMR (CDCl$_3$, 101 MHz)
210 200 190 180 170 160 150 140 130 120 110 100 90 80 70 60 50 40 30 20 10 0
chemical shift (ppm)

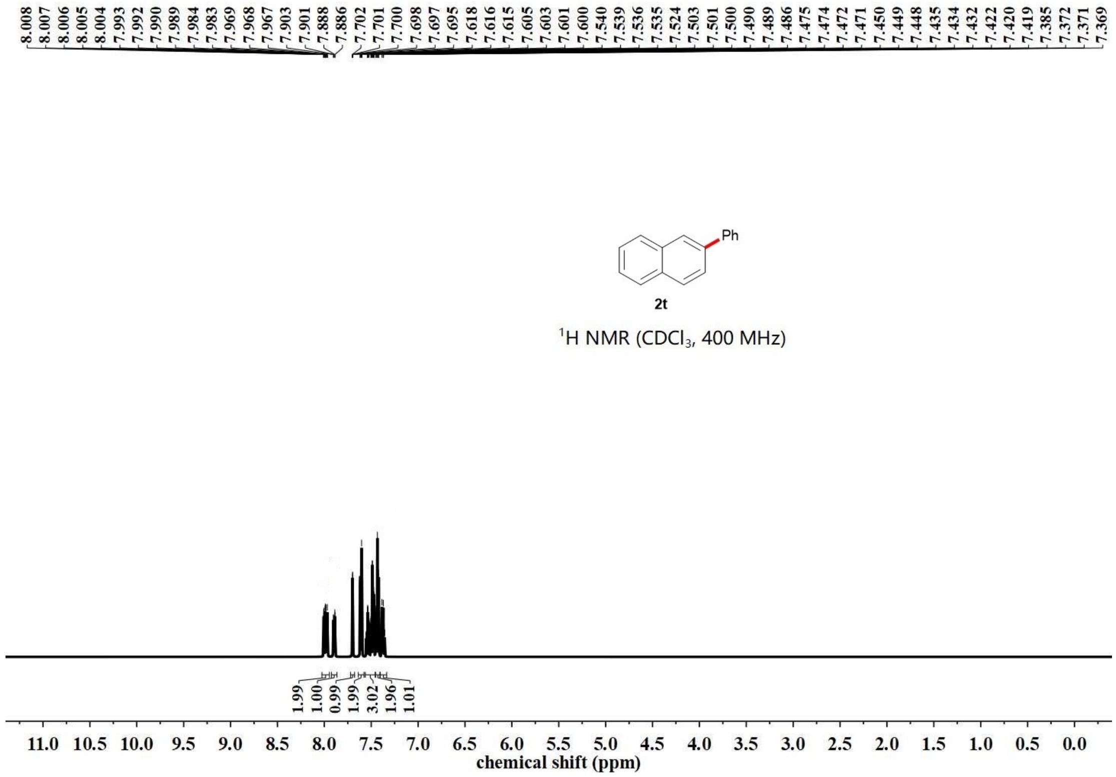

^{1}H NMR ($CDCl_3$, 400 MHz)

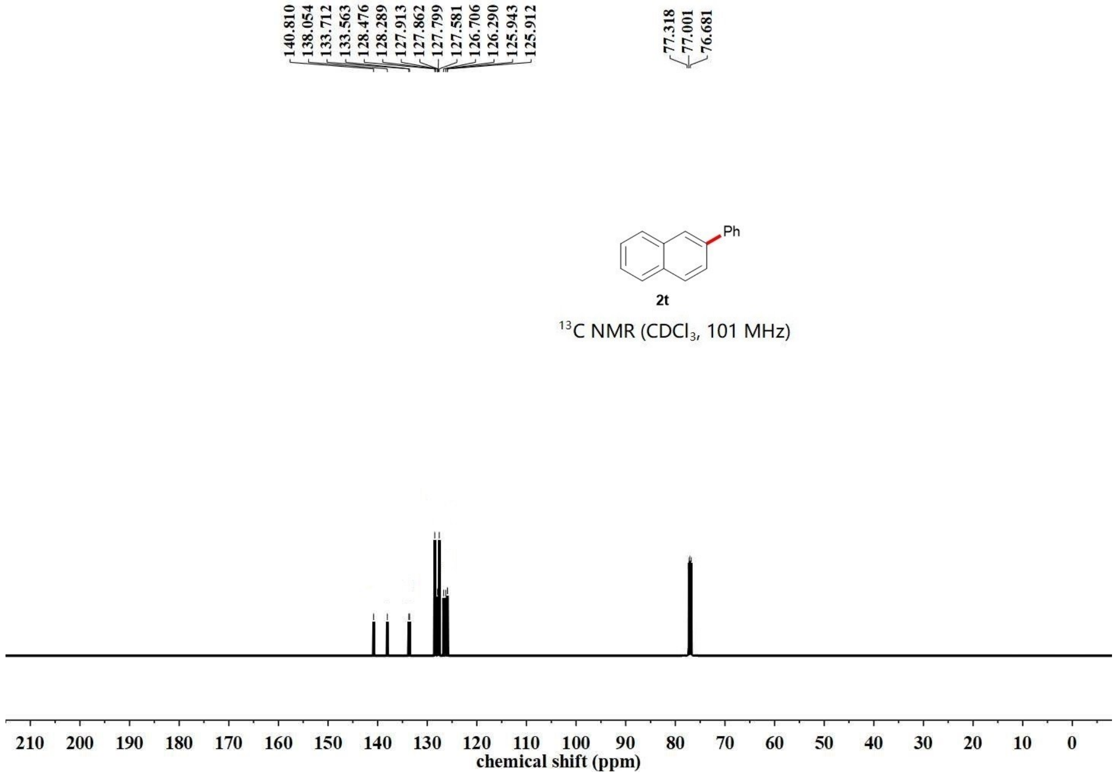

^{13}C NMR ($CDCl_3$, 101 MHz)

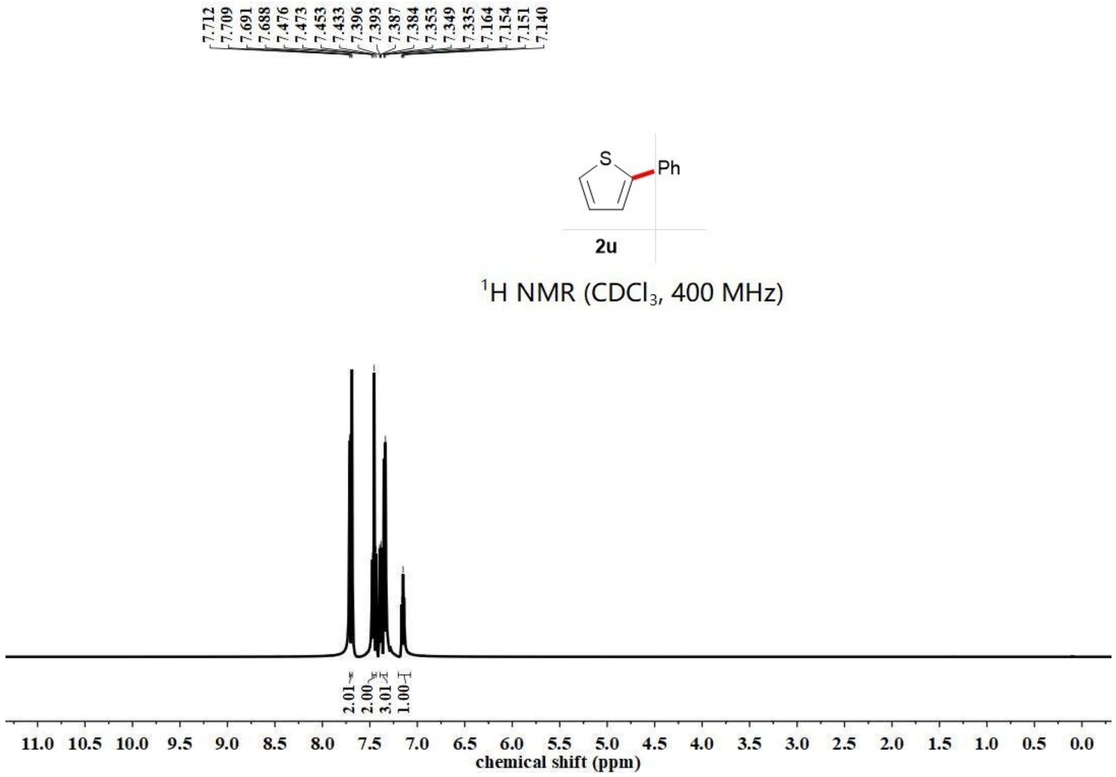
7.712
7.709
7.691
7.688
7.476
7.473
7.453
7.433
7.396
7.393
7.387
7.384
7.353
7.349
7.335
7.164
7.154
7.151
7.140
S
Ph
2u
^{1}H NMR ($CDCl_3$, 400 MHz)
2.01
2.00
3.01
1.00
11.0 10.5 10.0 9.5 9.0 8.5 8.0 7.5 7.0 6.5 6.0 5.5 5.0 4.5 4.0 3.5 3.0 2.5 2.0 1.5 1.0 0.5 0.0
chemical shift (ppm)

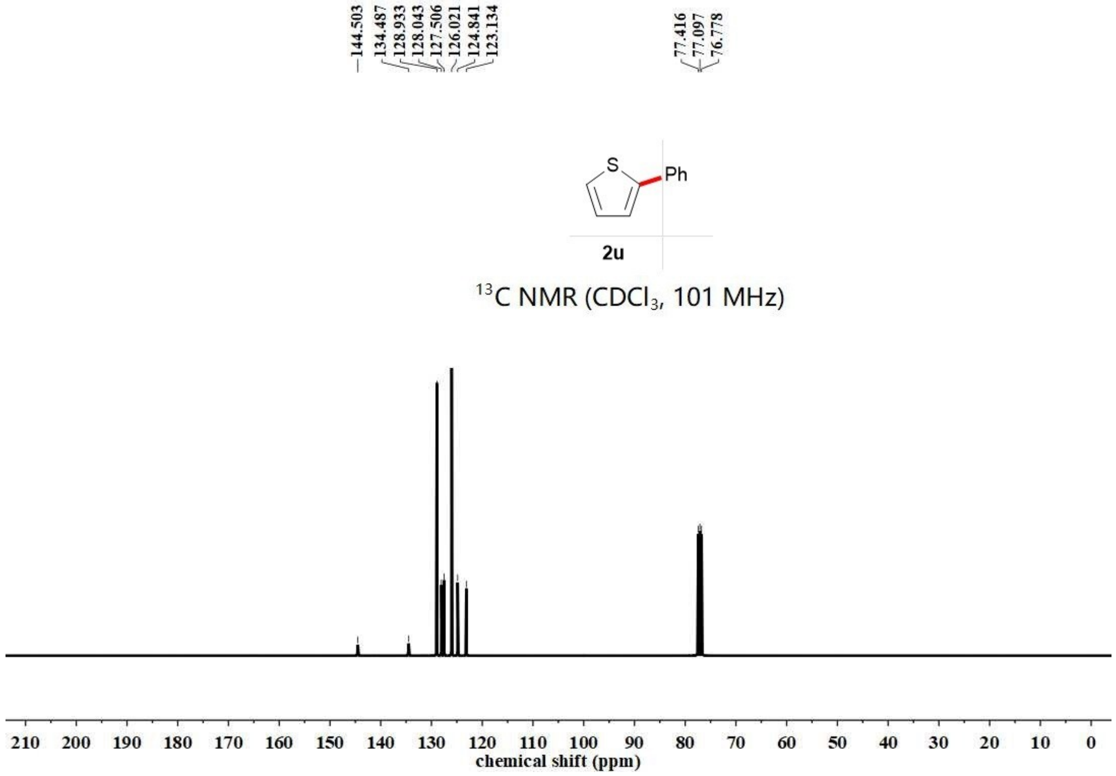
144.503
134.487
128.933
128.043
127.506
126.021
124.841
123.134
77.416
77.097
76.778
S
Ph
2u
^{13}C NMR ($CDCl_3$, 101 MHz)
210 200 190 180 170 160 150 140 130 120 110 100 90 80 70 60 50 40 30 20 10 0
chemical shift (ppm)

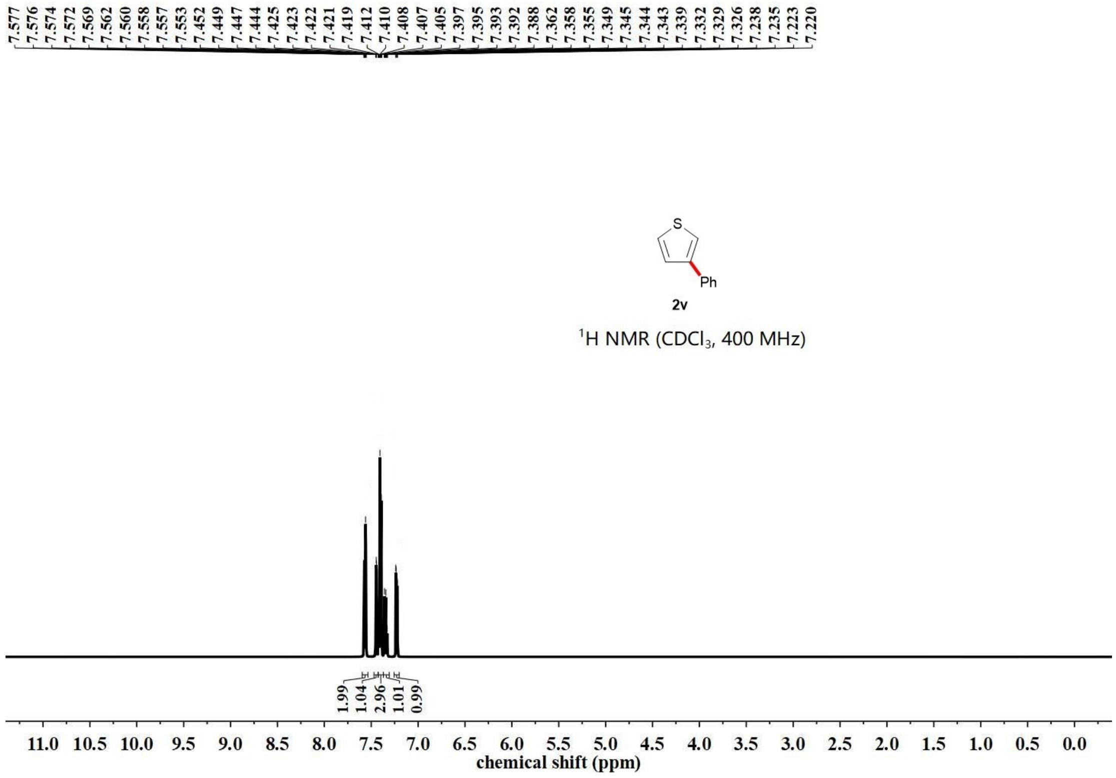
7.577 7.576 7.574 7.572 7.569 7.562 7.560 7.558 7.557 7.553 7.452 7.449 7.447 7.444 7.425 7.423 7.422 7.421 7.419 7.412 7.410 7.408 7.407 7.405 7.397 7.395 7.393 7.392 7.388 7.362 7.358 7.355 7.349 7.345 7.344 7.343 7.339 7.332 7.329 7.326 7.238 7.235 7.223 7.220
S
Ph
2v
^{1}H NMR (CDCl$_3$, 400 MHz)
1.99 1.04 2.96 1.01 0.99
11.0 10.5 10.0 9.5 9.0 8.5 8.0 7.5 7.0 6.5 6.0 5.5 5.0 4.5 4.0 3.5 3.0 2.5 2.0 1.5 1.0 0.5 0.0
chemical shift (ppm)

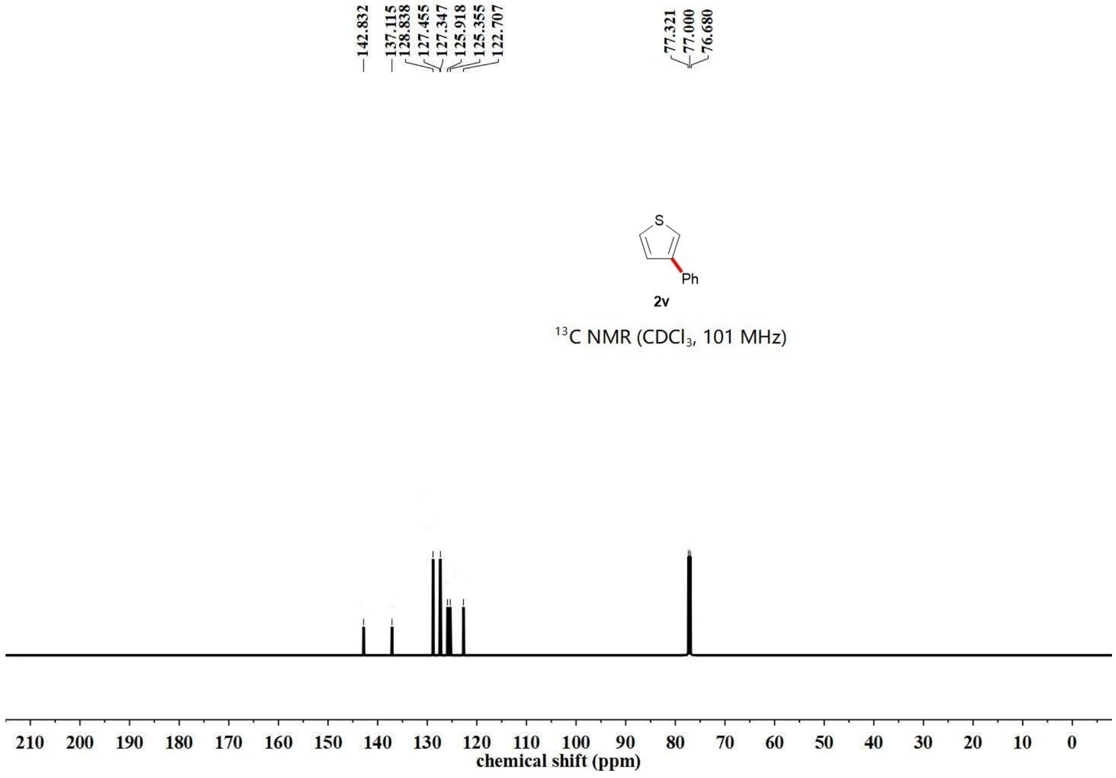
142.832 137.115 128.838 127.455 127.347 125.918 125.355 122.707
77.321 77.000 76.680
S
Ph
2v
^{13}C NMR (CDCl$_3$, 101 MHz)
210 200 190 180 170 160 150 140 130 120 110 100 90 80 70 60 50 40 30 20 10 0
chemical shift (ppm)

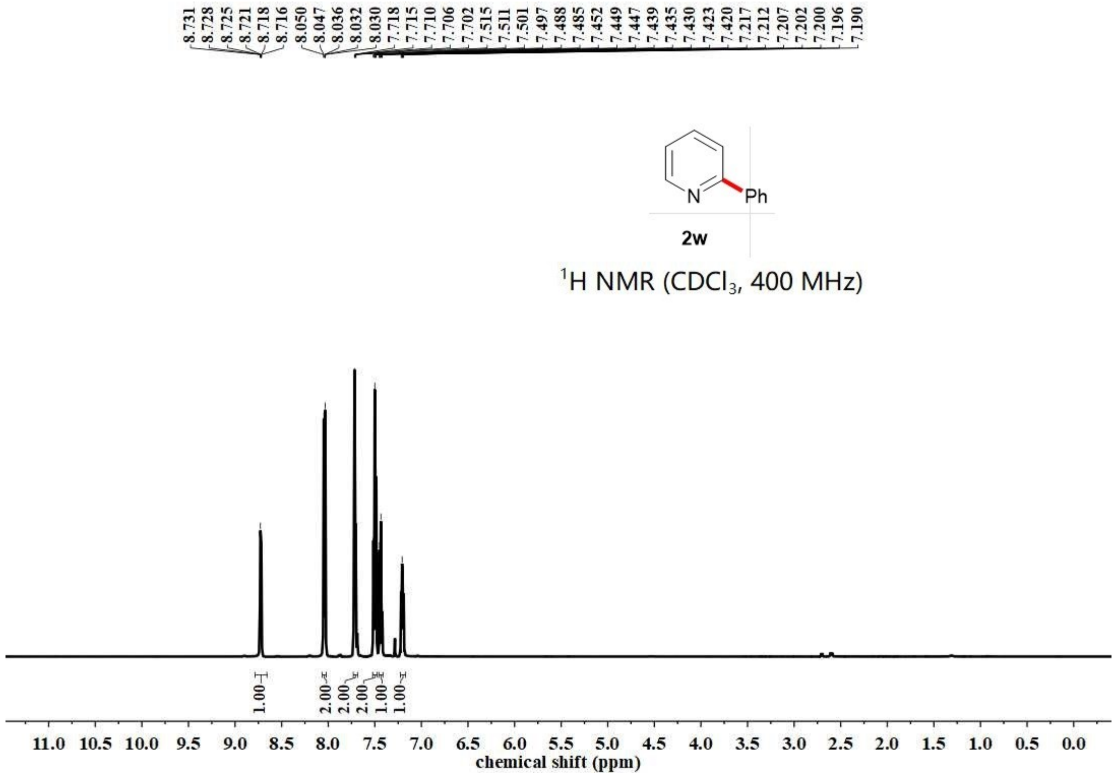
8.731
8.728
8.725
8.721
8.718
8.716
8.050
8.047
8.036
8.032
8.030
7.718
7.715
7.710
7.706
7.702
7.515
7.511
7.501
7.497
7.488
7.485
7.452
7.449
7.447
7.439
7.435
7.430
7.423
7.420
7.217
7.212
7.207
7.202
7.200
7.196
7.190
N
Ph
2w
1H NMR (CDCl3, 400 MHz)
1.00
2.00
2.00
2.00
1.00
1.00
11.0 10.5 10.0 9.5 9.0 8.5 8.0 7.5 7.0 6.5 6.0 5.5 5.0 4.5 4.0 3.5 3.0 2.5 2.0 1.5 1.0 0.5 0.0
chemical shift (ppm)

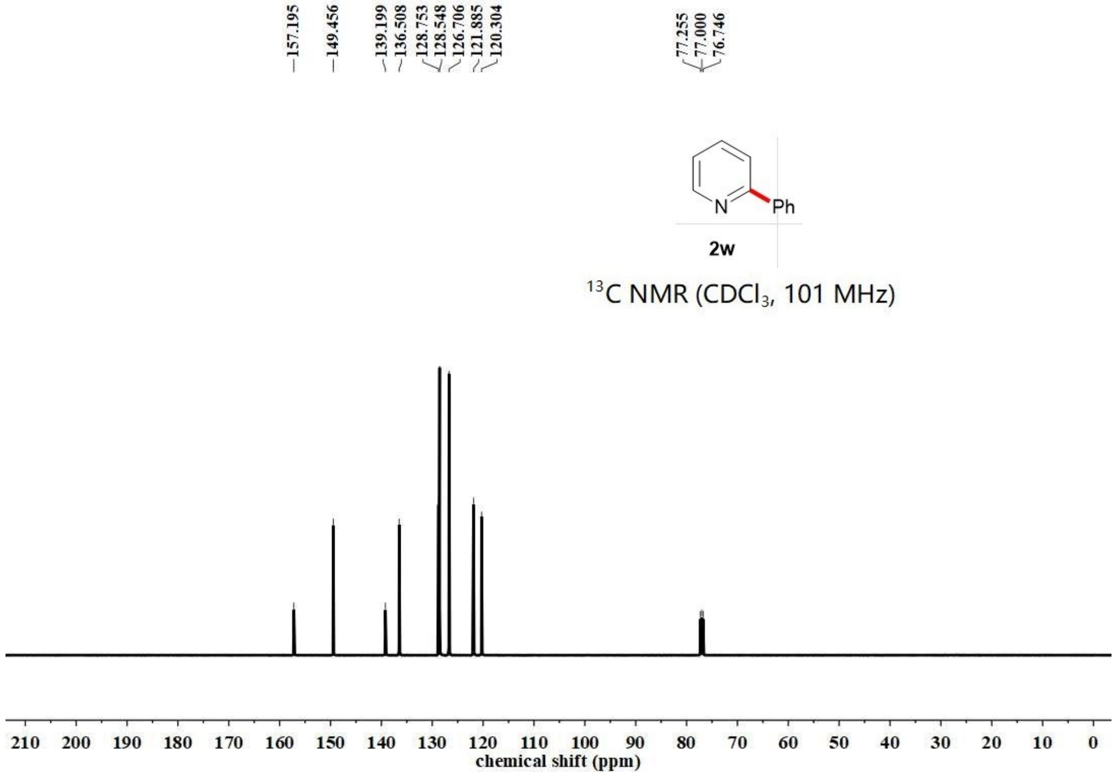
157.195
149.456
139.199
136.508
128.753
128.548
126.706
121.885
120.304
77.255
77.000
76.746
N
Ph
2w
13C NMR (CDCl3, 101 MHz)
210 200 190 180 170 160 150 140 130 120 110 100 90 80 70 60 50 40 30 20 10 0
chemical shift (ppm)

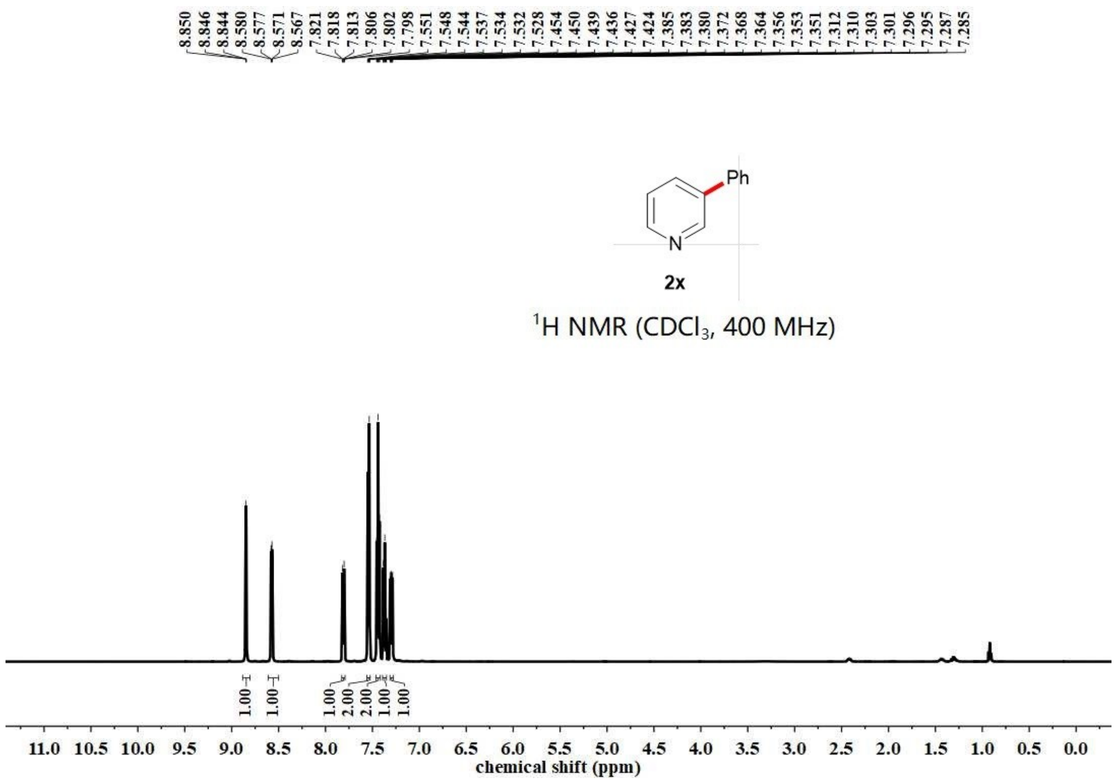
8.850
8.846
8.844
8.580
8.577
8.571
8.567
7.821
7.818
7.813
7.806
7.802
7.798
7.551
7.548
7.544
7.537
7.534
7.532
7.528
7.454
7.450
7.439
7.436
7.427
7.424
7.385
7.383
7.380
7.372
7.368
7.364
7.356
7.353
7.351
7.312
7.310
7.303
7.301
7.296
7.295
7.287
7.285
Ph
N
2x
1H NMR (CDCl3, 400 MHz)
1.00
1.00
1.00
2.00
2.00
1.00
1.00
chemical shift (ppm)

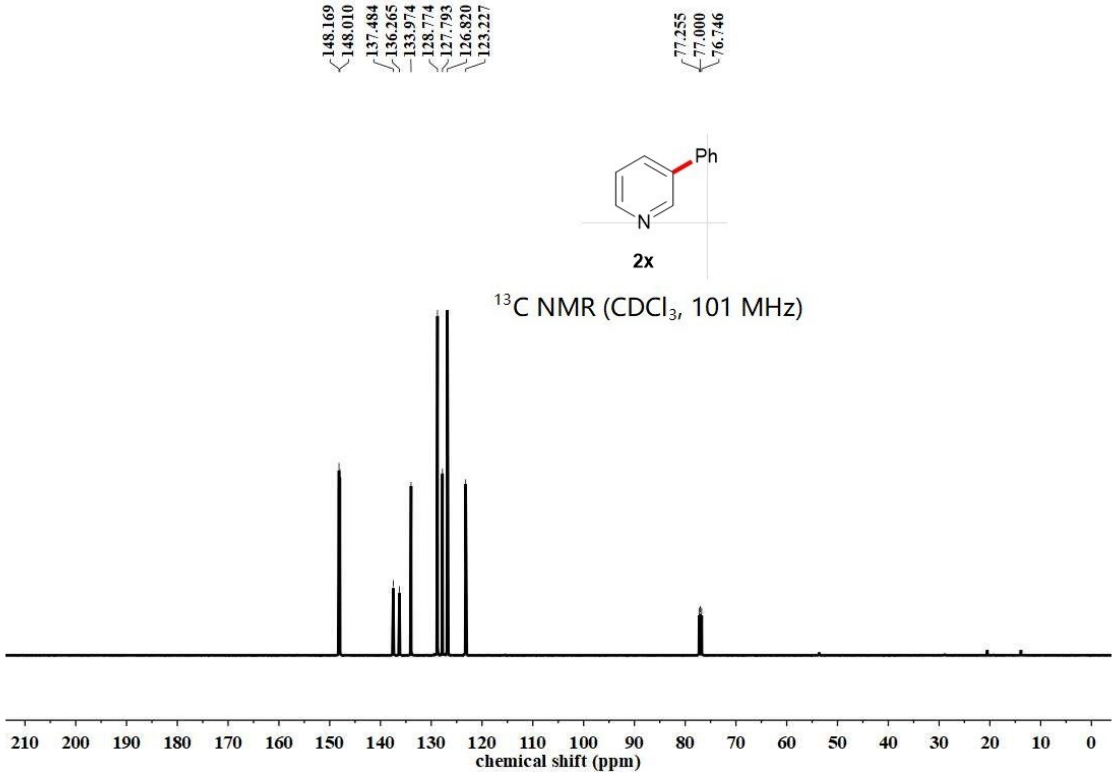
148.169
148.010
137.484
136.265
133.974
128.774
127.793
126.820
123.227
77.255
77.000
76.746
Ph
N
2x
13C NMR (CDCl3, 101 MHz)
chemical shift (ppm)

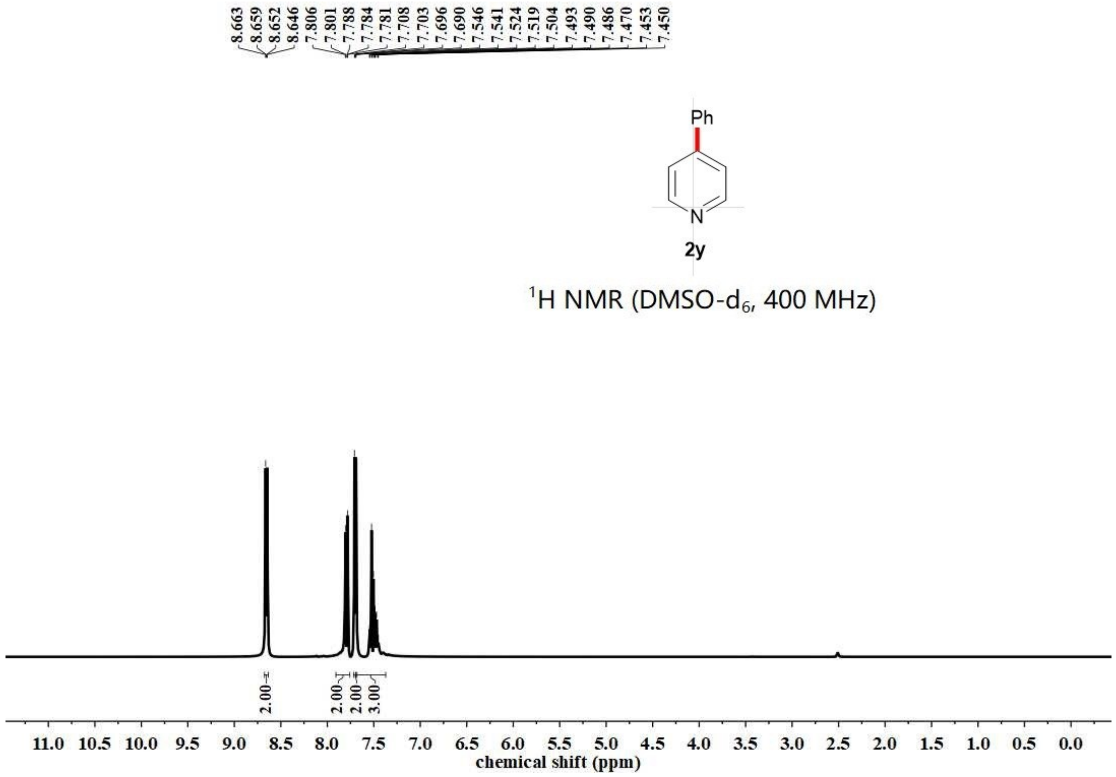

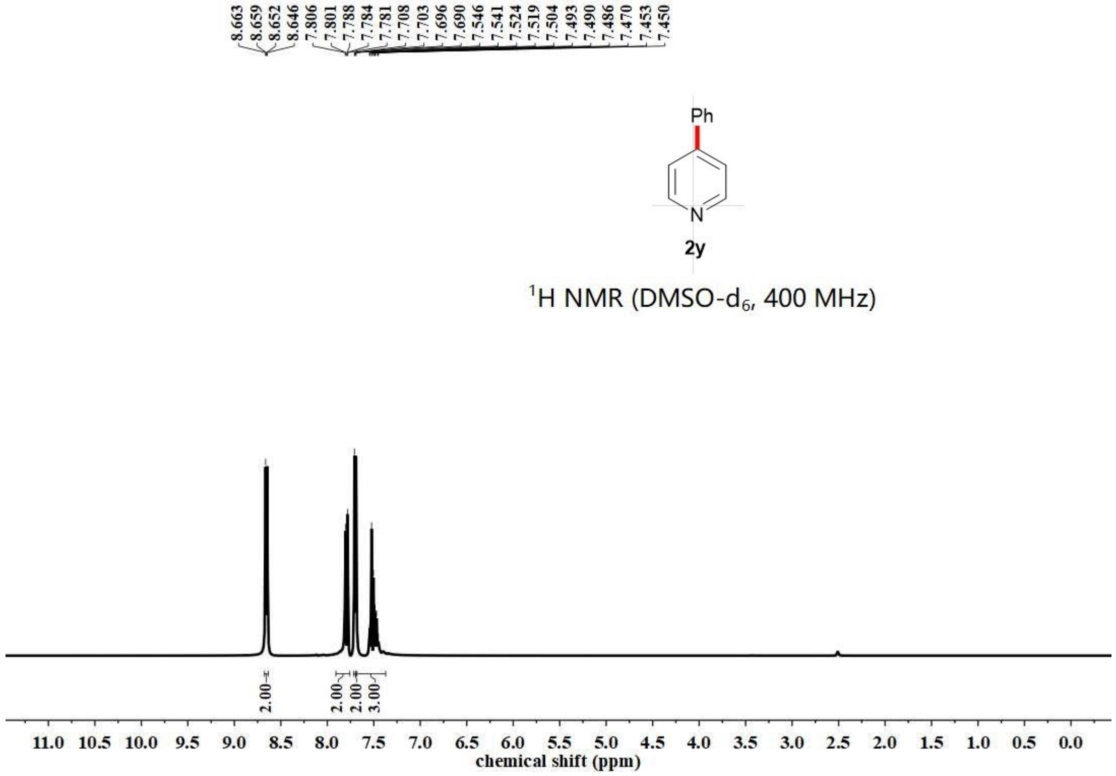
8.663 8.659 8.652 8.646 7.806 7.801 7.788 7.784 7.781 7.708 7.703 7.696 7.690 7.546 7.541 7.524 7.519 7.504 7.493 7.490 7.486 7.470 7.453 7.450
Ph
N
2y
^{1}H NMR (DMSO-d$_6$, 400 MHz)
2.00 2.00 2.00 3.00
11.0 10.5 10.0 9.5 9.0 8.5 8.0 7.5 7.0 6.5 6.0 5.5 5.0 4.5 4.0 3.5 3.0 2.5 2.0 1.5 1.0 0.5 0.0
chemical shift (ppm)

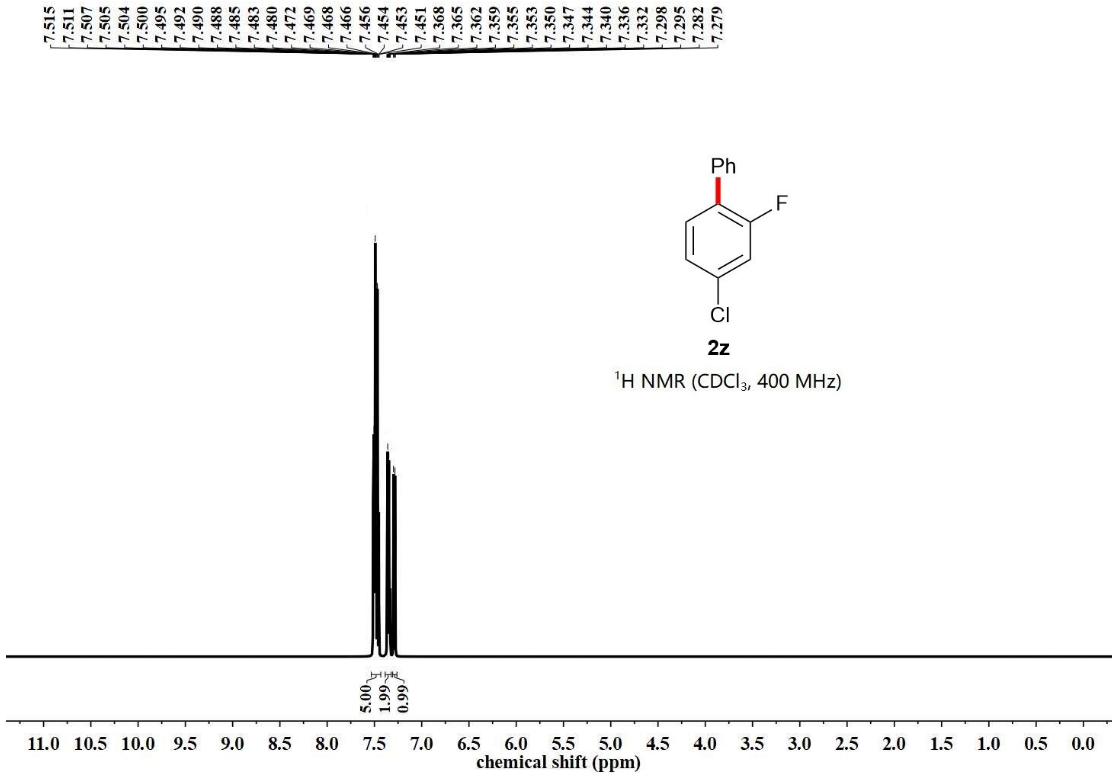

7.515 7.511 7.507 7.505 7.504 7.500 7.495 7.492 7.490 7.488 7.485 7.483 7.480 7.472 7.469 7.468 7.466 7.456 7.454 7.453 7.451 7.368 7.365 7.362 7.359 7.355 7.353 7.350 7.347 7.344 7.340 7.336 7.332 7.298 7.295 7.282 7.279
Ph
F
Cl
2z
1H NMR (CDCl3, 400 MHz)
5.00
1.99
0.99
11.0 10.5 10.0 9.5 9.0 8.5 8.0 7.5 7.0 6.5 6.0 5.5 5.0 4.5 4.0 3.5 3.0 2.5 2.0 1.5 1.0 0.5 0.0
chemical shift (ppm)

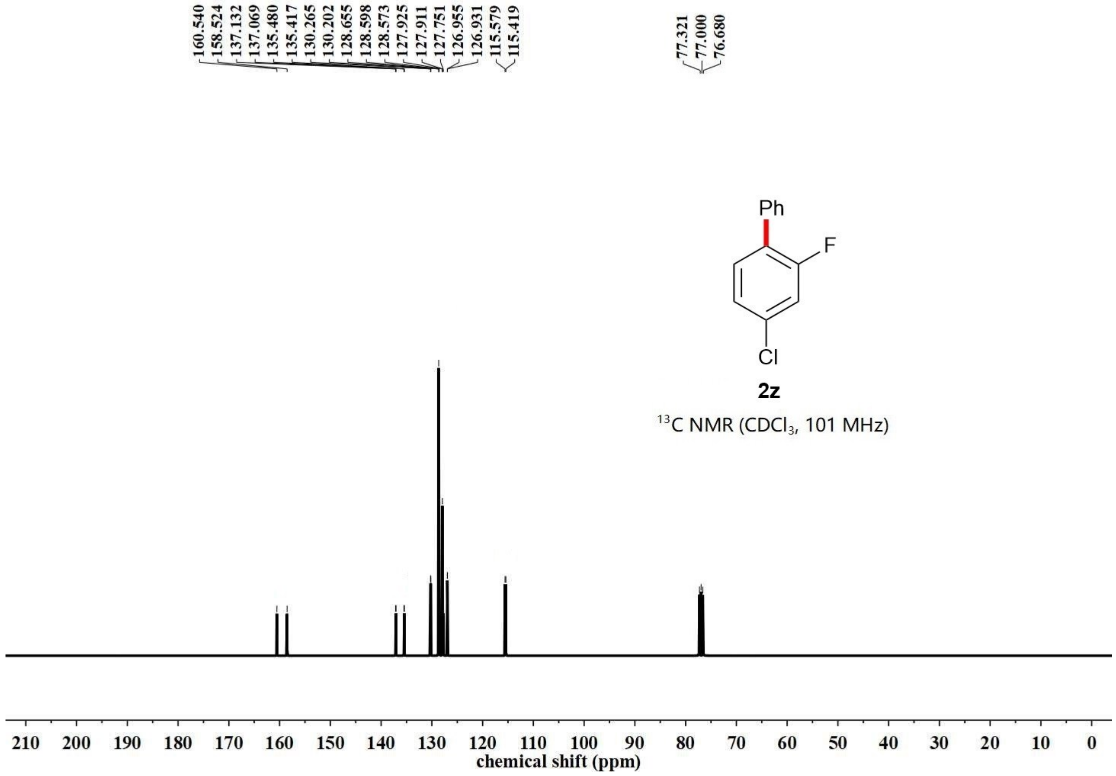

160.540 158.524 137.132 137.069 135.480 135.417 130.265 130.202 128.655 128.598 128.573 127.925 127.911 127.751 126.955 126.931 115.579 115.419
77.321 77.000 76.680
Ph
F
Cl
2z
13C NMR (CDCl3, 101 MHz)
210 200 190 180 170 160 150 140 130 120 110 100 90 80 70 60 50 40 30 20 10 0
chemical shift (ppm)

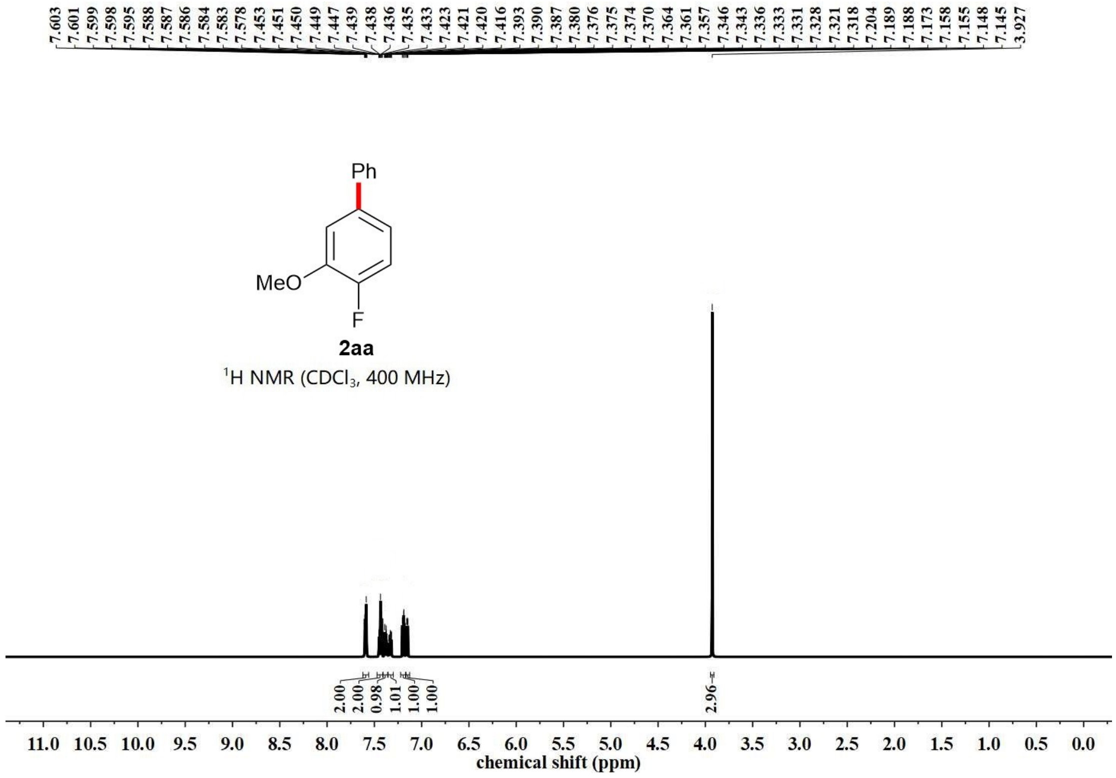
7.603
7.601
7.599
7.598
7.595
7.588
7.587
7.586
7.584
7.583
7.578
7.453
7.451
7.450
7.449
7.447
7.439
7.438
7.436
7.435
7.433
7.423
7.421
7.420
7.416
7.393
7.390
7.387
7.380
7.376
7.375
7.374
7.370
7.364
7.361
7.357
7.346
7.343
7.336
7.333
7.331
7.328
7.321
7.318
7.204
7.189
7.188
7.173
7.158
7.155
7.148
7.145
3.927
Ph
MeO
F
2aa
^{1}H NMR (CDCl$_3$, 400 MHz)
2.00
2.00
0.98
1.01
1.00
1.00
2.96
11.0 10.5 10.0 9.5 9.0 8.5 8.0 7.5 7.0 6.5 6.0 5.5 5.0 4.5 4.0 3.5 3.0 2.5 2.0 1.5 1.0 0.5 0.0
chemical shift (ppm)

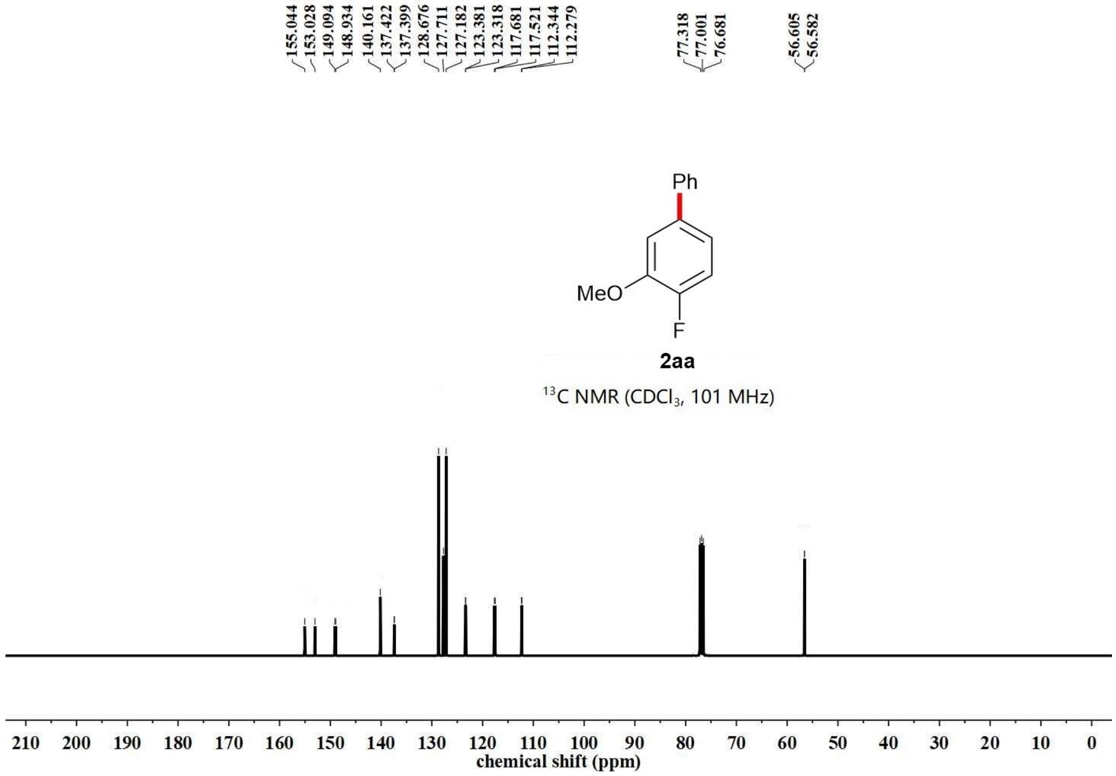
155.044
153.028
149.094
148.934
140.161
137.422
137.399
128.676
127.711
127.182
123.381
123.318
117.681
117.521
112.344
112.279
77.318
77.001
76.681
56.605
56.582
Ph
MeO
F
2aa
^{13}C NMR (CDCl$_3$, 101 MHz)
210 200 190 180 170 160 150 140 130 120 110 100 90 80 70 60 50 40 30 20 10 0
chemical shift (ppm)

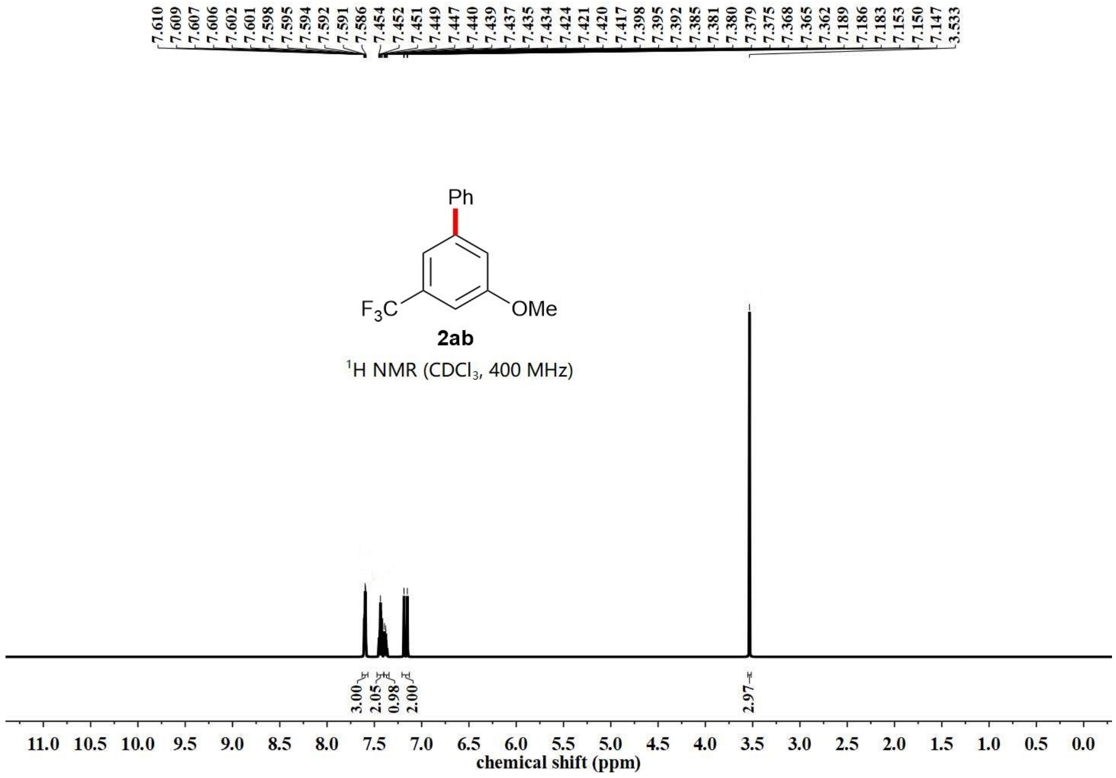

7.610
7.609
7.607
7.606
7.602
7.601
7.598
7.595
7.594
7.592
7.591
7.586
7.454
7.452
7.451
7.449
7.447
7.440
7.439
7.437
7.435
7.434
7.424
7.421
7.420
7.417
7.398
7.395
7.392
7.385
7.381
7.380
7.379
7.375
7.368
7.365
7.362
7.189
7.186
7.183
7.153
7.150
7.147
3.533
Ph
F3C
OMe
2ab
1H NMR (CDCl3, 400 MHz)
3.00
2.05
0.98
2.00
2.97
11.0 10.5 10.0 9.5 9.0 8.5 8.0 7.5 7.0 6.5 6.0 5.5 5.0 4.5 4.0 3.5 3.0 2.5 2.0 1.5 1.0 0.5 0.0
chemical shift (ppm)

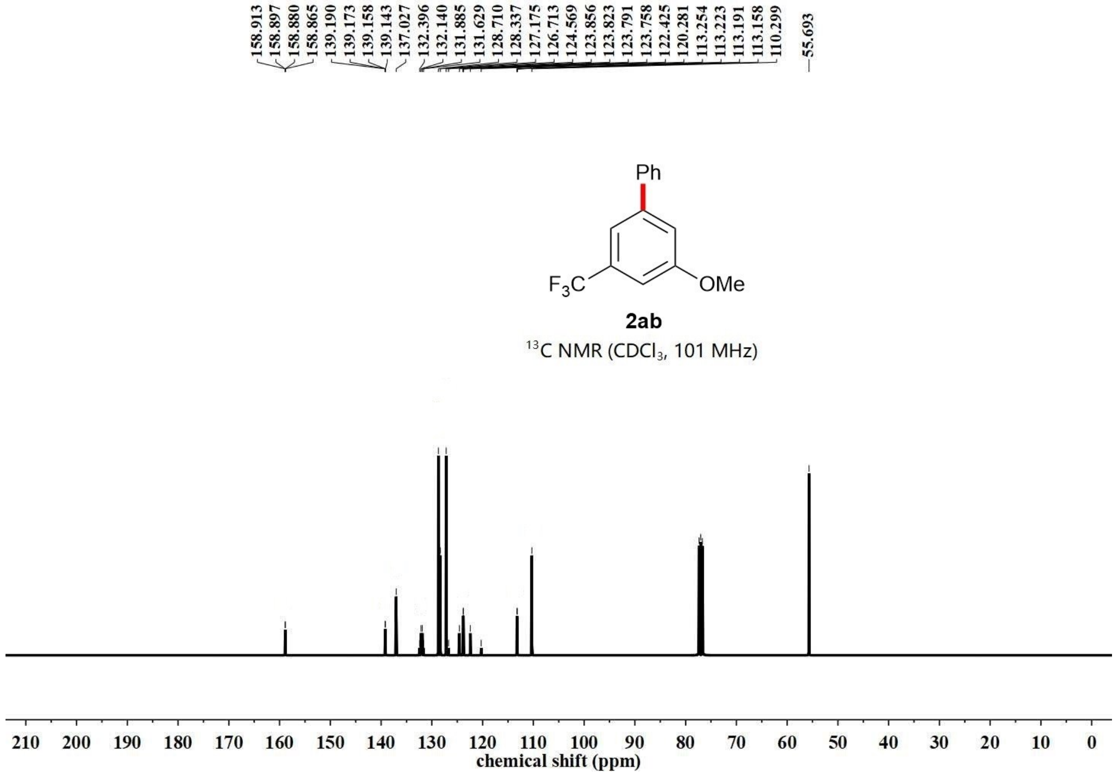

158.913
158.897
158.880
158.865
139.190
139.173
139.158
139.143
137.027
132.396
132.140
131.885
131.629
128.710
128.337
127.175
126.713
124.569
123.856
123.823
123.791
123.758
122.425
120.281
113.254
113.223
113.191
113.158
110.299
55.693
Ph
F3C
OMe
2ab
13C NMR (CDCl3, 101 MHz)
210 200 190 180 170 160 150 140 130 120 110 100 90 80 70 60 50 40 30 20 10 0
chemical shift (ppm)

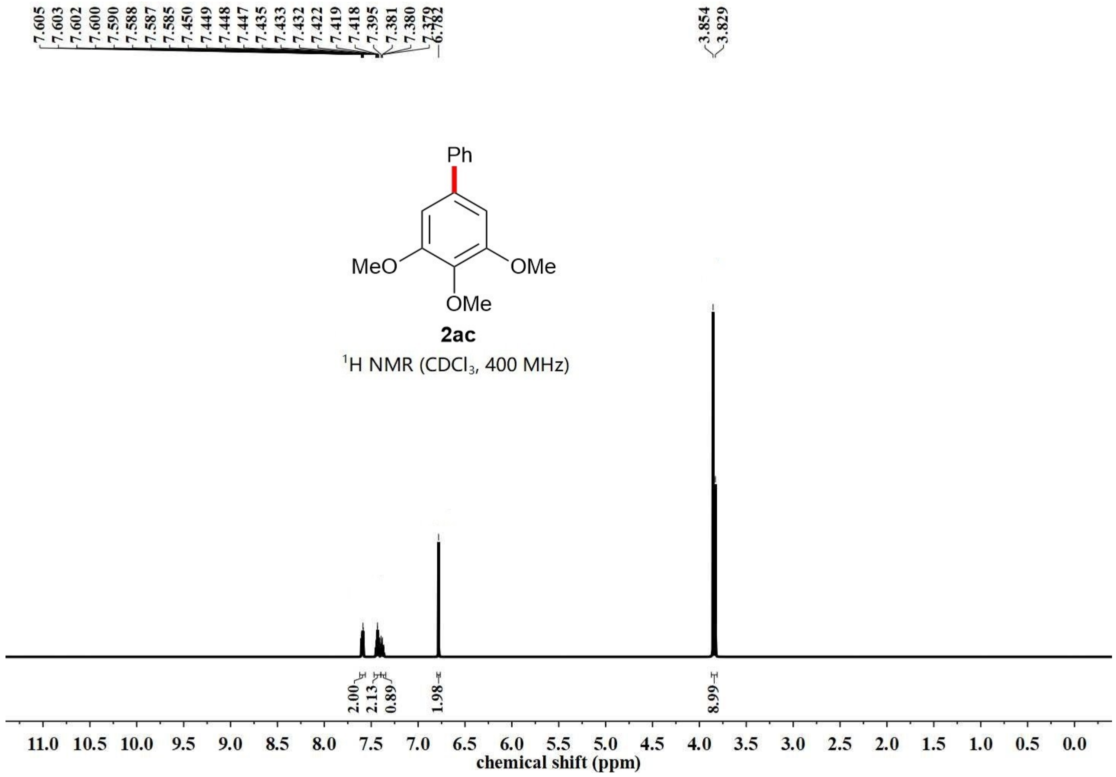
7.605
7.603
7.602
7.600
7.590
7.588
7.587
7.585
7.450
7.449
7.448
7.447
7.435
7.433
7.432
7.422
7.419
7.418
7.395
7.381
7.380
7.379
6.782
3.854
3.829
Ph
MeO
OMe
OMe
2ac
^{1}H NMR ($CDCl_3$, 400 MHz)
2.00
2.13
0.89
1.98
8.99
11.0 10.5 10.0 9.5 9.0 8.5 8.0 7.5 7.0 6.5 6.0 5.5 5.0 4.5 4.0 3.5 3.0 2.5 2.0 1.5 1.0 0.5 0.0
chemical shift (ppm)

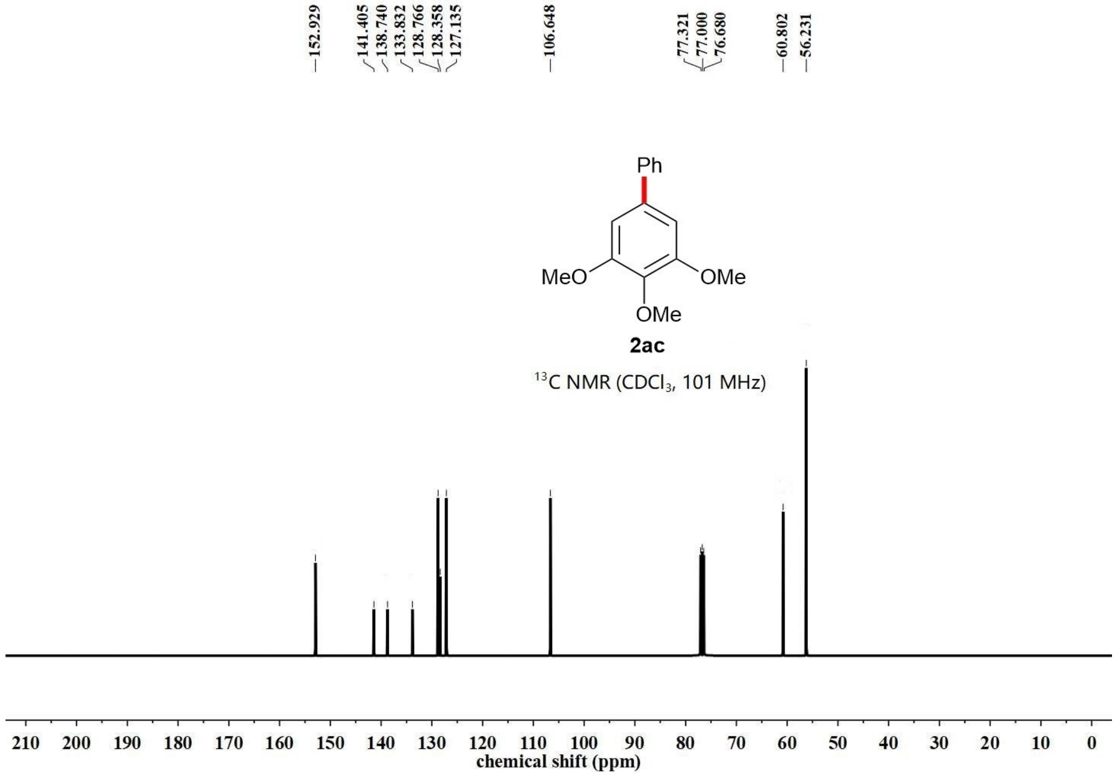
152.929
141.405
138.740
133.832
128.766
128.358
127.135
106.648
77.321
77.000
76.680
60.802
56.231
Ph
MeO
OMe
OMe
2ac
^{13}C NMR ($CDCl_3$, 101 MHz)
210 200 190 180 170 160 150 140 130 120 110 100 90 80 70 60 50 40 30 20 10 0
chemical shift (ppm)

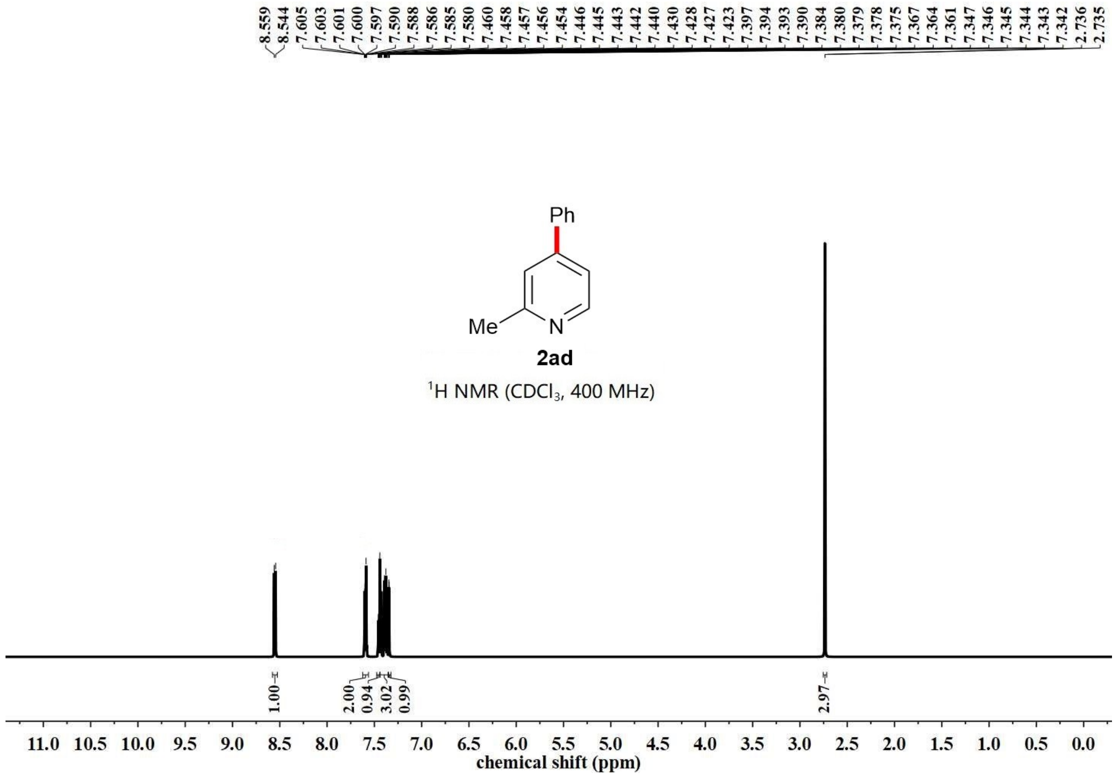
8.559
8.544
7.605
7.603
7.601
7.600
7.597
7.590
7.588
7.586
7.585
7.580
7.460
7.458
7.457
7.456
7.454
7.446
7.445
7.443
7.442
7.440
7.430
7.428
7.427
7.423
7.397
7.394
7.393
7.390
7.384
7.380
7.379
7.378
7.375
7.367
7.364
7.361
7.347
7.346
7.345
7.344
7.343
7.342
2.736
2.735
Ph
Me
N
2ad
^{1}H NMR (CDCl$_3$, 400 MHz)
1.00
2.00
0.94
3.02
0.99
2.97
11.0 10.5 10.0 9.5 9.0 8.5 8.0 7.5 7.0 6.5 6.0 5.5 5.0 4.5 4.0 3.5 3.0 2.5 2.0 1.5 1.0 0.5 0.0
chemical shift (ppm)

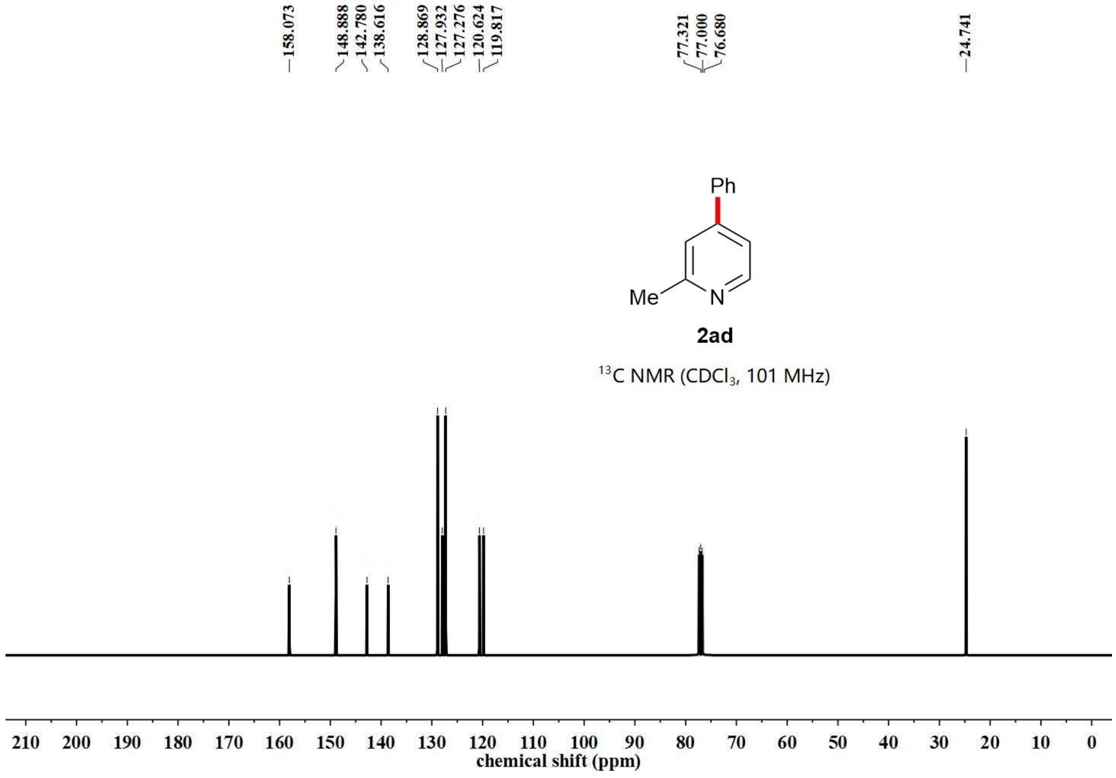
158.073
148.888
142.780
138.616
128.869
127.932
127.276
120.624
119.817
77.321
77.000
76.680
24.741
Ph
Me
N
2ad
^{13}C NMR (CDCl$_3$, 101 MHz)
210 200 190 180 170 160 150 140 130 120 110 100 90 80 70 60 50 40 30 20 10 0
chemical shift (ppm)

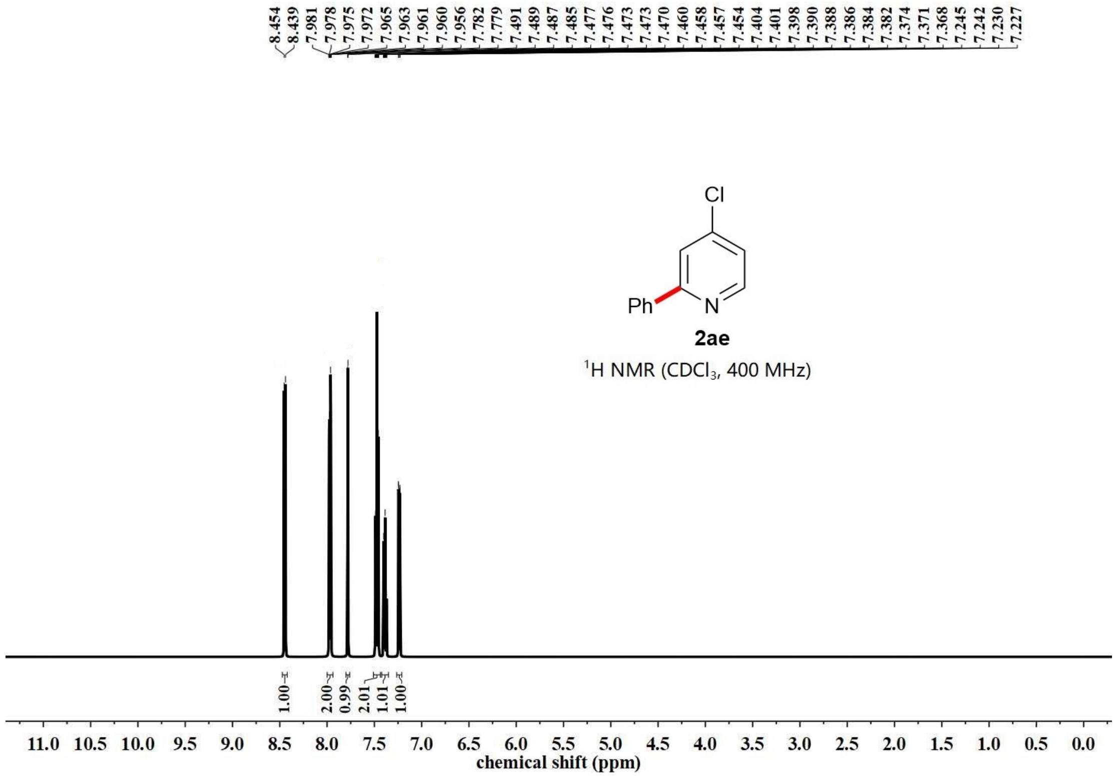
8.454
8.439
7.981
7.978
7.975
7.972
7.965
7.963
7.961
7.960
7.956
7.782
7.779
7.491
7.489
7.487
7.485
7.477
7.476
7.473
7.473
7.470
7.460
7.458
7.457
7.454
7.404
7.401
7.398
7.390
7.388
7.386
7.384
7.382
7.374
7.371
7.368
7.245
7.242
7.230
7.227
Cl
Ph
N
2ae
1H NMR (CDCl3, 400 MHz)
1.00
2.00
0.99
2.01
1.01
1.00
11.0 10.5 10.0 9.5 9.0 8.5 8.0 7.5 7.0 6.5 6.0 5.5 5.0 4.5 4.0 3.5 3.0 2.5 2.0 1.5 1.0 0.5 0.0
chemical shift (ppm)

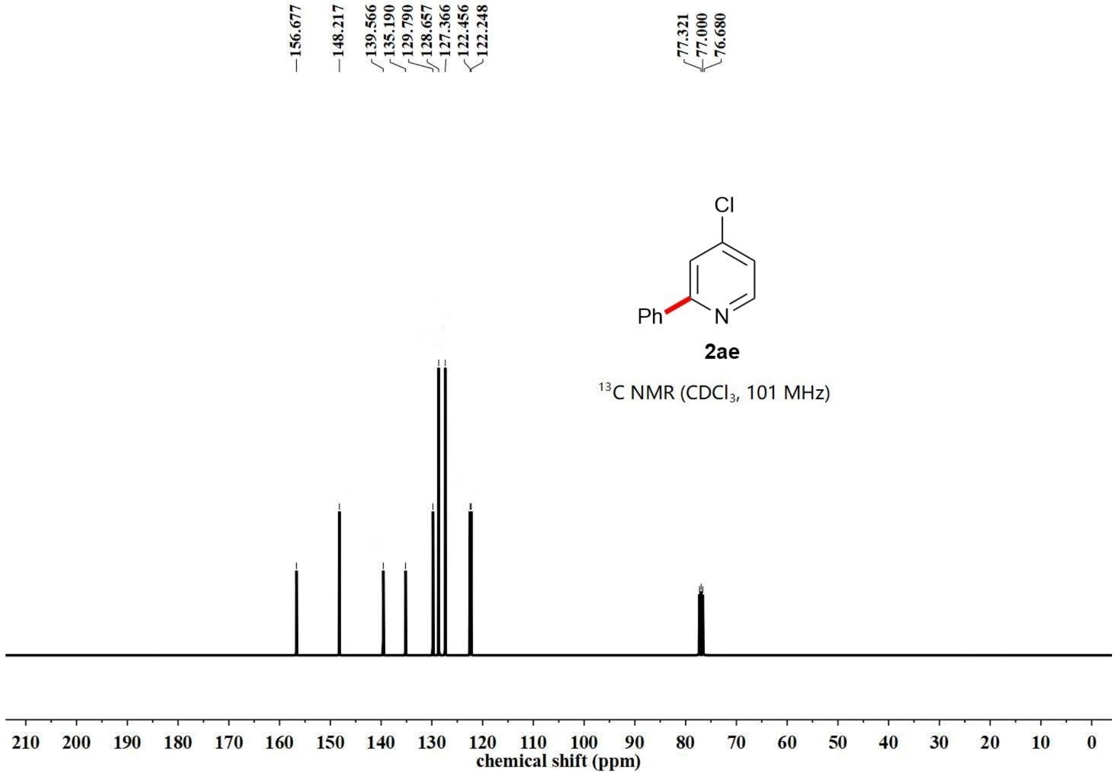
156.677
148.217
139.566
135.190
129.790
128.657
127.366
122.456
122.248
77.321
77.000
76.680
Cl
Ph
N
2ae
13C NMR (CDCl3, 101 MHz)
210 200 190 180 170 160 150 140 130 120 110 100 90 80 70 60 50 40 30 20 10 0
chemical shift (ppm)

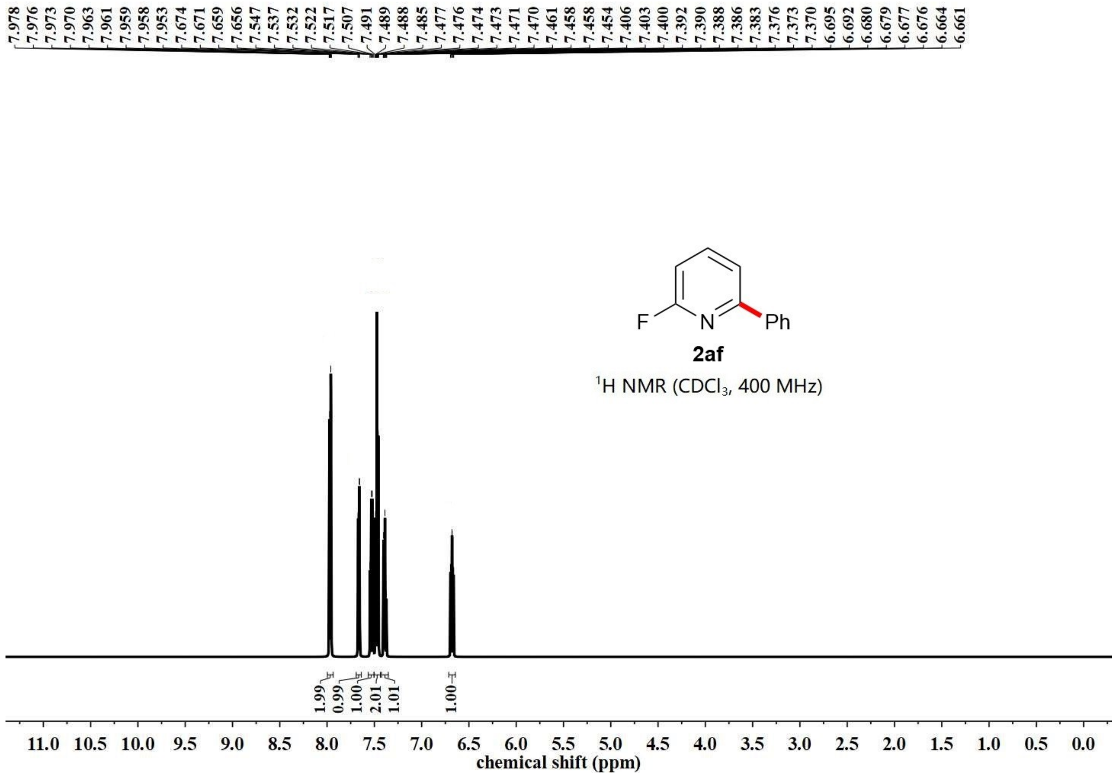
7.978 7.976 7.973 7.970 7.963 7.961 7.959 7.958 7.953 7.674 7.671 7.659 7.656 7.547 7.537 7.532 7.522 7.517 7.507 7.491 7.489 7.488 7.485 7.477 7.476 7.474 7.473 7.471 7.470 7.461 7.458 7.458 7.454 7.406 7.403 7.400 7.392 7.390 7.388 7.386 7.383 7.376 7.373 7.370 6.695 6.692 6.680 6.679 6.677 6.676 6.664 6.661
F
N
Ph
2af
1H NMR (CDCl3, 400 MHz)
1.99 0.99 1.00 2.01 1.01 1.00
11.0 10.5 10.0 9.5 9.0 8.5 8.0 7.5 7.0 6.5 6.0 5.5 5.0 4.5 4.0 3.5 3.0 2.5 2.0 1.5 1.0 0.5 0.0
chemical shift (ppm)

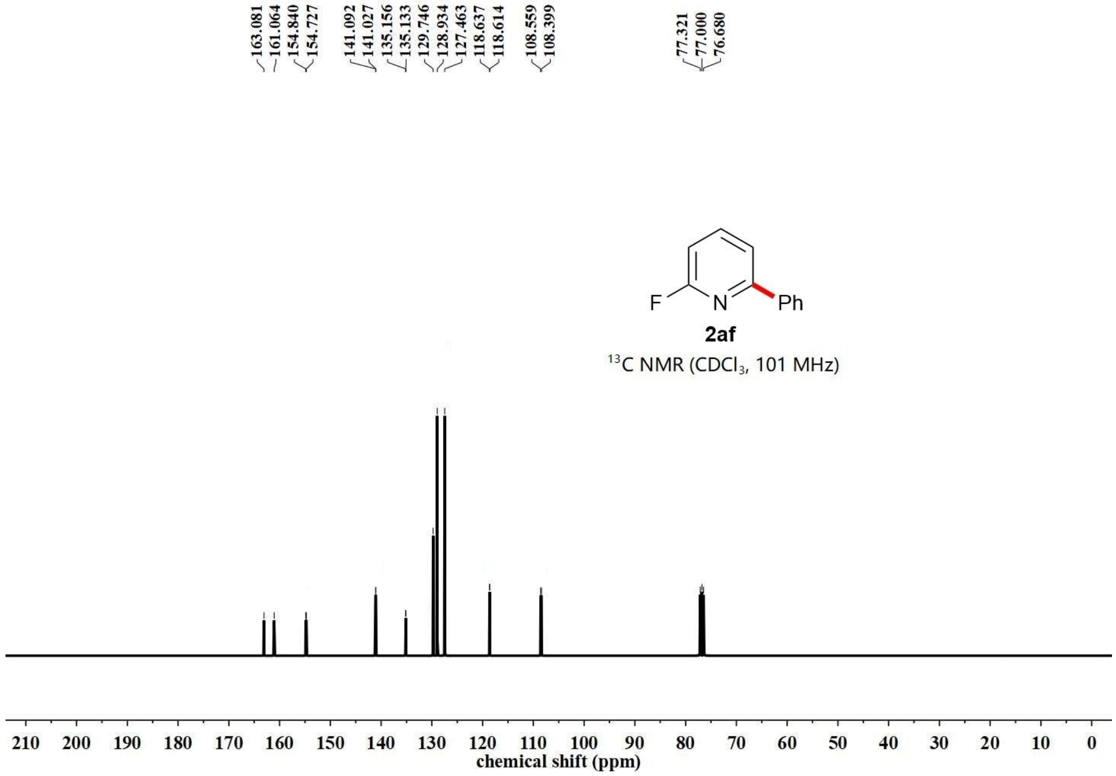
163.081 161.064 154.840 154.727 141.092 141.027 135.156 135.133 129.746 128.934 127.463 118.637 118.614 108.559 108.399 77.321 77.000 76.680
F
N
Ph
2af
13C NMR (CDCl3, 101 MHz)
210 200 190 180 170 160 150 140 130 120 110 100 90 80 70 60 50 40 30 20 10 0
chemical shift (ppm)

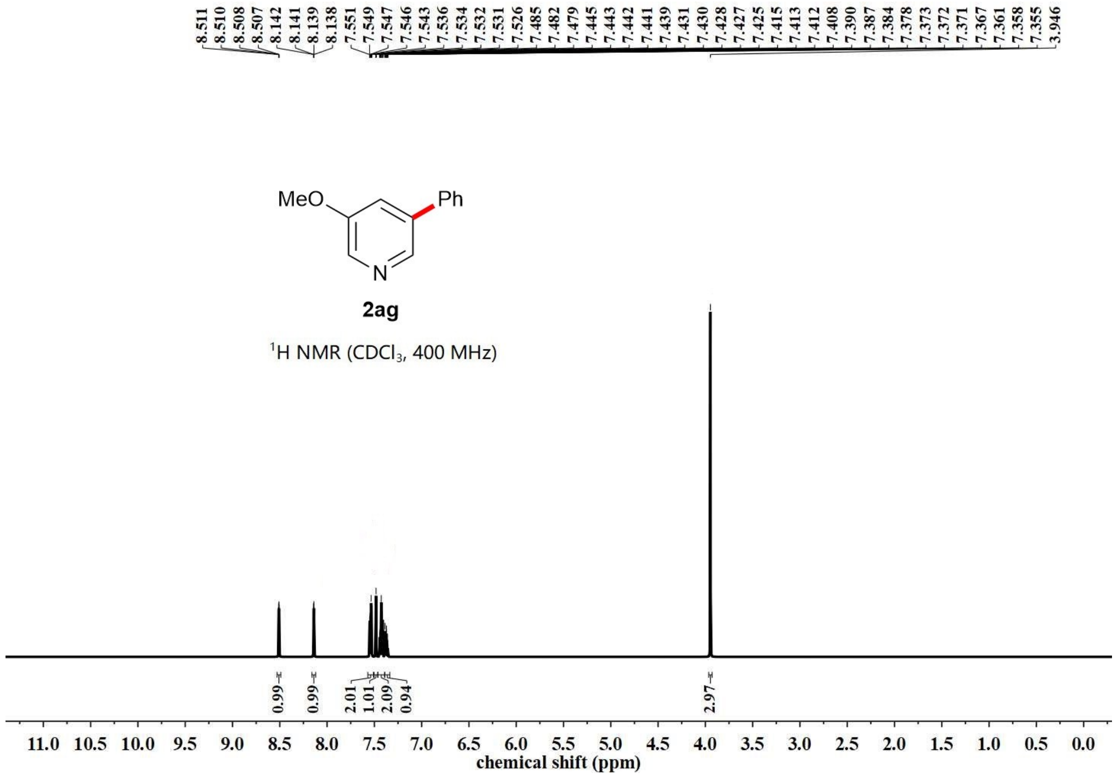
8.511
8.510
8.508
8.507
8.142
8.141
8.139
8.138
7.551
7.549
7.547
7.546
7.543
7.536
7.534
7.532
7.531
7.526
7.485
7.482
7.479
7.445
7.443
7.442
7.441
7.439
7.431
7.430
7.428
7.427
7.425
7.415
7.413
7.412
7.408
7.390
7.387
7.384
7.378
7.373
7.372
7.371
7.367
7.361
7.358
7.355
3.946
MeO
Ph
N
2ag
^{1}H NMR (CDCl$_3$, 400 MHz)
0.99
0.99
2.01
1.01
2.09
0.94
2.97
11.0 10.5 10.0 9.5 9.0 8.5 8.0 7.5 7.0 6.5 6.0 5.5 5.0 4.5 4.0 3.5 3.0 2.5 2.0 1.5 1.0 0.5 0.0
chemical shift (ppm)

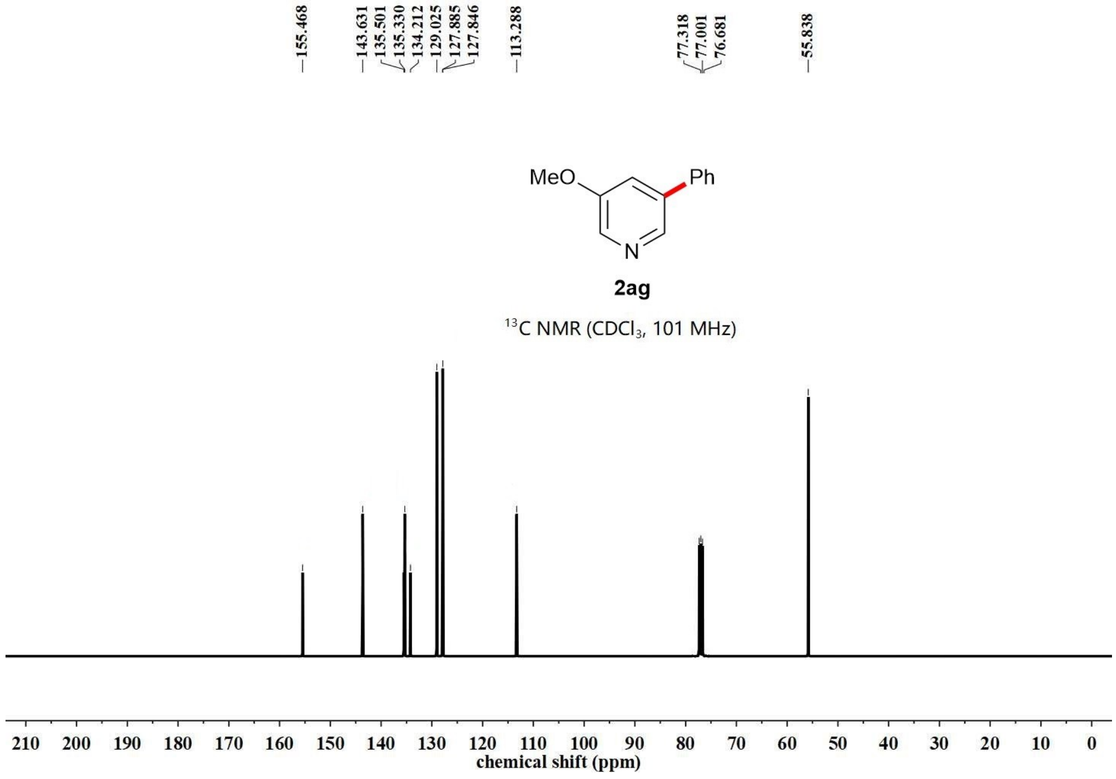
155.468
143.631
135.501
135.330
134.212
129.025
127.885
127.846
113.288
77.318
77.001
76.681
55.838
MeO
Ph
N
2ag
^{13}C NMR (CDCl$_3$, 101 MHz)
210 200 190 180 170 160 150 140 130 120 110 100 90 80 70 60 50 40 30 20 10 0
chemical shift (ppm)

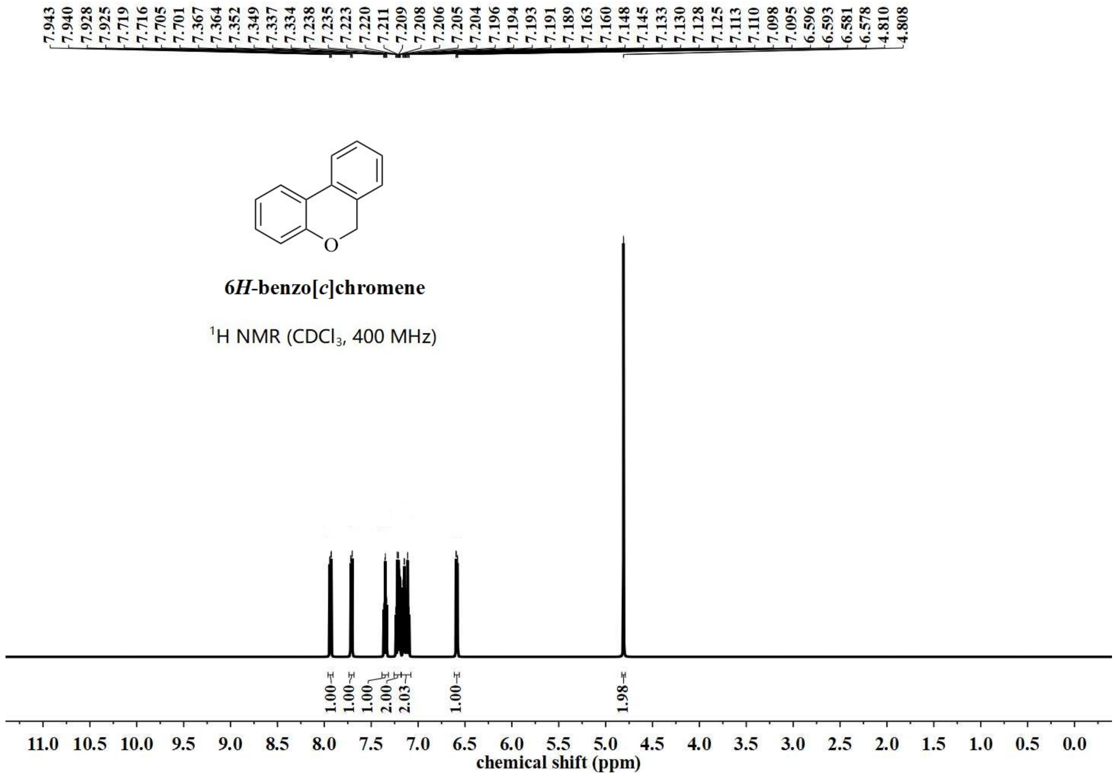
7.943 7.940 7.928 7.925 7.719 7.716 7.705 7.701 7.367 7.364 7.352 7.349 7.337 7.334 7.238 7.235 7.223 7.220 7.211 7.209 7.208 7.206 7.205 7.204 7.196 7.194 7.193 7.191 7.189 7.163 7.160 7.148 7.145 7.133 7.130 7.128 7.125 7.113 7.110 7.098 7.095 6.596 6.593 6.581 6.578 4.810 4.808
O
6H-benzo[c]chromene
1H NMR (CDCl3, 400 MHz)
1.00 1.00 1.00 2.00 2.03 1.00 1.98
11.0 10.5 10.0 9.5 9.0 8.5 8.0 7.5 7.0 6.5 6.0 5.5 5.0 4.5 4.0 3.5 3.0 2.5 2.0 1.5 1.0 0.5 0.0
chemical shift (ppm)

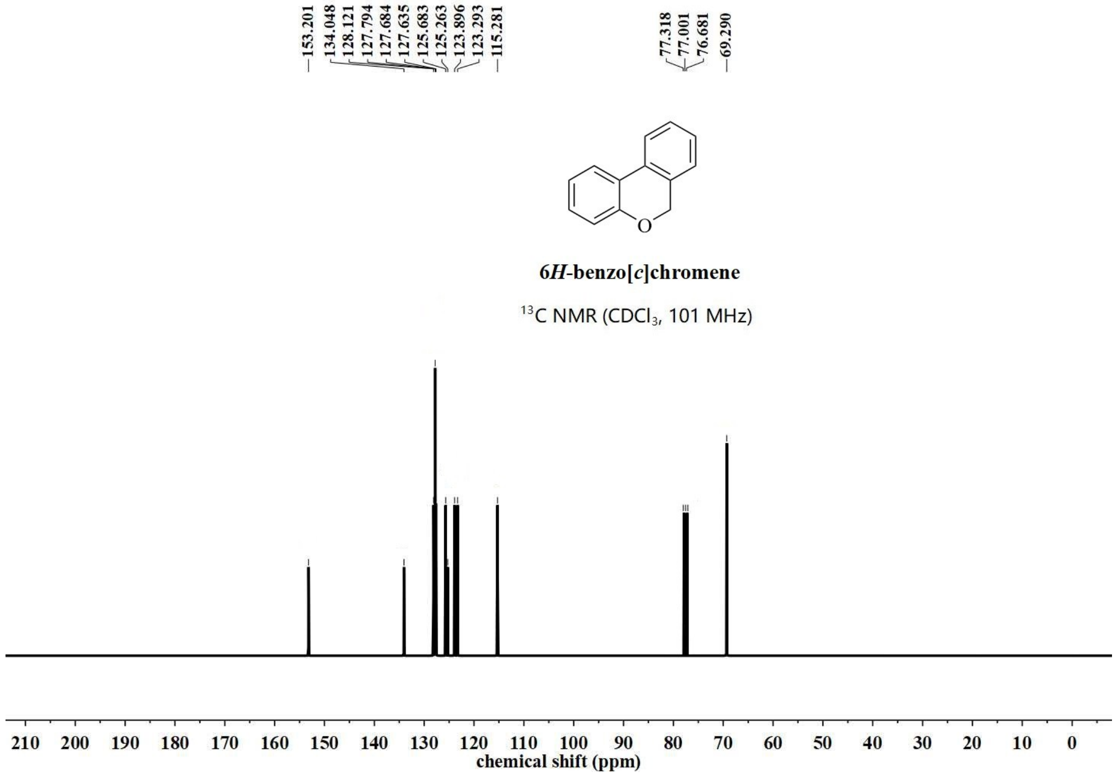
153.201 134.048 128.121 127.794 127.684 127.635 125.683 125.263 123.896 123.293 115.281
77.318 77.001 76.681 69.290
O
6H-benzo[c]chromene
13C NMR (CDCl3, 101 MHz)
210 200 190 180 170 160 150 140 130 120 110 100 90 80 70 60 50 40 30 20 10 0
chemical shift (ppm)
